# Assessing the risk of adverse drug events from combining aromatase inhibitors with CDK4/6 inhibitors using the FAERS and JADER databases

**DOI:** 10.3389/fmed.2026.1823079

**Published:** 2026-07-06

**Authors:** Zhipeng Fan, Peikai Sun, Lei Li

**Affiliations:** 1 Department of Oncology, Wuhan Asia General Hospital affiliated to Wuhan University of Science and Technology, Wuhan, Hubei, China; 2 Department of Radiation and Medical Oncology, Zhongnan Hospital of Wuhan University, Wuhan, Hubei, China

**Keywords:** adverse drug events, aromatase inhibitors, cyclin-dependent kinase 4 and 6 inhibitors, disproportionality analysis, FAERS, JADER

## Abstract

**Background:**

Reports of adverse drug events (ADEs) involving aromatase inhibitors (AIs) and cyclin-dependent kinase 4/6 (CDK4/6) inhibitors exist, but a comprehensive assessment of their combined safety in real-world practice has not been conducted.

**Methods:**

The U.S. Food and Drug Administration Adverse Event Reporting System (FAERS) and the Japanese Adverse Drug Event Report (JADER) database were used to collect data from the first quarter of 2015 to the third quarter of 2025. Disproportionality analysis was performed to identify ADEs associated with combination therapy. Multivariate logistic regression was used to explore factors associated with designated serious outcomes.

**Results:**

A total of 28,495 reports related to combination therapy were included in FAERS. Disproportionality analysis identified 150 ADEs. The three most frequently reported ADEs were fatigue, neutropenia, and white blood cell count decreased. Signals not mentioned in drug labels were found, like skin hypopigmentation, vertigo positional, and plicated tongue. Combination therapy showed stronger reporting signals for some ADEs, including neutropenia, anaemia, and leukopenia. Among 1,186 reports in the JADER database, we identified 25 positive signals, including neutropenia, alanine aminotransferase increased, and aspartate aminotransferase increased. Multivariate logistic regression showed that body weight was associated with the occurrence of serious outcomes and hematological toxicity.

**Conclusion:**

This study not only identified key signals that align with earlier clinical trials but also detected several ADEs not listed in drug labeling. Concurrently, combination therapy was associated with stronger disproportionality signals for certain specific ADEs compared with monotherapy. The findings provided clinicians crucial insights and emphasized the importance of additional research centered on causality to verify the outcomes observed.

## 1 Introduction

Breast cancer ranks as the most frequent malignant tumor among women across the globe and is a significant contributor to cancer-related deaths (1). Over the past decade, its incidence has steadily increased (2). Based on the expression status of hormone receptors (HR) and human epidermal growth factor receptor 2 (HER2), breast cancer can be classified into multiple molecular subtypes. Among these, the HR-positive/HER2-negative (HR+/HER2−) subtype is the most common, accounting for approximately 70% of all cases (3). This subtype typically has a favorable prognosis due to relatively slow tumor growth and a certain degree of responsiveness to endocrine therapy (ET) (4).

HR+ breast cancer cells have receptors for estrogen or progesterone, which makes them responsive to hormonal signals and leads to abnormal proliferation (5). The high occurrence of breast cancer in postmenopausal women is largely due to elevated estrogen levels (6). Therefore, inhibiting estrogen synthesis or blocking its signaling pathways has become a core therapeutic strategy for HR+ breast cancer. Aromatase, a member of the cytochrome P450 family, primarily converts androgens into estrogens in postmenopausal women and serves as the pivotal rate-limiting enzyme in estrogen biosynthesis (7). Aromatase inhibitors (AIs) specifically suppress the activity of the enzyme, blocking estrogen production and thereby inhibiting tumor growth. The third-generation AIs, such as letrozole, anastrozole, and exemestane, have significantly improved efficacy and selectivity compared to earlier generations and have become essential components of HR+ breast cancer treatment. Clinical studies confirmed that these third-generation AIs outperform tamoxifen in tumor control and extend progression-free survival, while also lowering the risk of recurrence and death in postmenopausal patients with HR+ breast cancer (8, 9).

Although ET serves as the standard systemic treatment for HR+ breast cancer by inhibiting tumor progression through disruption of estrogen signaling pathways, its efficacy remains limited in some patients due to primary or acquired resistance (10, 11). Fortunately, the emergence of cyclin-dependent kinase 4/6 (CDK4/6) inhibitors in recent years has greatly transformed the treatment strategies for HR+ breast cancer. These inhibitors have been shown to overcome resistance to traditional ET, improve patient prognosis, and have become a crucial therapeutic option for metastatic HR+/HER2− breast cancer (12). CDK4/6 inhibitors exert their function through the regulation of the G1 to S phase transition, playing a central role in controlling tumor proliferation. CDK4/6 inhibitors selectively suppress the activity of the key regulatory factor CDK4/6, reinstating normal cell cycle regulation and thereby inhibiting tumor growth (13). Multiple clinical trials have confirmed their significant efficacy treating HR+/HER2- breast cancer patients (14, 15).

Currently, CDK4/6 inhibitors have been approved for use in combination with ET as first-line therapy for metastatic HR+/HER2− breast cancer. However, despite the apparent clinical benefits of the combination therapy, its associated adverse drug events (ADEs) cannot be overlooked. Common ADEs to the third-generation AIs include hot flush, musculoskeletal pain, and osteoporosis (16). CDK4/6 inhibitors primarily manifest toxicities such as neutropenia, leukopenia, nausea, and fatigue (17). Randomized controlled trials indicated that combination therapy might lead to ADEs such as neutropenia, leukopenia, fatigue, and electrocardiogram QT prolonged, with severe cases potentially leading to pulmonary embolism and venous thromboembolism (18, 19). However, due to strict patient selection criteria, fixed treatment regimens, and limited follow-up periods, randomized trials struggle to fully reflect the real-world safety profile of combination therapy. For instance, a review of three landmark phase III trials: PALOMA-2 (palbociclib+letrozole), MONALEESA-2 (ribociclib+letrozole), and MONARCH-2 (abemaciclib+fulvestrant) reported that insomnia was the most common psychiatric adverse event, with rates ranging from 9.52% to 16.22% (20). In contrast, a real-world multicountry survey study reported that among patients receiving CDK4/6 inhibitors, 50% of them experienced low sexual, demonstrating discrepancies compared to randomized trials (21).

The constraints of randomized controlled trials in recognizing safety issues make real-world evidence from post-marketing monitoring vital for spotting ADEs. Spontaneous reporting systems, including the U.S. Food and Drug Administration Adverse Event Reporting System (FAERS) and the Japanese Adverse Drug Event Report (JADER) database, offer extensive real-world datasets for pharmacovigilance studies. Previous studies have predominantly focused on single drugs or drugs within the same class, with limited real-world safety analyses of combination therapy involving AIs and CDK4/6 inhibitors (16, 22, 23). This study utilized the FAERS and JADER databases to carry out a comprehensive pharmacovigilance assessment of combination therapy with AIs and CDK4/6 inhibitors, identifying and analyzing safety signals, including ADEs not yet widely recognized. The findings were expected to provide clinicians, patients, and researchers with reference information on the safety of combination therapy with AIs and CDK4/6 inhibitors, thereby optimizing treatment strategies and improving patient outcomes.

## 2 Materials and methods

### 2.1 Data source

FAERS is a global spontaneous reporting database managed by the Food and Drug Administration, extensively collecting ADEs and medication error reports from healthcare professionals, consumers, and manufacturers. It includes seven distinct datasets: patient demographic records, drug details, adverse events, patient outcomes, sources of reports, therapy initiation and discontinuation dates, and drug indication data. JADER is a free post-marketing surveillance database in Japan that contains information on patient demographics, drug use, ADEs, and primary diseases. The retrieval and collection of individual reports were conducted according to the standards advised by FAERS. Cases with identical “primary id” were marked as duplicates, and only the most comprehensive report was kept. If demographic or clinical information was repeated across reports, the dataset selected the most current or detailed entry. All ADEs were standardized and coded using the Medical Dictionary for Regulatory Activities version 28.0 and classified into Preferred Term (PT) and corresponding System Organ Classes (SOC) according to their hierarchical structure. We focused exclusively on drug-induced adverse events, excluding non-indicated use, product issues, medication errors, and disease-related adverse events from analysis. AIs included in the study were letrozole, anastrozole, and exemestane. CDK4/6 inhibitors included palbociclib, ribociclib, abemaciclib, and dalpiciclib. For both FAERS and JADER, drug names were retrieved from the Kyoto Encyclopedia of Genes and Genomes database and standardized with MeSH terms.

ADEs were classified into 4 categories: primary suspected (PS), second suspected (SS), concomitant (C), and interacting (I). Combination therapy was defined as the presence of both an AI and a CDK4/6 inhibitor in the same report, with either drug designated as the PS. If one drug was recorded as PS and the other as SS, C, or I, the case was included in the analysis. Reports in which neither drug was designated as PS were excluded. Due to the large number of cases with missing complete therapy start and end dates in the FAERS and JADER databases, this definition represented report-level co-exposure rather than verified treatment overlap in each individual case. The time to onset (TTO) of ADEs was described as the time span from the start date of drug treatment (START_DT) to the date of ADEs occurrence (EVENT_DT). When START_DT, the end date of drug treatment (END_DT), and EVENT_DT were available, we checked temporal plausibility and excluded records in which the ADEs occurred before treatment initiation from TTO analysis. Reports with missing or incomplete therapy dates were retained for disproportionality analysis but excluded from TTO analyses requiring complete temporal information. Using the scale parameter (*α*) and shape parameter (β), the Weibull distribution test defined the pattern of ADEs over time. The Kruskal-Wallis and Wilcoxon rank-sum test was employed to compare TTO among various groups.

The start date of Q1 2015 was selected because palbociclib, the first CDK4/6 inhibitor approved globally, received the U.S. Food and Drug Administration (FDA) approval in February 2015. This allowed capture of the entire post-marketing period for all CDK4/6 inhibitors included in this study. Although the three AIs (letrozole, anastrozole, exemestane) were approved before 2015, restricting the analysis to the same 2015–2025 window ensures temporal comparability between combination therapy and monotherapy groups. The approval or marketing dates for each drug were provided in Supplementary Table S1. We removed reports originating from Japan from the FAERS database to prevent duplicate counting. We focused on reports mentioning “Breast Cancer” and excluded those with vague or irrelevant indications to identify relevant cases. Serious outcomes included death (DE), disability (DS), hospitalization - initial or prolonged (HO), life-threatening (LT), required intervention to prevent permanent impairment/damage (RI), and other (OT).

### 2.2 Disproportionality analysis

This study employed disproportionality analysis as the signal detection method for pharmacovigilance assessment. Three approaches were utilized for disproportionality analysis when defining and identifying ADEs: reporting odds ratios (ROR) (24), proportional reporting ratios (PRR) (25), and bayesian confidence propagation neural network (BCPNN) (26). ROR and PRR are frequentist disproportionality measures derived from two-by-two contingency tables (27, 28), whereas BCPNN is a Bayesian shrinkage-based method that may reduce the instability of estimates in sparse reporting cells. Because no single algorithm is universally optimal across different reporting frequencies, event types, and database structures, an ADE was considered a positive signal when it met the predefined threshold in at least two of the three algorithms (Supplementary Tables S2, S3). This prespecified criterion was used to reduce method-specific false-positive findings compared with reliance on a single algorithm, while preserving sensitivity for rare but clinically relevant ADEs compared with requiring concordance across all three algorithms. Similar multi-algorithm concordance approaches were employed in previous pharmacovigilance studies (29, 30). To minimize the risk of false positives (type I errors), we applied the Bonferroni method to adjust for multiple *p*-value comparisons, thus striking a balance between controlling false positives and maintaining sensitivity, while also ensuring signal specificity (31). To compare the ADEs reporting signals between the combination therapy group and the monotherapy groups, we calculated the ROR (Supplementary Table S4) and presented the results as forest plots. Drug labels were defined as the regulatory-approved prescribing information for each individual drug. For all drugs except dalpiciclib, we used the U.S. FDA-approved prescribing information. For dalpiciclib, we used the prescribing information approved by the China National Medical Products Administration. A PT was classified as an unexpected signal if it did not appear on the drug label of any of the included drugs.

### 2.3 Drug–drug interaction (DDI) analysis

The evaluation of DDI signals involved four models: (1) the Ω shrinkage measure model (32), (2) the additive model (33), (3) the multiplicative model (34), and (4) the chi-square statistics model (χ^2^) (35). Detailed descriptions of the four models were provided in the Supplementary Table S5. The Ω shrinkage measure provides a shrinkage-based observed-to-expected estimate and reduces instability in sparse cells. The additive and multiplicative models assess whether co-reporting exceeds expected patterns under different null assumptions. And the chi-square model evaluates departure from independence. Because DDI detection in spontaneous reporting systems is particularly susceptible to confounding by indication, concomitant medications, reporting bias, and disease severity (34, 36, 37), we used the DDI analysis as a secondary, high-specificity prioritization step. Therefore, a positive DDI signal was defined only when all four models exceeded their respective thresholds.

### 2.4 Subgroup and sensitivity analysis

Subgroup analyses were performed in FAERS database according to age (<65, ≥65 years) and body weight (<70, ≥70 kg). Missing age or weight values were excluded only from the corresponding subgroup analysis. Sensitivity analysis were conducted by (1) excluding reports containing additional antineoplastic agents other than the AIs-CDK4/6 inhibitors combination, and (2) restricting the data set to reports submitted by health professional, physician, other health professional, pharmacist. All subgroup and sensitivity analysis were performed using the same disproportionality algorithms and signal detection criteria as the primary analysis.

### 2.5 Logistic regression analysis

Logistic regression was performed to explore factors associated with serious outcomes and hematologic toxicity. Serious outcome included DE, DS, HO, LT, RI, and OT. Hematologic toxicity was defined as the presence of at least one hematologic PT, including neutropenia, leukopenia, anaemia, neutrophil count decreased, haemoglobin decreased, or thrombocytopenia. Independent variables included age group (<65 years, ≥65 years), body weight group (<70 kg, ≥70 kg), duration of medication (<1 year, 1–2 years, >2 years), and concomitant chemotherapy status when available. Reports with missing values for independent variables were excluded from the logistic regression analysis. Odds ratio (OR) and 95% confidence interval (CI) for all independent variables were estimated using univariable and multivariable logistic regression.

### 2.6 Statistical analysis

Due to limited baseline patient information in the JADER database, we performed subgroup, sensitivity, TTO, and logistic regression analyses only in the FAERS database. For the TTO and logistic regression analyses, *p* value < 0.05 was considered statistically significant. All analyses were performed using R software version 4.3.1. Data import, cleaning, deduplication, drug name standardization, and case selection were carried out using base R along with the data.table, dplyr, tidyr, stringr, and lubridate packages. Disproportionality analysis and DDI modeling were implemented using custom R scripts based on the formulas provided in Supplementary Tables S2–S5. Logistic regression was performed using the finalfit function from the finalfit package, and the ggplot2 package was used for data visualization. The flowchart of the entire study was presented in Supplementary Figure 1.

## 3 Results

### 3.1 Descriptive analysis

FAERS data was collected over a 10-year period, from Q1 2015 to Q3 2025. Ultimately, we gathered relevant reports for the AIs monotherapy group (*N* = 11,378), the CDK4/6 inhibitors monotherapy group (*N* = 47,457), and the combination therapy group (*N* = 28,495). The clinical characteristics of these reports were summarized in Table 1. Patients were grouped into three age cohorts using 18 and 65 years old as cutoffs. The proportion of patients aged 65 years and older was highest in the AIs monotherapy group (41.6%), followed by the CDK4/6 inhibitors monotherapy group (42.5%) and the combination therapy group (40.6%). Across all three treatment groups, the frequency distribution of patients who weighed under or above 70 kg was similar. After excluding other and missing outcomes, HO was the most frequently reported serious outcome. The reports primarily originated in developed countries (e.g., the United States, France, Great Britain, and Germany). Over one-third of reports in the AIs monotherapy and combination therapy groups were submitted by consumers, whereas the number of reports submitted by consumers exceeded half (*n* = 24,580) in the CDK4/6 inhibitors monotherapy group.

**Table 1:** Demographic and clinical information of reports associated with AIs, CDK4/6 inhibitors, and combination therapy from the FAERS Database.

Characteristics	Report number, n (%)		
	AIs	CDK4/6 inhibitors	Combination therapy
Numbers	11,378	47,457	28,495
Gender			
Female	10,658 (93.7)	45,606 (96.1)	27,537 (96.6)
Male	76 (0.7)	606 (1.3)	290 (1.0)
Unknown or missing	644 (5.7)	1,245 (2.6)	668 (2.3)
Age (years)			
<18	3 (0.0)	13 (0.0)	4 (0.0)
18–64	4,042 (35.5)	17,405 (36.7)	10,816 (38.0)
>64	4,737 (41.6)	20,146 (42.5)	11,557 (40.6)
Unknown or missing	2,596 (22.8)	9,893 (20.8)	6,118 (21.5)
Weight (kg)			
<70	2,264 (19.9)	5,315 (11.2)	5,944 (20.9)
≥70	1887 (16.6)	5,173 (10.9)	6,248 (21.9)
Unknown or missing	7,227 (63.5)	36,969 (77.9)	16,303 (57.2)
Serious outcome#			
CA	1 (0.0)	3 (0.0)	4 (0.0)
DE	765 (6.7)	6,586 (13.9)	3,036 (10.7)
DS	432 (3.8)	139 (0.3)	145 (0.5)
HO	2,374 (20.9)	6,905 (14.6)	6,108 (21.4)
LT	334 (2.9)	460 (1.0)	780 (2.7)
OT	5,437 (47.8)	12,094 (25.5)	11,929 (41.9)
RI	10 (0.1)	99 (0.2)	12 (0.0)
Missing	2025 (17.8)	21,171 (44.6)	6,481 (22.7)
Reported Countries (Top five)			
United States	3,678 (32.3)	34,019 (71.7)	12,541 (44.0)
Germany	597 (5.2)	-	3,899 (13.7)
France	1,346 (11.8)	-	1,376 (4.8)
Argentina	-	2018 (4.3)	1,166 (4.1)
Canada	-	-	1,063 (3.7)
Great Britain	1,190 (10.5)	-	-
Japan	633 (5.6)	827 (1.7)	-
India	-	1,665 (3.5)	-
Country not specified	-	1,041 (2.2)	-
Reported Person			
Consumer	3,756 (33.0)	24,580 (51.8)	10,810 (37.9)
Health Professional	1,331 (11.7)	5,788 (12.2)	4,682 (16.4)
Physician	3,510 (30.8)	6,917 (14.6)	8,399 (29.5)
Other health-professional	1,373 (12.1)	4,429 (9.3)	2,270 (8.0)
Pharmacist	973 (8.6)	4,497 (9.5)	1,696 (6.0)
Missing	435 (3.8)	1,246 (2.6)	638 (2.2)

AIs, aromatase inhibitors; CDK4/6, cyclin-dependent kinase 4/6; #, Outcome categories were mutually exclusive: each report contributed to only one serious outcome; CA, congenital anomaly; DE, death; DS, disability; HO, hospitalisation-initial or prolonged; LT, life-threatening; OT, other; RI, required intervention to prevent permanent impairment/damage.

**Figure 1:**
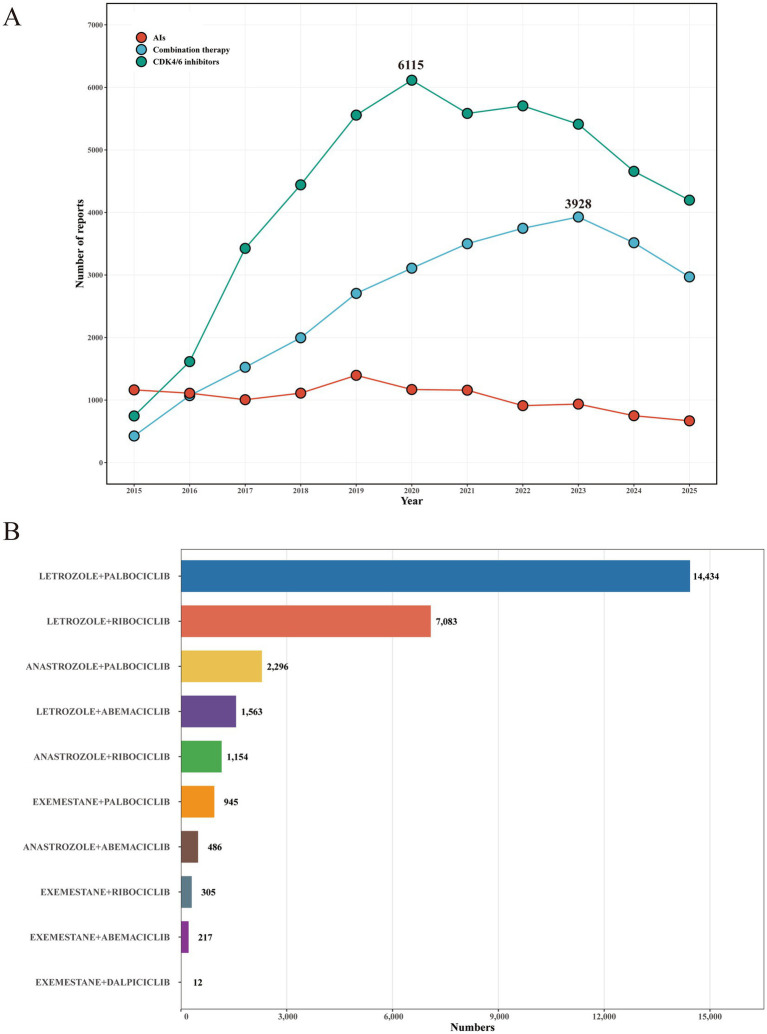
ADEs signal monitoring results. **(A)** The number of case reports each year from Q1 2015 through Q3 2025. **(B)** The number of drug combinations for combination therapy. ADEs, adverse drug events.

Regarding the annual distribution of report numbers (Figure 1A), the number of reported cases in the AIs monotherapy group showed a gradual downward trend. From 2015 to 2023, the number of cases in the combination therapy group continued to increase, reaching a peak of 3,928 reported cases in 2023. In contrast, the growth trend in the CDK4/6 inhibitors monotherapy group reversed starting in 2020. Figure 1B further illustrated the specific drug combinations used in the combination therapy group. The letrozole plus palbociclib regimen was the most widely used, involving 14,434 patients, while the exemestane plus dalpiciclib regimen was the least common, reported in only 12 patients. To further examine drug-specific reporting patterns, annual report trends stratified by individual CDK4/6 inhibitors, individual AIs, and major AIs-CDK4/6 inhibitors combinations were provided in Supplementary Figure 2. The results revealed substantial heterogeneity in the number of ADEs reports across different drugs. Among combination therapy regimens, the combination of palbociclib and letrozole ranked first in the number of reported cases (Supplementary Figure 2A). In the AIs monotherapy group, letrozole had the highest number of reported cases (584 cases, Supplementary Figure 2B), whereas in the CDK4/6 inhibitors monotherapy group, palbociclib was the most prominent, with 4,607 reported cases (Supplementary Figure 2C).

### 3.2 Signal monitoring at the PT level

**Figure 2:**
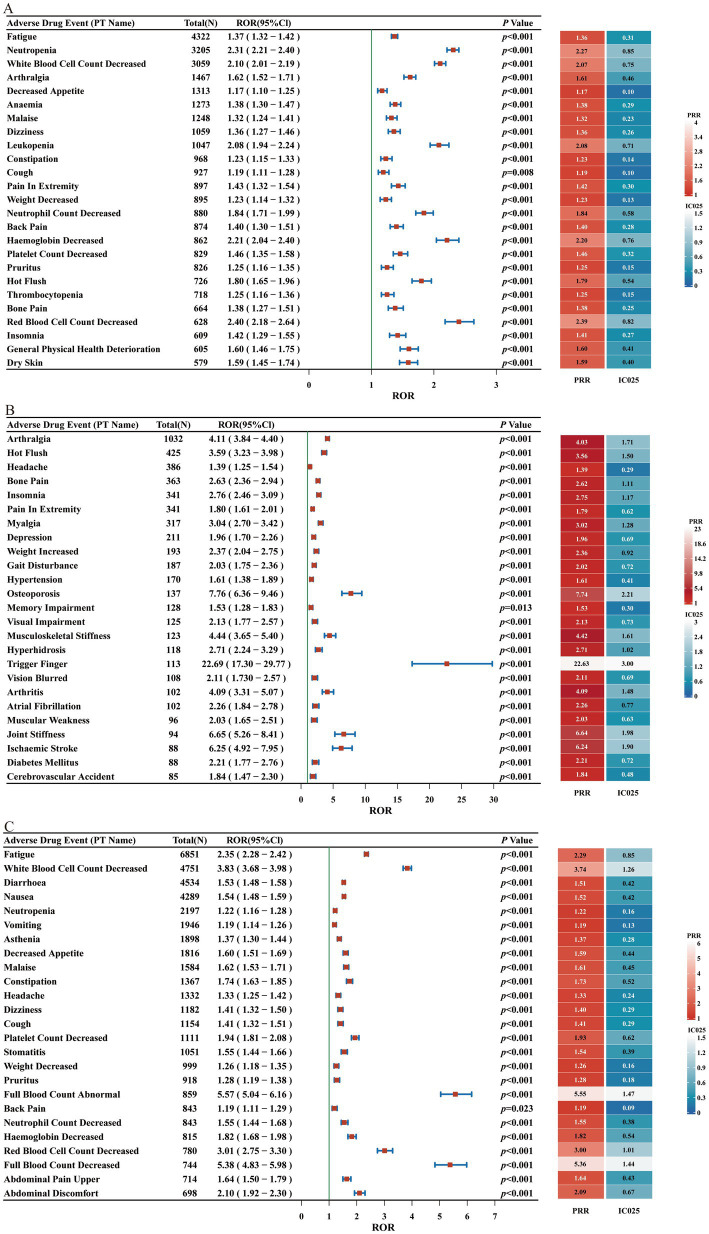
Signal detection at the PT level. **(A)** The combination therapy group. **(B)** The AIs monotherapy group. **(C)** The CDK4/6 inhibitors monotherapy group. PT, preferred term; AIs, aromatase inhibitors; CDK4/6, cyclin-dependent kinase 4/6; ROR, Reporting Odds Ratio; CI, confidence interval; PRR, Proportional Reporting Ratio; IC, Information Component; IC025, the lower limit of the 95% one-sided CI, of the IC.

After excluding PTs related to symptoms associated with the indication and those clearly unrelated to the drug, we identified 162, 104, and 150 PTs in the AIs monotherapy, CDK4/6 inhibitors monotherapy, and combination therapy groups, respectively. The complete list of PTs was provided in Supplementary Tables S6–S8. Ranked by reporting frequency, forest plots displayed the ROR values and 95% CI for the top 25 signals in each cohort. The top three signals by case counts in the combination therapy cohort were fatigue (*n* = 4,322, ROR: 1.37 [1.32–1.42], PRR: 1.36, IC025: 0.31), neutropenia (*n* = 3,205, ROR: 2.31 [2.21–2.40], PRR: 2.27, IC025: 0.85), and white blood cell count decreased (*n* = 3,059, ROR: 2.10 [2.01–2.19], PRR: 2.07, IC025: 0.75) (Figure 2A). In the AIs monotherapy group, the top three PTs were arthralgia (*n* = 1,032, ROR: 4.11 [3.84–4.40], PRR: 4.03, IC025: 1.71), hot flush (*n* = 425, ROR: 3.59 [3.23–3.98], PRR: 3.56, IC025: 1.50), and headache (*n* = 386, ROR: 1.39 [1.25–1.54], PRR: 1.39, IC025: 0.29), but trigger finger (*n* = 113, ROR: 22.69 [17.30–29.77], PRR: 22.63, IC025: 3.00) and osteoporosis (*n* = 137, ROR: 7.76 [6.36–9.46], PRR: 7.74, IC025: 2.21) showed stronger signals (Figure 2B). In contrast, the CDK4/6 inhibitors monotherapy group exhibited stronger signals for full blood count abnormal (*n* = 859, ROR: 5.57 [5.04–6.16], PRR: 5.55, IC025: 1.47) and full blood count decreased (*n* = 744, ROR: 5.38 [4.83–5.98], PRR: 5.36, IC025: 1.44). Fatigue (*n* = 6,851, ROR: 2.35 [2.28–2.42], PRR: 2.29, IC025: 0.85), white blood cell count decreased (*n* = 4,751, ROR: 3.83 [3.68–3.98], PRR: 3.74, IC025: 1.26), and diarrhoea (*n* = 4,534, ROR: 1.53 [1.48–1.58], PRR: 1.51, IC025: 0.42) were the most common (Figure 2C).

The three treatment groups shared four common PTs: hot flush, insomnia, gait disturbance, and memory impairment. The AIs monotherapy group and CDK4/6 inhibitors monotherapy group had 144 and 52 unique PTs, respectively, while the combination therapy group had 91 unique PTs. Among these 91 unique PTs (Supplementary Table S9), the most common were anaemia, leukopenia, thrombocytopenia, general physical health deterioration, urinary tract infection, and pleural effusion. Moreover, our data mining found some intense PT signals not identified in the drug labels of AIs and CDK4/6 inhibitors, such as skin hypopigmentation, vertigo positional, red blood cells urine positive, and plicated tongue, revealing that combination therapy induced novel, previously unrecognized PTs.

### 3.3 Combination therapy against monotherapy

**Figure 3:**
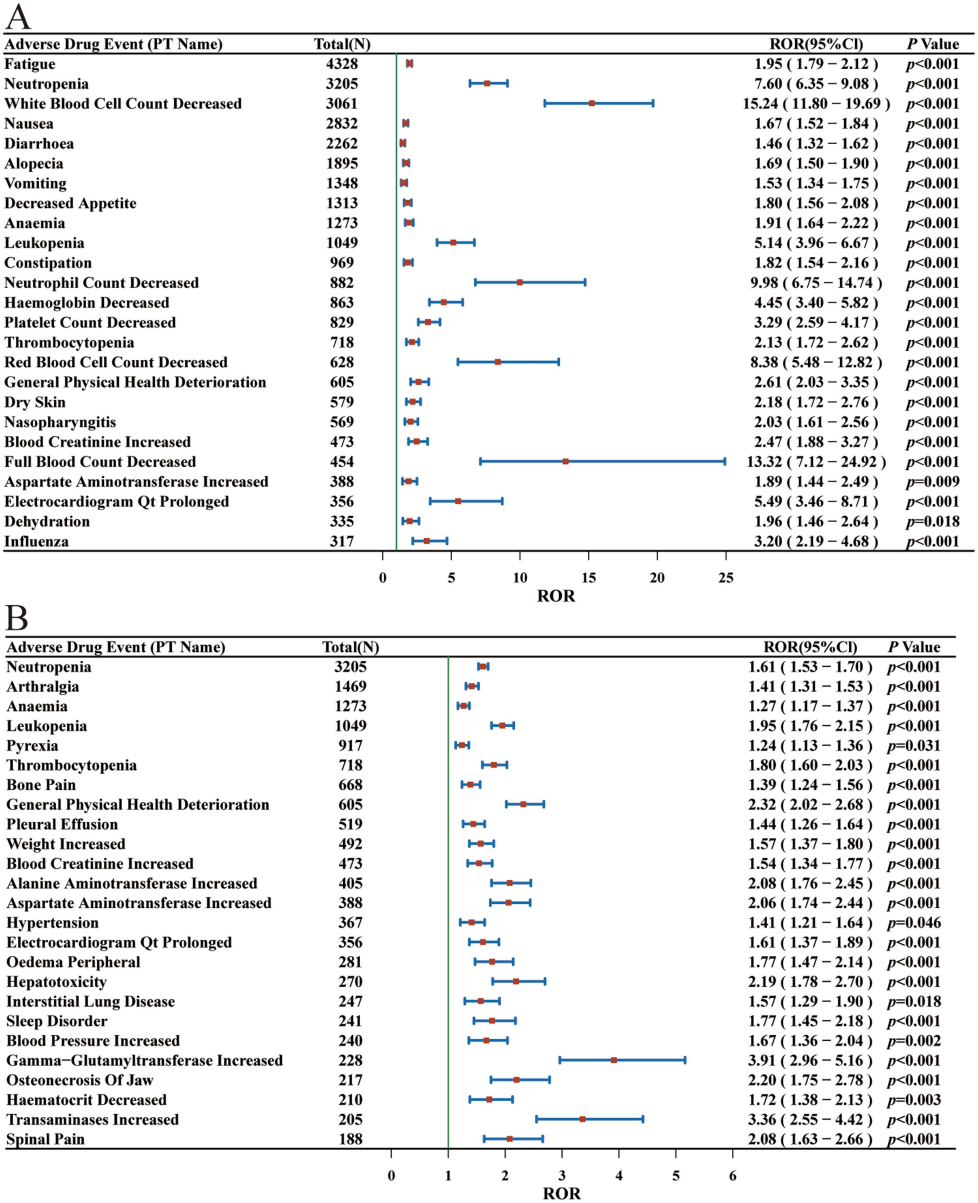
Signal detection for combination therapy against monotherapy. **(A)** Combination therapy vs. AIs monotherapy. **(B)** Combination therapy vs. CDK4/6 inhibitors monotherapy. AIs, aromatase inhibitors; CDK4/6, cyclin-dependent kinase 4/6; PT, preferred term; ROR, Reporting Odds Ratio; CI, confidence interval.

The ROR algorithm was used to compare reporting associations between combination therapy and monotherapy (AIs monotherapy or CDK4/6 inhibitors monotherapy). The results (Supplementary Tables S10, S11) showed that compared with either monotherapy group, the combination therapy group was associated with stronger reporting signals for certain ADEs. Specifically, compared with AIs monotherapy, the combination therapy group showed stronger reporting signals for fatigue, neutropenia, white blood cell count decreased, nausea, and diarrhoea (Figure 3A). Compared with CDK4/6 inhibitors monotherapy, the combination therapy group showed stronger reporting signals for neutropenia, arthralgia, anaemia, leukopenia, and thrombocytopenia (Figure 3B). These exploratory findings indicated that combination therapy was associated with stronger disproportionality signals for specific ADEs relative to monotherapy.

### 3.4 Evaluating DDI using statistical models

Previous ROR algorithm results showed that combination therapy was associated with stronger reporting signals for certain ADEs already observed with monotherapy. To further explore this pattern, we performed four DDI statistical models to assess potential associations between AIs-CDK4/6 inhibitors and ADEs. A total of 51 positive PT signals met the thresholds of all four models (Supplementary Table S12). DDI signals were detected for several common PTs, including neutropenia, anaemia, leukopenia, neutrophil count decreased, haemoglobin decreased, thrombocytopenia, alanine aminotransferase increased, aspartate aminotransferase increased, and hepatotoxicity (Table 2). These results suggested that combination therapy showed stronger disproportionality signals for certain specific ADEs, indicating potential drug-interaction reporting patterns.

**Table 2:** Analysis of potential drug interactions between AIs and CDK4/6 inhibitors.

PTs	*n*	Ω shrinkage model		Additive model		Multiplicative model		Chi-squared model	
Neutropenia	3,205	0.63	P	0.01564	P	11.40	P	26.87	P
Anaemia	1,273	0.26	P	0.00386	P	2.09	P	8.37	P
Leukopenia	1,047	0.87	P	0.00616	P	5.33	P	21.77	P
Neutrophil count decreased	880	0.10	P	0.00334	P	18.05	P	4.01	P
Haemoglobin decreased	862	0.12	P	0.00169	P	10.97	P	4.36	P
Thrombocytopenia	718	0.05	P	0.00471	P	1.31	P	2.84	P
General physical health deterioration*	605	0.39	P	0.00405	P	1.59	P	8.58	P
Decreased immune responsiveness*	550	0.24	P	0.00121	P	154.74	P	5.81	P
Pleural effusion	518	0.12	P	0.00106	P	1.02	P	3.84	P
Blood creatinine increased	473	0.46	P	0.00119	P	4.12	P	8.93	P
Alanine aminotransferase increased	405	0.33	P	0.00195	P	1.11	P	6.55	P
Aspartate aminotransferase increased	388	0.34	P	0.00204	P	1.29	P	6.60	P
Electrocardiogram QT prolonged	356	0.53	P	0.00121	P	13.37	P	9.01	P
Hepatotoxicity	270	0.80	P	0.00186	P	6.44	P	11.28	P
Haematocrit decreased	210	0.58	P	0.00097	P	9.94	P	7.91	P

AIs, aromatase inhibitors; CDK4/6, cyclin-dependent kinase 4/6; PTs, preferred terms; n, the number of reports; P, positive signal; *, unexpected signals that were not listed in the drug label.

### 3.5 Subgroup analysis

**Figure 4:**
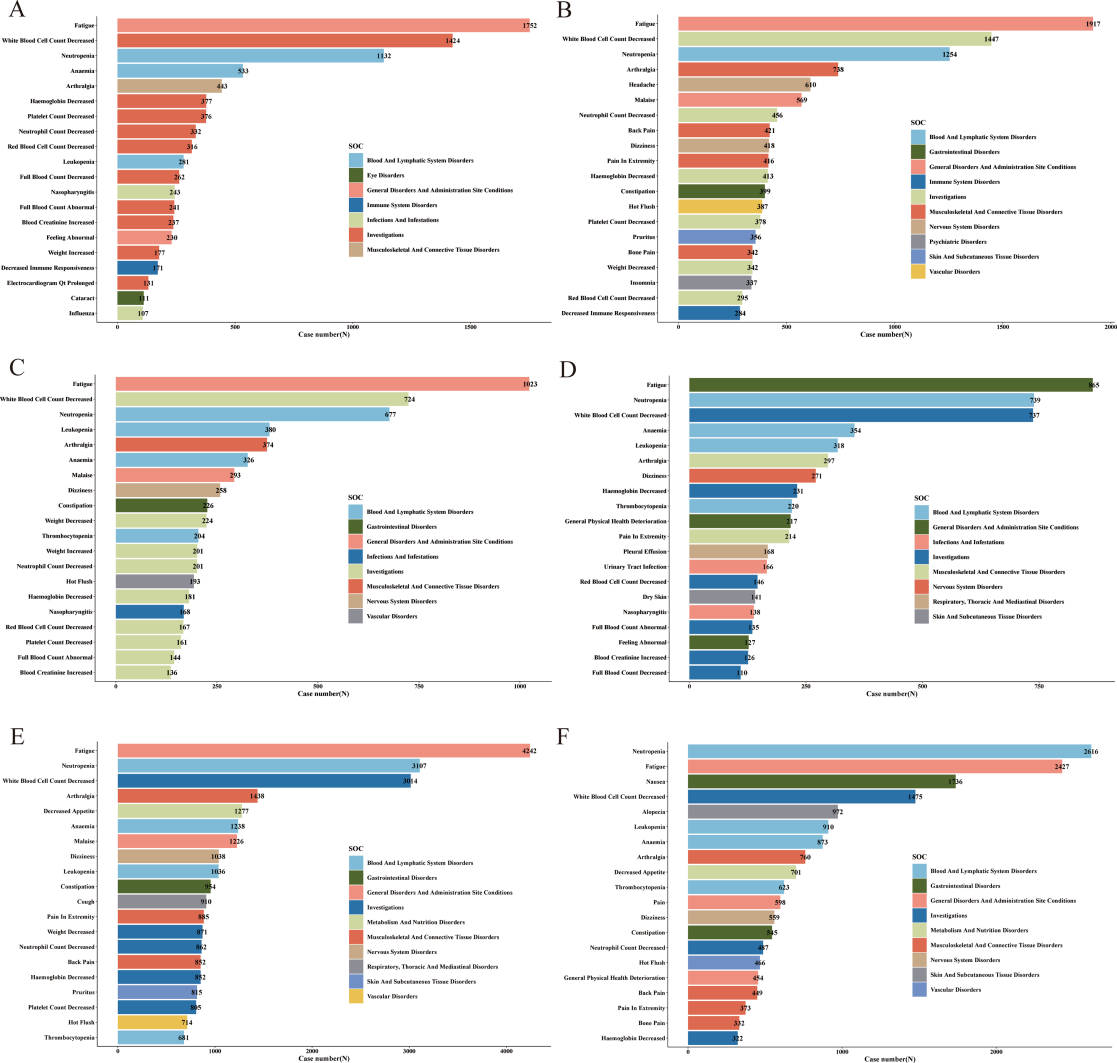
The results of the subgroup and sensitivity analysis. **(A)** Subgroup analysis results from the FAERS database in the population aged≥65 years. **(B)** Subgroup analysis results from the FAERS database in the population aged <65 years. **(C)** Subgroup analysis results from the FAERS database in the population with body weight≥70 kg. **(D)** Subgroup analysis results from the FAERS database in the population with body weight <70 kg. **(E)** Sensitivity analysis results from the FAERS database excluding other antineoplastic agents. **(F)** Sensitivity analysis results from the FAERS database restricted to reports submitted by medical professionals. SOC, system organ class.

Subgroup analysis were carried out according to age and weight demographics to explore differences in the reporting proportions of ADEs associated with combination therapy across distinct populations (Supplementary Tables S13–S16). We screened the 20 most common ADEs. The most prominent ADEs in the elderly population (≥65 years old) were fatigue, white blood cell count decreased, neutropenia, anaemia, and arthralgia (Figure 4A). In contrast, the primary ADEs in the non-elderly population (<65 years old) were fatigue, white blood cell count decreased, neutropenia, arthralgia, and headache (Figure 4B). Similarly, among patients weighing ≥70 kg, fatigue, white blood cell count decreased, neutropenia, leukopenia, and arthralgia were particularly prominent (Figure 4C). For patients under 70 kg, the most prominent ADEs included fatigue, neutropenia, white blood cell count decreased, anaemia, and leukopenia (Figure 4D).

### 3.6 Sensitivity analysis

In clinical practice, some patients with breast cancer are treated with a combination of other antitumor agents, like capecitabine and taxanes, to improve the efficacy of the treatment. To lessen the influence of simultaneous medications on our findings, a sensitivity analysis was carried out. After excluding cases involving combination therapy with other antineoplastic agents, we identified 145 PTs (Supplementary Table S17). Based on positive signal criteria, similar PTs included fatigue, neutropenia, white blood cell count decreased, arthralgia, decreased appetite, anaemia, malaise, dizziness, and leukopenia (Figure 4E). To reduce reporting bias, we restricted adverse reaction reports to healthcare professionals (health professional, physician, other health professional, pharmacist) and collected 97 PTs from FAERS (Supplementary Table S18). Persistent positive signals included neutropenia, fatigue, nausea, white blood cell count decreased, alopecia, leukopenia, anaemia, arthralgia, decreased appetite, and thrombocytopenia (Figure 4F).

### 3.7 TTO analysis

**Figure 5:**
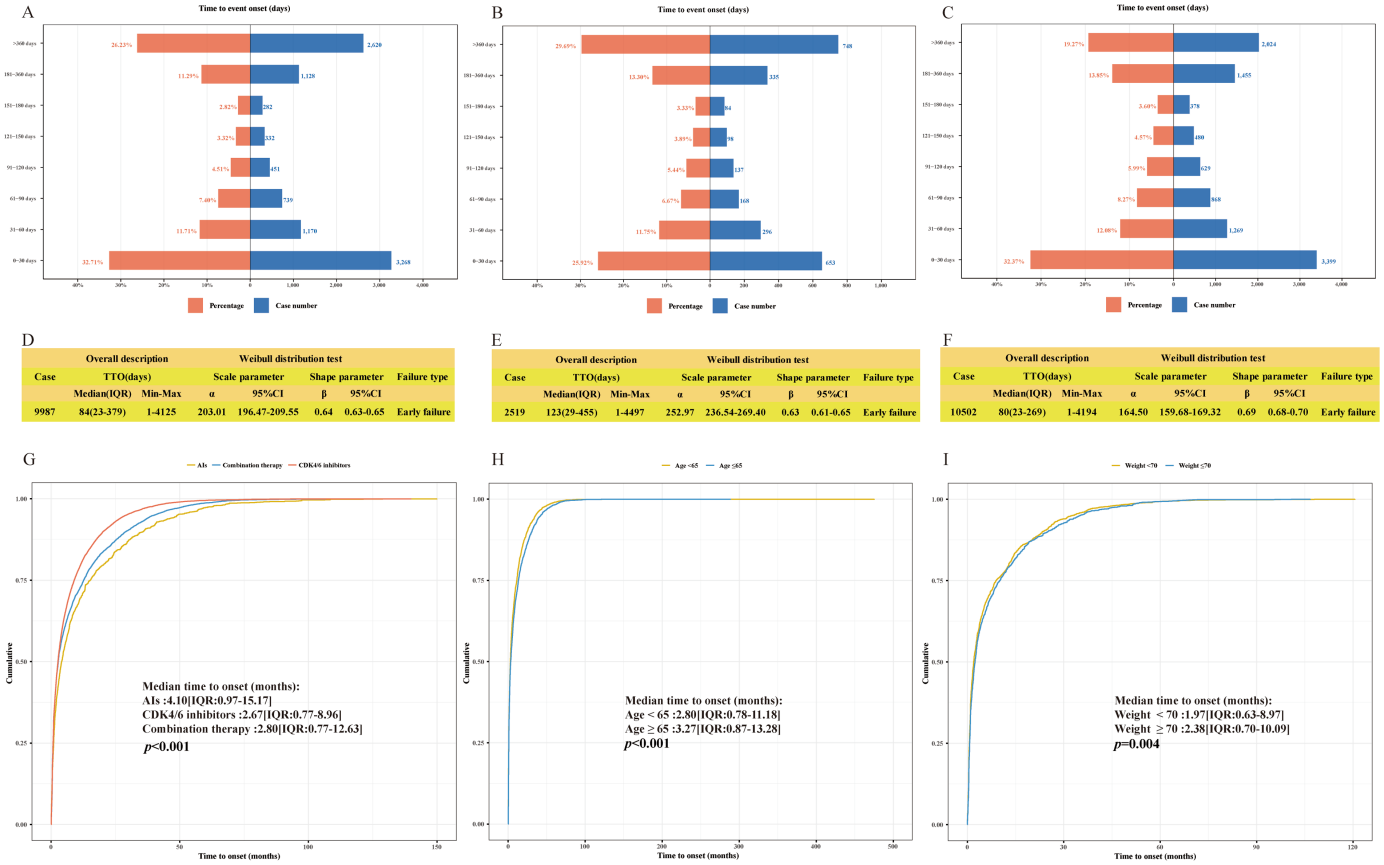
The reports of TTO, Weibull distribution test analysis, and cumulative distribution of ADEs. **(A,D)** The combination therapy group. **(B,E)** The AIs monotherapy group. **(C,F)** The CDK4/6 inhibitors monotherapy group. **(G)** Cumulative distribution of time to ADEs for AIs, CDK4/6 inhibitors, and combination therapy. **(H,I)** Cumulative distribution of time to ADEs for the combination therapy group based on age and weight. TTO, time-to-onset; IQR, interquartile range; CI, confidence interval; ADEs, adverse drug events; AIs, aromatase inhibitors; CDK4/6, cyclin-dependent kinase 4/6.

After excluding reports lacking temporal information, duplicates, and erroneous entries, we retrieved case reports associated with three distinct treatment strategies from the FAERS database. Results indicated (Figures 5A–C) that most ADEs occurred within 1 year, with the highest frequency in all three groups within the first 30 days of treatment (combination therapy group: 32.71%; AIs monotherapy group: 25.92%; CDK4/6 inhibitors monotherapy group: 32.37%). Nevertheless, ADEs could still arise after 1 year of treatment (combination therapy group: 26.23%; AIs monotherapy group: 29.69%; CDK4/6 inhibitors monotherapy group: 19.27%). The Weibull distribution analysis for TTO (Figures 5D–F) displayed that the 95% CI upper limit for *β* was under 1, aligning with an early-failure pattern, indicating that the probability of ADEs across all three groups gradually decreased over time. Significant differences in onset timing were also observed across treatment strategies (Figure 5G, *p* < 0.001). Concurrently, within the combination therapy group, we stratified TTO analysis by age and weight cohorts (Figures 5H,I). We found that ADEs occurred earlier in individuals who were younger than 65 years or who weighed less than 70 kg (*p* < 0.05).

### 3.8 External validation in the JADER database

**Figure 6:**
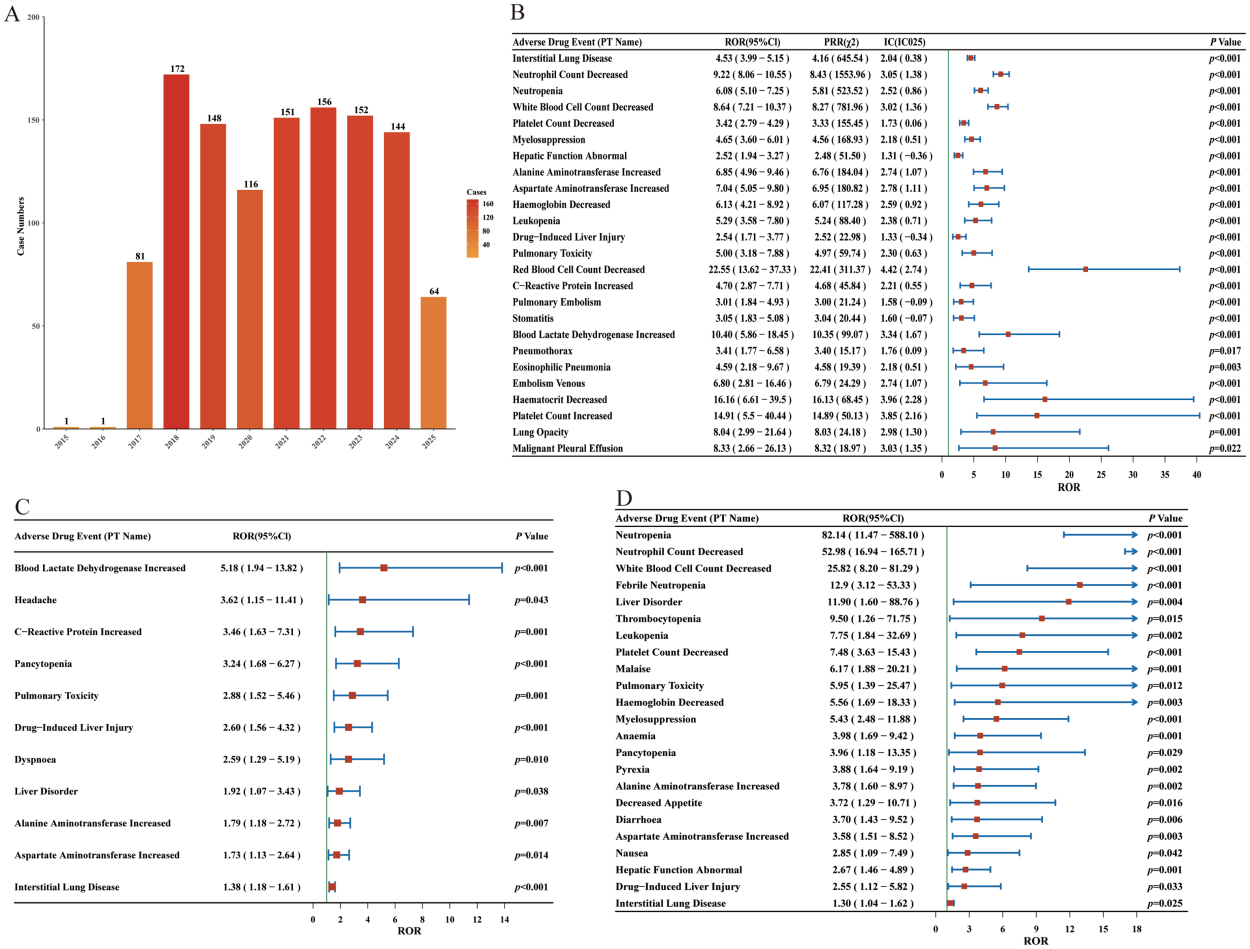
External validation was performed using the JADER database. **(A)** The annual number of case reports submitted for combination therapy in the JADER database. **(B)** Signal detection at the PT level for combination therapy in the JADER database. **(C)** Combination therapy group vs. AIs monotherapy group. **(D)** Combination therapy group vs. CDK4/6 inhibitors monotherapy group. PT, preferred term; ROR, Reporting Odds Ratio; CI, confidence interval; PRR, Proportional Reporting Ratio; IC, information component; IC025, the lower limit of the 95% one-sided CI, of the IC; AIs: aromatase inhibitors; CDK4/6: cyclin-dependent kinase 4/6.

Data from the JADER database was used to validate the results obtained from FAERS. PT level signals were evaluated using the same ROR, PRR, and BCPNN thresholds used for FAERS database. A PT was defined as a positive disproportionality signal when it met the predefined threshold in at least two of the three algorithms. A total of 1,186 case reports related to combination therapy were collected (Figure 6A). In our signal mining study targeting the combination therapy of AIs and CDK4/6 inhibitors, we detected multiple persistently reported and positive disproportionality signals (Figure 6B), including neutrophil count decreased, neutropenia, white blood cell count decreased, haemoglobin decreased, leukopenia, and aspartate aminotransferase increased. These signals aligned with FAERS database analysis results, further validating their reliability. Additionally, we identified several novel potential risk signals that had not received sufficient attention, such as interstitial lung disease, pulmonary embolism, C-reactive protein increased, and embolism venous.

We again compared the reporting signal strength of combination therapy with either AIs monotherapy or CDK4/6 inhibitors monotherapy. Results (Figures 6C,D) showed that combination therapy exhibited stronger reporting signals than either monotherapy for neutropenia, neutrophil count decreased, alanine aminotransferase increased, anaemia, aspartate aminotransferase increased, haemoglobin decreased, and pancytopenia. This observation further supported our prior finding that combination therapy was associated with stronger reporting signals for specific ADEs compared with monotherapy.

### 3.9 Logistic regression analysis

**Figure 7:**
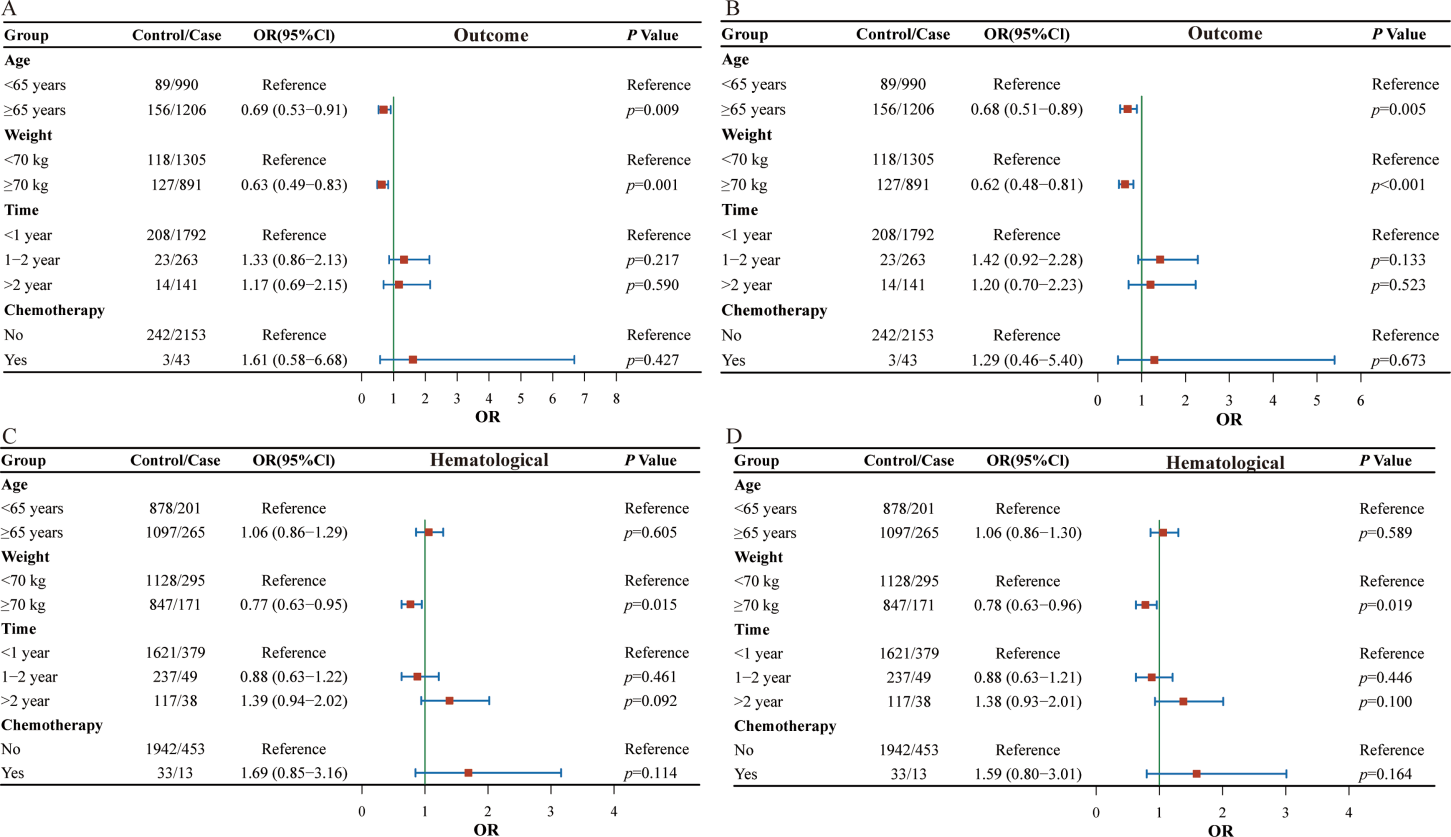
The results of univariate and multivariate logistic regression. **(A,B)** Results of univariate and multivariate logistic regression analysis for serious outcomes. **(C,D)** Results of univariate and multivariate logistic regression analysis for hematologic toxicity. OR, odds ratio; CI, confidence interval.

The occurrence of serious outcomes leads to poor patient prognosis. To explore the factors contributing to serious outcomes, we conducted a logistic regression analysis. The results demonstrated significant associations between age and weight with serious outcomes. Patients aged ≥65 years had a lower risk of serious outcomes than the control group (OR = 0.68, 95% CI = 0.51–0.89, *p* = 0.005). Multivariate logistic regression (Figures 7A,B) confirmed that weight ≥70 kg (OR = 0.62, 95% CI = 0.48–0.81, *p* < 0.001) was a protective factor for serious outcomes. Other demographic characteristics were not significantly associated with serious outcomes.

Given that PTs with higher incidence rates of neutropenia, anaemia, leukopenia, decreased neutrophil count, decreased haemoglobin, and thrombocytopenia all detected positive DDI signals and all fall under hematologic toxicity, we examined their correlation with independent variables. Specifically, only weight ≥70 kg (OR = 0.78, 95% CI = 0.63–0.96, *p* = 0.019) emerged as a protective factor against hematologic toxicity (Figures 7C,D).

## 4 Discussion

Although adverse reactions associated with AIs, CDK4/6 inhibitors, and their combination regimens have been extensively documented and studied in clinical trials, the current literature on real-world evidence remains relatively limited. Certain delayed or rare ADEs are often hard to identify quickly due to the inherent limitations of pre-marketing trials, such as limited sample sizes and short follow-up durations. We conducted the first systematic pharmacovigilance study of ADEs linked to the combination therapy of AIs and CDK4/6 inhibitors, utilizing the FAERS and JADER databases. Through disproportionality analysis, we detected significant imbalance signals in reporting for AIs, CDK4/6 inhibitors, and their combination therapy, suggesting distinct ADEs profiles across treatment regimens. Notably, several adverse reactions already listed in drug labels—such as fatigue, neutropenia, decreased appetite, hot flush, and arthralgia—also appeared frequently in the signal list of this study, which provides empirical support for known safety profiles.

The number of case reports across different drug categories in the FAERS database exhibited variation, primarily influenced by factors such as the time on the market, market utilization, and clinical prevalence. The temporal trend analysis in this study revealed an overall decline in the number of case reports for the AIs monotherapy group, potentially reflecting the evolving shift in breast cancer ET strategies toward the combination therapy. Substantial heterogeneity in reporting volumes was observed across individual drugs and combinations, with palbociclib plus letrozole having the highest number and dalpiciclib the fewest, largely due to market availability and prescribing frequency. Higher report counts do not imply higher clinical risk but reflect exposure and reporting intensity. These variations should be considered when interpreting signal strength, and direct comparisons across drugs with vastly different report volumes should be made cautiously.

Previously published studies about the safety of AIs or CDK4/6 inhibitors have primarily focused on single-agent analyses or specific types of ADEs. For instance, existing literature has specifically examined the adverse reaction profiles of ribociclib monotherapy (38) and letrozole monotherapy (22). Furthermore, Wu et al. (39) and Raschi et al. (40) independently explored the relationship between skin toxicity and AIs and CDK4/6 inhibitors, while Elshafie et al. (41) and Liu et al. (42) investigated cardiovascular events associated with AIs and CDK4/6 inhibitors. In our study, several ADEs identified in these previous investigations also generated positive disproportionality signals, indicating consistency in reporting patterns across different data sources and study designs.

Whether in the FAERS or JADER databases, disproportionality analysis consistently detected stronger reporting signals for hematologic toxicity associated with combination therapy compared to monotherapy. Both the ROR algorithm and the DDI statistical model detected positive signals in PTs for neutropenia, anaemia, and leukopenia. As indicated on drug labels, hematologic toxicity is the most common adverse reaction of CDK4/6 inhibitors, primarily due to their inhibition of CDK6, a key regulator of hematopoietic progenitor cell proliferation (43, 44). Unlike chemotherapy-induced neutropenia, which results from DNA damage and apoptosis, CDK4/6 inhibitors cause neutropenia by reversibly inhibiting the cell cycle (45). Consequently, it typically presents as afebrile and transient, with rapid recovery upon discontinuation, making it clinically manageable (46). In contrast, hematologic toxicity is not listed as a primary adverse reaction in drug labels of AIs, consistent with our screening results. However, study reported that neutropenia was a relatively common event with letrozole and exemestane (16), though direct evidence establishing a causal relationship with AIs remains lacking. While direct real-world comparisons between combination therapy and CDK4/6 inhibitors monotherapy were lacking, current clinical research evidence indicated that patients on combination therapy experienced neutropenia more frequently than those on AIs monotherapy (47). Furthermore, our exploratory comparison demonstrated that the proportion of neutropenia reports was higher in the combination therapy group than in the AIs monotherapy group. These findings suggested that enhanced hematologic monitoring could be considered for patients receiving combination therapy.

This study also found previously underreported hepatotoxicity events associated with AIs and CDK4/6 inhibitors, including hepatic cytolysis and hepatic steatosis. Alanine aminotransferase increased and aspartate aminotransferase increased are common manifestations of hepatotoxicity with CDK4/6i, whereas they are less frequent with AIs monotherapy. The mechanisms underlying hepatotoxicity involve multiple factors, including lipid metabolism disorders, mitochondrial dysfunction, alterations in hepatic metabolic pathways, and inhibition of hepatic transporters (24). Among them, the tendency of abemaciclib to accumulate in hepatocytes due to its high lipophilicity, coupled with ribociclib’s ability to inhibit key hepatic transporter functions, may represent the potential mechanisms through which they induce liver injury (48). Recent case reports revealed that AIs caused liver damage such as acute hepatitis and chronic cholestatic hepatitis (49–51). Liver function typically recovered completely after discontinuation, implying a mechanism potentially related to drug metabolism-mediated hepatocyte injury (52–54). In our analysis, AIs monotherapy was associated with disproportionate reporting of cholestasis, autoimmune hepatitis, and chronic hepatitis. Clinical trial data showed that there was a greater occurrence of hepatotoxicity in patients treated with both ribociclib and letrozole than in those treated with letrozole alone (47). Our findings suggested a signal of stronger reporting associations for liver injury with combination therapy, which might warrant mechanistic investigation. Given that AIs combined with CDK4/6 inhibitors have become the standard treatment for advanced HR+ breast cancer, often requiring long-term use, hepatobiliary toxicity has emerged as a significant complication warranting attention. We strongly recommend establishing a systematic liver function monitoring and follow-up system in clinical practice.

Combination therapy was also closely correlated with ADEs, including electrocardiogram QT prolonged, pleural effusion, decreased immune responsiveness, urinary tract infection, blood creatinine increased, and nasopharyngitis. Existing evidence suggested that ribociclib might induce QT interval prolongation by downregulating the expression of KCNH2 and upregulating the expression of SCN5A and SNTA1 (55). Pleural effusion and decreased immune responsiveness were also frequently observed with ribociclib, with the specific mechanisms not yet fully elucidated (56). Clinical trials reported increased infection rates among patients receiving palbociclib (18). The incidence of infection-related ADEs was higher in the palbociclib plus letrozole group than in the letrozole monotherapy group, primarily involving upper respiratory tract viral infections (57). Urinary tract infections were the most common adverse infection type with ribociclib, predominantly grades 1–2 (14). In contrast, fewer infection-related events were reported with AIs monotherapy. The incidence of neutropenia was significantly elevated in combination therapy groups, primarily attributable to the inclusion of CDK4/6 inhibitors in the regimen. Given that neutropenia is a well-established risk factor for infection, patients in these groups appeared to be at heightened risk of infection. Blood creatinine increased was associated with abemaciclib, a phenomenon attributed to its inhibition of tubular secretory function, without affecting the glomerular filtration rate (58). The CDK4/6 inhibitors component in the combination therapy mainly mediated the aforementioned ADEs. As a result, in clinical practice, physicians should remain highly vigilant regarding the potential association of related ADEs with CDK4/6 inhibitors and promptly initiate clinical intervention.

Additionally, we got several serious ADEs, such as superficial vein thrombosis and pulmonary embolism. Substantial evidence indicated that both AIs and CDK4/6 inhibitors promote the risk of thrombotic events. A pooled analysis of the MONARCH 2 and 3 trials implicated incidence rates of 4.8% for pulmonary embolism and 6.1% for deep vein thrombosis among patients treated with abemaciclib (19). A retrospective study also reported a 9% incidence of venous thromboembolism among 424 patients primarily treated with palbociclib (59). Furthermore, case reports described an acute myocardial infarction occurring 2 weeks after abemaciclib initiation and diffuse venous thrombosis with pulmonary embolism in a patient receiving anastrozole (60–62). Despite the lack of full understanding of how these two types of drugs led to thrombosis, we strongly emphasized the need for continuous monitoring of coagulation function before and during treatment initiation.

In breast cancer drug therapy, neurologic and psychiatric system adverse events represent a category of reactions with low reporting rates but significant clinical importance. In the combination therapy group, positive signals were detected for insomnia, stress, sleep disorders, depressed mood, and memory impairment. In the disproportionality analysis of the AIs monotherapy group, the neurologic and psychiatric systems primarily reported headache, insomnia, depression, and memory impairment. Research demonstrated that breast cancer patients receiving AIs therapy appeared to have higher levels of anxiety and depression, along with diminished cognitive perception capabilities, compared to healthy postmenopausal women (63). Additionally, the incidence of carpal tunnel syndrome was higher among AIs-treated patients than in the tamoxifen-treated group (64, 65). This might be attributed to the widespread distribution of estrogen receptors throughout the central nervous system, where AIs-induced reductions in estrogen levels disrupt metabolic regulation and emotional states (66, 67). In a review of three landmark phase III trials for CDK4/6 inhibitors, insomnia was the most common psychiatric adverse reaction, occurring at a rate of 9.52%―16.22% (14, 15, 68). Other reported psychiatric events included confusion, psychosis, suicidal ideation, and altered mental status. The mechanism underlying psychiatric adverse reactions induced by CDK4/6 inhibitors remained incompletely understood. It is generally recognized that adult hippocampal neurogenesis participates in emotional regulation, and its abnormalities are associated with psychiatric disorders (69). CDK4/6 inhibitors inhibit the activity of the CDK4/6–Cyclin D complex, blocking Rb protein phosphorylation and thereby arresting the G1/S phase transition of the cell cycle (70). Due to the non-cell-specific nature of this action, the drug affects hippocampal neural precursor cells and tumor cells, thereby interfering with their proliferation and differentiation. Research indicated that CDK4/6 kinase activity (particularly Rb phosphorylation) was crucial for regulating the cell cycle of neural precursor cells, while the Cyclin D2–CDK6 complex played a vital role in adult neurogenesis (71). Consequently, CDK4/6 inhibitors might impair hippocampal neurogenesis by inhibiting the CDK4/6–Cyclin D complex in neural precursor cells, thereby inducing psychiatric symptoms. Notably, breast cancer patients themselves constitute a high-risk population for psychiatric disorders, exhibiting elevated rates of anxiety, depression, and stress-related disorders (72). These comorbid conditions significantly impact survival, recurrence, and quality of life. Thus, assessing and managing mental health risks is critically important in the systemic treatment of breast cancer. Given that both AIs and CDK4/6 inhibitors cause psychiatric adverse events, clinicians must closely monitor the psychological status of patients over the long term when using these drugs in combination and implement appropriate screening and intervention measures.

Descriptive analysis revealed that over 30% of ADEs took place within the initial 30 days of treatment, regardless of combination or monotherapy. The results suggested that combination therapy did not alter the early risk pattern of ADEs, but it highlighted the importance of enhanced monitoring during this initial period. In contrast, over 25% of ADEs were recorded after 360 days, indicating that combination therapy might contribute to cumulative long-term systemic toxicity. Logistic regression analysis identified age and weight as factors influencing the risk of serious outcomes. Specifically, patients aged ≤65 years and weighing ≤70 kg had a higher risk of serious outcomes, potentially reflecting greater challenges in controlling disease in these subgroups. TTO analysis further confirmed the earlier onset of ADEs in these populations. Notably, a body weight of ≥70 kg was a protective factor against hematologic toxicity. Higher body mass index and visceral adipose tissue index were found to be associated with greater improvements in progression free survival, suggesting a potential protective effect of obesity in metastatic HR+/HER2- breast cancer patients treat with combination therapy (73). Earlier research confirmed that extended hormonal stimulation and ongoing inflammation could result in worse outcomes in obese individuals. The production of estrogens by adipose tissue and the release of inflammatory mediators during adipocyte hypoxia were considered to stimulate the growth of breast cancer with estrogen receptors (74). However, for patients with advanced cancer, excess adipose tissue could serve as an energy reserve during palliative care, alleviating cancer-related cachexia. The negative impact of cachexia on survival outweighed the adverse effects of obesity itself (75). Furthermore, underweight patients faced heightened risks of sarcopenia, which had been consistently linked to poor outcomes across multiple studies (76, 77). Patients with lower body mass index often exhibited elevated plasma drug concentrations, increasing the risk of hematologic toxicities like neutropenia, a phenomenon particularly pronounced in Asian populations (78–80). Another possible explanation was related to fixed-dose drug exposure. Both the AIs and the CDK4/6 inhibitors included in this study were administered as fixed daily doses rather than weight-adjusted doses. Therefore, patients with higher body weight might receive a lower effective exposure on a mg/kg basis, potentially resulting in lower systemic exposure and fewer reported hematologic toxicity events (81, 82). However, FAERS and JADER databases did not reliably capture dose intensity, dose reductions, interruptions, adherence, or plasma drug concentrations. Consequently, we could not evaluate dose-toxicity or exposure-toxicity relationships, and the observed association between body weight and hematologic toxicity was interpreted cautiously.

It should be noted that, despite the use of large real-world datasets and data mining methods, this study had inherent limitations. Both FAERS and JADER databases were spontaneous reporting systems, lacking rigorous prospective design and standardized data entry, which might have introduced data gaps and reporting biases. Moreover, not all ADEs were reported to these databases, and factors such as concomitant medications and pre-existing medical conditions might have affected event reporting and attribution. Signal detection algorithms primarily assessed the intensity of reporting bias and statistical significance rather than establishing causality. Bonferroni correction was applied in the primary disproportionality analysis to control for multiple comparisons. Given the very large number of drug–ADEs pairs tested simultaneously, such multiplicity adjustment was necessary to limit the risk of false-positive signals. However, the Bonferroni method was inherently conservative and might have increased the risk of false negatives (type II errors) (83), especially for rare adverse events. Consequently, some true signals, particularly those with small effect sizes or very low reporting frequencies, might have been missed.

The DDI analysis was performed at the drug-class level, an approach that had been used in previous pharmacovigilance study (40). Aggregation improved statistical stability for rare adverse events and less frequently reported drug combinations. Nevertheless, individual CDK4/6 inhibitors were found to have distinct toxicity profiles. Lin et al. (84) reported that ribociclib was associated with higher risks of hepatotoxicity, nephrotoxicity, and QT prolongation; abemaciclib with higher risks of hepatotoxicity, gastrointestinal effects, interstitial lung disease, and thrombosis; and palbociclib with the highest risk of hematologic toxicity. Even among AIs, some heterogeneity existed (16). Therefore, our DDI findings were interpreted as class-level reporting interaction signals rather than definitive evidence that every specific AI-CDK4/6 inhibitor pair conferred the same risk. Future studies with larger sample sizes were needed to explore agent-specific interactions.

The FAERS database lacked detailed clinical information on important potential confounders, including comorbidities (e.g., baseline hepatic or renal function), disease stage, prior treatment lines, concomitant non-cancer medications, and exact drug dosages. In our logistic regression models, we could only adjust for the available demographic variables (age, body weight, duration of therapy, and use of concomitant chemotherapy); residual confounding by unmeasured factors could not be excluded. Furthermore, comparisons of ROR between combination therapy and monotherapy had important methodological limitations. ROR derived from spontaneous reporting databases was not directly comparable across different exposure groups, because patients receiving different regimens might have differed systematically in disease stage, prior treatment history, and other unmeasured characteristics. Higher ROR for combination therapy should therefore not be interpreted as evidence of greater clinical risk or causal effect; they merely indicated stronger disproportionality in reporting.

Finally, this study analyzed overall adverse events for the combined therapy category “AIs and CDK4/6 inhibitors” without delving into the unique safety profiles of specific drug combinations (e.g., letrozole plus palbociclib). Moreover, the pathophysiological mechanisms underlying potential pharmacokinetic or pharmacodynamic interactions between AIs and CDK4/6 inhibitors remained poorly elucidated.

## 5 Conclusion

The purpose of this study was to detect real-world safety signals by analyzing post-marketing ADEs associated with combination therapy of AIs and CDK4/6 inhibitors. Fatigue, neutropenia, white blood cell count decreased, arthralgia, decreased appetite, and anaemia were the most common ADEs. Notably, combination therapy not only showed stronger reporting signals for neutropenia, anaemia, leukopenia, alanine aminotransferase increased, and aspartate aminotransferase increased, but also associated with previously unreported new ADEs, such as skin hypopigmentation, vertigo positional, red blood cells urine positive, and plicated tongue. Therefore, clinical practices must not only remain vigilant against the known ADEs associated with AIs and CDK4/6 inhibitors individually but also be attentive to potential new ADEs linked to combination therapy. Continuous, close monitoring and proper management are crucial for preventing treatment discontinuation and maintaining patient quality of life. The signals detected from the FAERS and JADER databases required further confirmation through in-depth mechanistic studies and long-term follow-up data. In summary, this study provided a systematic pharmacovigilance analysis of combination therapy with AIs and CDK4/6 inhibitors, aiding clinicians in optimizing treatment decisions and enhancing disease management throughout the treatment journey.

## Data Availability

The original contributions presented in the study are included in the article/Supplementary material, further inquiries can be directed to the corresponding author.
