# Supplementary material for: Assessing the risk of adverse drug events from combining aromatase inhibitors with CDK4/6 inhibitors using the FAERS and JADER databases
Source: Front Med (Lausanne). 2026 Jul 6;13:1823079. doi: 10.3389/fmed.2026.1823079 (PMC13381434; doi:10.3389/fmed.2026.1823079)
Supplement: Supplementary file 1 [file Supplementary_file_1.docx]

Supplementary Material

Supplementary Figures


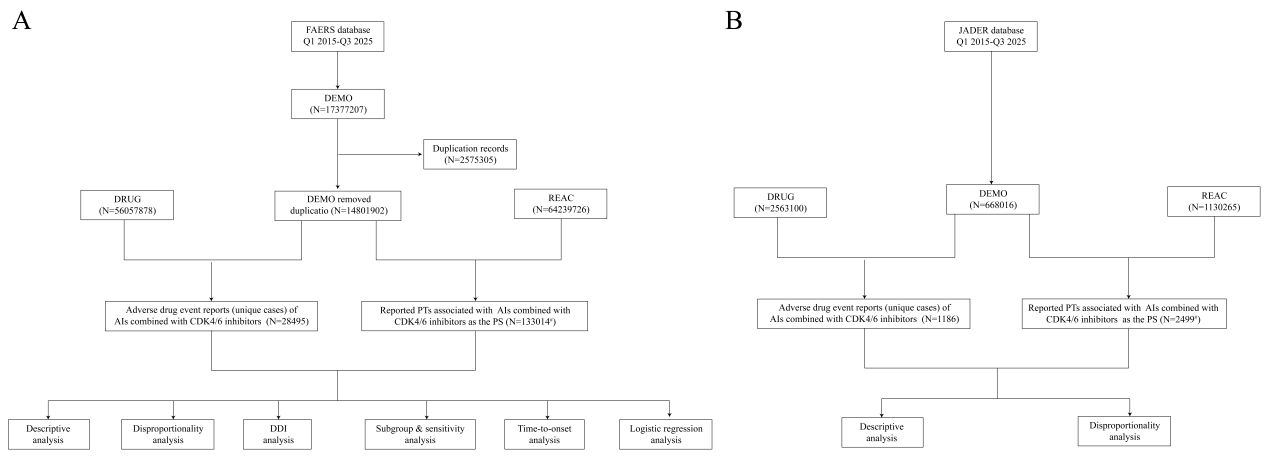


Supplementary Figure 1. Flowchart of the whole study. (A) Flowchart of data processing and analysis in the FAERS database. (B) Flowchart of data processing and analysis in the JADER database. FAERS, Food and Drug Administration Adverse Event Reporting System; DEMO, patient demographic records; DRUG, drug details; REAC, adverse events; AIs, aromatase inhibitors; CDK4/6, cyclin-dependent kinase 4/6; PTs, Preferred Terms; PS, primary suspected; DDI, Drug-Drug Interaction; JADER, Japanese Adverse Drug Event Report; #, the total number of reported PT entries.


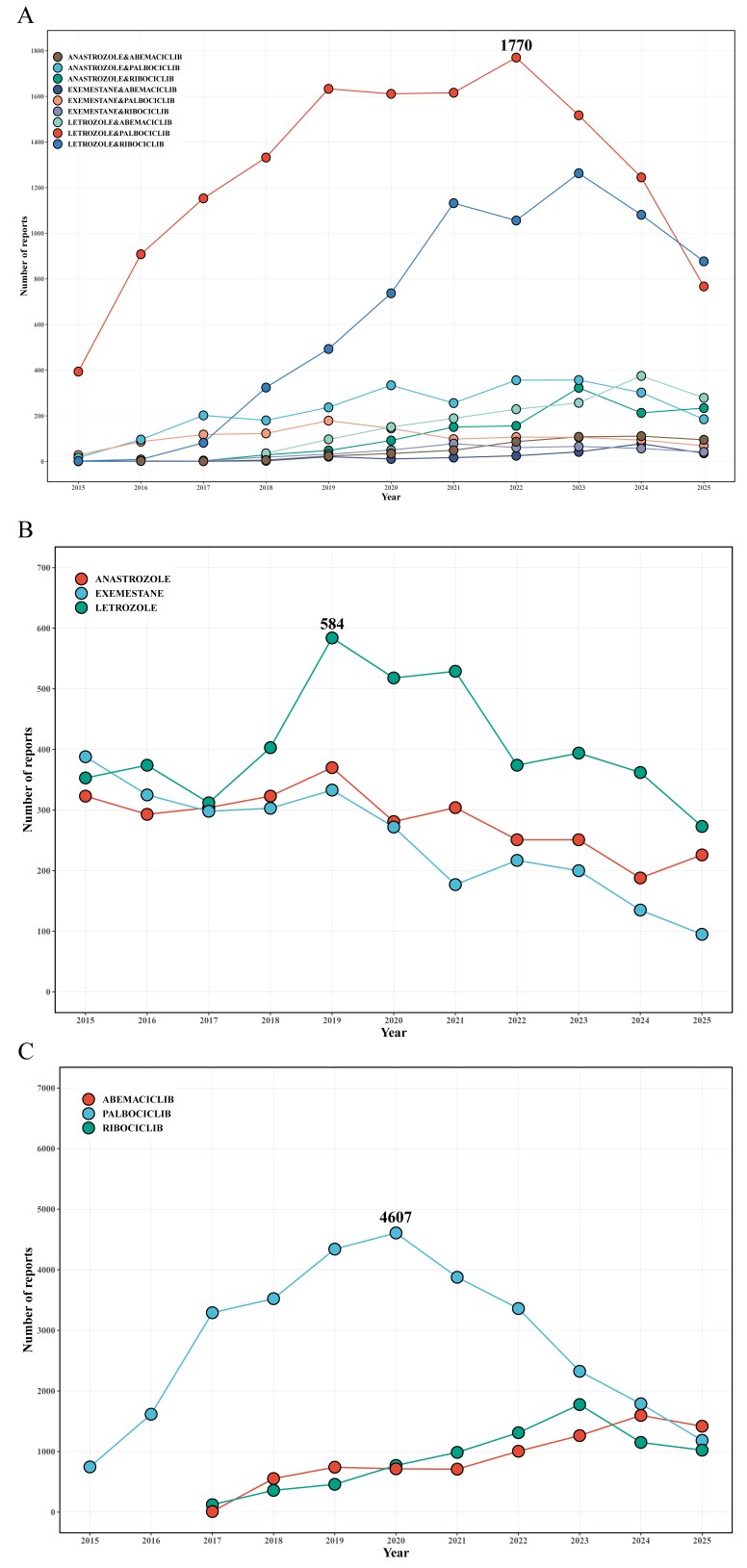


Supplementary Figure 2. Annual report counts for AIs-CDK4/6 inhibitors combinations, AIs monotherapy, and CDK4/6 inhibitors monotherapy. (A) Annual report counts for AIs and CDK4/6 inhibitors combinations. (B) Annual report counts for the three AIs. (C) Annual report counts for the four CDK4/6 inhibitors.

Supplementary Tables

Supplementary Table 1. Approval and launch dates of the included drugs.

| Drug class | Generic name | Brand name | First approval authority | First approval date |
| --- | --- | --- | --- | --- |
| CDK4/6 inhibitor | palbociclib | Ibrance | FDA (USA) | February，2015 |
| CDK4/6 inhibitor | ribociclib | Kisqali | FDA (USA) | March，2017 |
| CDK4/6 inhibitor | abemaciclib | Verzenio | FDA (USA) | September，2017 |
| CDK4/6 inhibitor | dalpiciclib | Erlikon | NMPA (China) | December，2021 |
| Aromatase inhibitor | letrozole | Femara | FDA (USA) | July，1997 |
| Aromatase inhibitor | anastrozole | Arimidex | FDA (USA) | December，1995 |
| Aromatase inhibitor | exemestane | Aromasin | FDA (USA) | October，1999 |

Abbreviations: FDA, Food and Drug Administration; NMPA, National Medical Products Administration.

Supplementary Table 2. Two-by-two contingency table for disproportionality analysis.

| Characteristic | Drug-related ADEs | Non-drug-related ADEs | Total |
| --- | --- | --- | --- |
| Drug | a | c | a+b |
| Non-drug | c | d | c+d |
| Total | a+c | b+d | N = a+b+c+d |

Abbreviations: ADEs, adverse drug events; a, number of reports containing both the target drug and target adverse drug reaction; b, number of reports containing other adverse drug reaction of the target drug; c, number of reportscontaining the target adverse drug reaction of other drugs; d: number of reports containing other drugs and other adverse drug reactions.

Supplementary Table 3. Three main algorithms for disproportionality analysis signal detection.

| Algorithms | Equation | Criteria |
| --- | --- | --- |
| ROR | ROR = ad/bc 95%CI=eln(ROR)±1.96(1/a+1/b+1/c+1/d)^0.5 | lower limit of 95%  CI>1, N≥3 |
|
| PRR | PRR = a (c+d)/c/(a+b) χ² = [(ad-bc)^2](a+b+c+d)/[(a+b)(c+d)(a+c)(b+d)] | PRR≥2, χ²≥4, N≥3 |
|
| BCPNN | IC=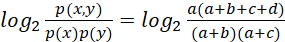  E(IC)=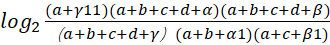  V(IC)=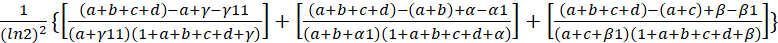  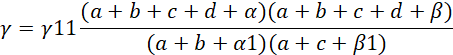  IC-2SD=E(IC)-2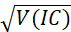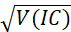  α1=β1, α=β=2 | Lower limit of IC025>0 |
|

Abbreviations: ROR, reporting odds ratio; a, number of reports containing both the target drug and target adverse drug reaction; b, number of reports containing other adverse drug reaction of the target drug; c, number of reportscontaining the target adverse drug reaction of other drugs; d, number of reports containing other drugs and other adverse drug reactions; PRR, proportional reporting ratio; χ2, chi-squared; BCPNN, bayesian confidence propagation neural network; CI, confidence interval; IC, information component; E(IC), the IC expectations; V(IC), the variance of IC; IC025, the lower limit of the 95% one-sided CI, of the IC.

Supplementary Table 4. Two-by-two contingency table for disproportionality analysis.

| Characteristic | Drug-related ADEs | Non-drug-related ADEs | Total |
| --- | --- | --- | --- |
| drugA+drugB | a | c | a+b |
| drugA/drugB | c | d | c+d |
| Total | a+c | b+d | N = a+b+c+d |

Abbreviations: ADEs, adverse drug events; drugA, aromatase inhibitors; drugB, cyclin-dependent kinase 4/6; a, number of reports containing both the target drug and target adverse drug reaction; b, number of reports containing other adverse drug reaction of the target drug; c, number of reportscontaining the target adverse drug reaction of other drugs; d: number of reports containing other drugs and other adverse drug reactions.

Supplementary Table 5. Four main algorithms for DDI signal detection.

|  | Target ADEs | Other ADEs | Total |
| --- | --- | --- | --- |
| Concomitant use of  Drug A (AIs) and Drug B (CDK4/6 inhibitors) | n_111_ | n_110_ | n_11+_ |
| Drug A (AIs) without Drug B (CDK4/6 inhibitors) | n_101_ | n_100_ | n_10+_ |
| Drug B (CDK4/6 inhibitors) without Drug A (AIs) | n_011_ | n_010_ | n_01+_ |
| Neither Drug A (AIs) or Drug B (CDK4/6 inhibitors) | n_001_ | n_000_ | n_00+_ |
| Total | n_++1_ | n_++0_ | n_+++_ |

Abbreviations: DDI, drug drug interaction; ADEs, adverse drug events; AIs: aromatase inhibitors; CDK4/6, cyclin-dependent kinase 4/6; *n*111: the number of reports.

Targeted ADEs rates for different drug exposure scenarios were as follows:

$$\text{f}_{00}\text{=}\frac{\text{n}_{001}}{\text{n}_{00\text{+}}}\text{, }{\text{ }\text{f}}_{10}\text{=}\frac{\text{n}_{101}}{\text{n}_{10\text{+}}}\text{, }{\text{ }\text{f}}_{01}\text{=}\frac{\text{n}_{011}}{\text{n}_{01\text{+}}}\text{, }{\text{ }\text{f}}_{11}\text{=}\frac{\text{n}_{111}}{\text{n}_{11\text{+}}}\text{ }$$

$$\text{g}_{11}\text{=1-}\frac{1}{\max\left( \frac{\text{f}_{00}}{\text{1-}\text{f}_{00}}\text{, }\frac{\text{f}_{10}}{\text{1-}\text{f}_{10}} \right)\text{+ }\max\left( \frac{\text{f}_{00}}{\text{1-}\text{f}_{00}}\text{, }\frac{\text{f}_{01}}{\text{1-}\text{f}_{01}} \right)\text{- }\frac{\text{f}_{00}}{\text{1-}\text{f}_{00}}\text{ +1 }}$$

$${\text{ }\text{E}}_{111}\text{=}\text{g}_{11}\text{×}\text{n}_{\text{11+}}\text{ }$$

1. Ω Shrinkage Measure Model:

$$\text{Ω=}\text{log}_{2}\frac{\text{n}_{111}\text{+0.5}}{\text{E}_{111}\text{+0.5}}\text{ }$$

$$\text{Ω}_{025}\text{=Ω-}\frac{\text{ϕ}\left( 0.975 \right)}{\log\left( 2 \right)\sqrt{\text{n}_{111}}}\text{ }\text{>}\text{ }0\text{ }$$

Where, ϕ (0.975) is 97.5% of the standard normal distribution. When Ω025 > 0, it is considered a positive signal.

1. Additive Model:

$$\frac{\text{n}_{111}}{\text{n}_{\text{11+}}}\text{-}\frac{\text{n}_{101}}{\text{n}_{\text{10+}}}\text{-}\frac{\text{n}_{011}}{\text{n}_{\text{01+}}}\text{-}\frac{\text{n}_{001}}{\text{n}_{\text{00+}}}\text{ }\text{>}\text{ }0$$

2. Multiplicative Model:

$$\frac{\text{n}_{111}\text{×}\text{n}_{001}\text{×}\text{n}_{\text{10+}}\text{×}\text{n}_{\text{01+}}}{\text{n}_{\text{11+}}\text{×}\text{n}_{\text{00+}}\text{×}\text{n}_{101}\text{×}\text{n}_{011}}\text{ }\text{>}\text{ }1$$

3. Chi-Square Statistics Model:

Supplementary Table 6. The full results of Preferred Terms for Combination Therapy in the FAERS Database.

| **Preferred Terms** | **Cases** | **ROR(95%Cl)** | **PRR(χ2)** | **IC(IC025)** | adjusted P value |
| --- | --- | --- | --- | --- | --- |
| Fatigue | 4322 | 1.37(1.32-1.42) | 1.36(328.07) | 0.36(0.31) | 1.21916E-69 |
| Neutropenia | 3205 | 2.31(2.21-2.4) | 2.27(1590.78) | 0.91(0.85) | 0 |
| White Blood Cell Count Decreased | 3059 | 2.1(2.01-2.19) | 2.07(1214.45) | 0.81(0.75) | 2.8285E-262 |
| Arthralgia | 1467 | 1.62(1.52-1.71) | 1.61(257.94) | 0.55(0.46) | 2.58725E-54 |
| Decreased Appetite | 1313 | 1.17(1.1-1.25) | 1.17(26.84) | 0.19(0.1) | 0.000973654 |
| Anaemia | 1273 | 1.38(1.3-1.47) | 1.38(105.38) | 0.38(0.29) | 4.89174E-21 |
| Malaise | 1248 | 1.32(1.24-1.41) | 1.32(76.84) | 0.32(0.23) | 8.75308E-15 |
| Dizziness | 1059 | 1.36(1.27-1.46) | 1.36(80.19) | 0.36(0.26) | 1.6383E-15 |
| Leukopenia | 1047 | 2.08(1.94-2.24) | 2.08(413.62) | 0.81(0.71) | 3.81585E-88 |
| Constipation | 968 | 1.23(1.15-1.33) | 1.23(34.38) | 0.25(0.14) | 2.05545E-05 |
| Cough | 927 | 1.19(1.11-1.28) | 1.19(22.69) | 0.21(0.1) | 0.008471979 |
| Pain In Extremity | 897 | 1.43(1.32-1.54) | 1.42(87.97) | 0.41(0.3) | 3.2787E-17 |
| Weight Decreased | 895 | 1.23(1.14-1.32) | 1.23(30.17) | 0.24(0.13) | 0.000179394 |
| Neutrophil Count Decreased | 880 | 1.84(1.71-1.99) | 1.84(246.91) | 0.69(0.58) | 7.19374E-52 |
| Back Pain | 874 | 1.4(1.3-1.51) | 1.4(77.41) | 0.39(0.28) | 6.77316E-15 |
| Haemoglobin Decreased | 862 | 2.21(2.04-2.4) | 2.2(394.13) | 0.88(0.76) | 7.00424E-84 |
| Platelet Count Decreased | 829 | 1.46(1.35-1.58) | 1.46(91.7) | 0.44(0.32) | 5.04551E-18 |
| Pruritus | 826 | 1.25(1.16-1.35) | 1.25(33.57) | 0.26(0.15) | 3.15182E-05 |
| Hot Flush | 726 | 1.8(1.65-1.96) | 1.79(187.3) | 0.66(0.54) | 7.0991E-39 |
| Thrombocytopenia | 718 | 1.25(1.16-1.36) | 1.25(29.55) | 0.27(0.15) | 0.000249994 |
| Bone Pain | 664 | 1.38(1.27-1.51) | 1.38(55.28) | 0.38(0.25) | 5.07751E-10 |
| Red Blood Cell Count Decreased | 628 | 2.4(2.18-2.64) | 2.39(344.07) | 0.96(0.82) | 5.90316E-73 |
| Insomnia | 609 | 1.42(1.29-1.55) | 1.41(57.78) | 0.4(0.27) | 1.44811E-10 |
| General Physical Health Deterioration* | 605 | 1.6(1.46-1.75) | 1.6(102.4) | 0.54(0.41) | 2.40927E-20 |
| Dry Skin | 579 | 1.59(1.45-1.74) | 1.59(95.32) | 0.53(0.4) | 8.58207E-19 |
| Nasopharyngitis | 569 | 1.93(1.75-2.13) | 1.92(182.79) | 0.74(0.6) | 7.2029E-38 |
| Decreased Immune Responsiveness* | 550 | 3.89(3.48-4.34) | 3.87(660.49) | 1.39(1.23) | 1.2513E-141 |
| Urinary Tract Infection | 537 | 1.39(1.27-1.53) | 1.39(46.31) | 0.38(0.25) | 4.91461E-08 |
| Pleural Effusion | 518 | 1.27(1.15-1.4) | 1.27(23.47) | 0.28(0.14) | 0.005854122 |
| Feeling Abnormal* | 518 | 1.74(1.58-1.93) | 1.74(121.49) | 0.63(0.49) | 1.6859E-24 |
| Full Blood Count Abnormal* | 516 | 2.5(2.24-2.77) | 2.49(307.21) | 1(0.84) | 6.51962E-65 |
| Weight Increased | 492 | 1.9(1.72-2.11) | 1.9(152.29) | 0.72(0.57) | 3.28817E-31 |
| Abdominal Discomfort* | 491 | 1.45(1.31-1.6) | 1.45(52.45) | 0.43(0.28) | 2.20873E-09 |
| Blood Creatinine Increased | 473 | 2.24(2.01-2.5) | 2.23(223.11) | 0.89(0.73) | 1.28284E-46 |
| Full Blood Count Decreased* | 454 | 2.49(2.23-2.79) | 2.49(270.23) | 1(0.83) | 7.43258E-57 |
| Memory Impairment* | 431 | 1.62(1.45-1.81) | 1.62(77.04) | 0.55(0.4) | 8.9909E-15 |
| Gait Disturbance* | 422 | 1.35(1.21-1.5) | 1.35(29.88) | 0.35(0.19) | 0.000219723 |
| Alanine Aminotransferase Increased | 405 | 1.51(1.35-1.69) | 1.51(53.74) | 0.48(0.32) | 1.17512E-09 |
| Aspartate Aminotransferase Increased | 388 | 1.53(1.37-1.72) | 1.53(54.55) | 0.49(0.33) | 7.85102E-10 |
| Electrocardiogram Qt Prolonged | 356 | 3.02(2.65-3.45) | 3.01(298.91) | 1.17(0.99) | 4.90244E-63 |
| Influenza | 317 | 2.17(1.9-2.47) | 2.16(138.26) | 0.86(0.67) | 4.22068E-28 |
| Visual Impairment* | 305 | 1.58(1.39-1.8) | 1.58(49.63) | 0.53(0.34) | 9.82485E-09 |
| Somnolence | 295 | 1.39(1.22-1.58) | 1.39(24.82) | 0.38(0.19) | 0.003067064 |
| Oropharyngeal Pain | 291 | 1.55(1.36-1.77) | 1.55(43.54) | 0.51(0.32) | 2.16852E-07 |
| Hepatotoxicity | 270 | 2.11(1.83-2.43) | 2.1(110.06) | 0.83(0.62) | 6.13638E-22 |
| Renal Impairment* | 257 | 1.42(1.23-1.63) | 1.42(24.45) | 0.4(0.2) | 0.003773694 |
| Sleep Disorder* | 239 | 2.13(1.83-2.48) | 2.13(100.32) | 0.84(0.62) | 8.45859E-20 |
| Gamma-Glutamyltransferase Increased | 228 | 2.02(1.73-2.35) | 2.02(83.23) | 0.79(0.56) | 4.57135E-16 |
| Depressed Mood* | 224 | 1.84(1.58-2.14) | 1.84(62.52) | 0.69(0.47) | 1.54673E-11 |
| Haematocrit Decreased | 210 | 2.52(2.14-2.98) | 2.52(127.9) | 1.01(0.77) | 8.78061E-26 |
| Transaminases Increased | 205 | 1.6(1.37-1.87) | 1.6(34.72) | 0.54(0.31) | 2.03886E-05 |
| Stress* | 203 | 2.19(1.86-2.59) | 2.19(91.5) | 0.87(0.63) | 7.44551E-18 |
| Lymphadenopathy* | 187 | 1.51(1.29-1.78) | 1.51(25.05) | 0.48(0.24) | 0.002884674 |
| Spinal Pain* | 186 | 2.35(1.98-2.8) | 2.35(98.13) | 0.94(0.69) | 2.76593E-19 |
| Taste Disorder | 180 | 1.54(1.3-1.82) | 1.54(26.06) | 0.5(0.25) | 0.001732277 |
| Liver Function Test Increased | 180 | 1.66(1.41-1.97) | 1.66(35.69) | 0.58(0.34) | 1.27265E-05 |
| Dyspnoea Exertional* | 178 | 1.57(1.33-1.86) | 1.57(28.14) | 0.52(0.27) | 0.000595739 |
| Cataract | 177 | 1.6(1.35-1.89) | 1.6(29.91) | 0.54(0.29) | 0.00024226 |
| Blood Alkaline Phosphatase Increased | 175 | 1.5(1.27-1.78) | 1.5(22.61) | 0.47(0.22) | 0.010233523 |
| Inflammation* | 168 | 1.86(1.55-2.22) | 1.85(48.26) | 0.7(0.44) | 2.20988E-08 |
| Eating Disorder* | 148 | 1.55(1.29-1.86) | 1.54(21.73) | 0.5(0.23) | 0.0164653 |
| Bronchitis | 147 | 1.8(1.49-2.18) | 1.8(38.61) | 0.67(0.39) | 3.00061E-06 |
| Cystitis | 142 | 1.81(1.5-2.2) | 1.81(37.97) | 0.67(0.39) | 4.17782E-06 |
| Productive Cough* | 140 | 1.76(1.45-2.13) | 1.76(33.7) | 0.64(0.36) | 3.65897E-05 |
| Sinusitis | 140 | 1.65(1.36-2) | 1.65(26.82) | 0.57(0.29) | 0.001220992 |
| Hepatic Cytolysis* | 139 | 1.57(1.29-1.9) | 1.57(21.64) | 0.52(0.24) | 0.017388821 |
| Musculoskeletal Chest Pain* | 136 | 1.66(1.37-2.02) | 1.66(27.02) | 0.58(0.3) | 0.001109297 |
| Laboratory Test Abnormal* | 133 | 1.71(1.41-2.08) | 1.71(29.28) | 0.61(0.32) | 0.000351414 |
| Mean Cell Volume Increased* | 132 | 5.02(3.94-6.39) | 5.02(211.56) | 1.59(1.25) | 8.46254E-44 |
| Breast Pain | 130 | 1.88(1.54-2.3) | 1.88(39) | 0.71(0.42) | 2.53503E-06 |
| Ageusia* | 129 | 1.65(1.35-2.02) | 1.65(24.9) | 0.57(0.28) | 0.003311714 |
| Movement Disorder* | 128 | 2.21(1.79-2.72) | 2.21(58.47) | 0.88(0.57) | 1.38561E-10 |
| Renal Disorder* | 126 | 1.9(1.55-2.34) | 1.9(38.97) | 0.72(0.42) | 2.59062E-06 |
| Cytopenia* | 125 | 2.06(1.67-2.53) | 2.05(47.9) | 0.8(0.5) | 2.86217E-08 |
| Glomerular Filtration Rate Decreased* | 123 | 3.23(2.57-4.05) | 3.22(114.56) | 1.23(0.91) | 8.99663E-23 |
| White Blood Cell Count Abnormal* | 122 | 2.38(1.92-2.96) | 2.38(66.21) | 0.95(0.64) | 2.88602E-12 |
| Red Cell Distribution Width Increased* | 121 | 2.54(2.04-3.17) | 2.54(74.97) | 1.02(0.7) | 3.58313E-14 |
| Migraine* | 120 | 1.59(1.29-1.95) | 1.59(19.88) | 0.53(0.23) | 0.044073047 |
| Nervousness* | 115 | 1.78(1.44-2.2) | 1.78(28.88) | 0.65(0.34) | 0.000443368 |
| Osteoarthritis* | 111 | 2.19(1.75-2.73) | 2.18(49.58) | 0.87(0.54) | 1.27222E-08 |
| Nasal Congestion | 109 | 2.02(1.62-2.52) | 2.02(39.83) | 0.79(0.46) | 1.74367E-06 |
| Fluid Retention* | 109 | 1.73(1.39-2.16) | 1.73(25.13) | 0.63(0.31) | 0.003033029 |
| Hepatic Steatosis* | 101 | 1.77(1.41-2.22) | 1.77(25.01) | 0.65(0.32) | 0.003281024 |
| Tooth Disorder* | 100 | 1.75(1.4-2.2) | 1.75(23.95) | 0.64(0.3) | 0.005657858 |
| Viral Infection | 100 | 2.44(1.92-3.1) | 2.44(57.1) | 0.98(0.63) | 3.02058E-10 |
| Bone Lesion* | 97 | 1.72(1.36-2.17) | 1.72(21.69) | 0.62(0.28) | 0.018095082 |
| Mean Cell Haemoglobin Increased* | 93 | 4.37(3.31-5.77) | 4.37(128.71) | 1.48(1.09) | 9.37162E-26 |
| Musculoskeletal Discomfort* | 90 | 2.58(2-3.32) | 2.58(57.19) | 1.03(0.66) | 3.01057E-10 |
| Immune System Disorder* | 89 | 2.04(1.6-2.61) | 2.04(33.58) | 0.8(0.44) | 4.35114E-05 |
| Immunodeficiency* | 88 | 1.97(1.54-2.52) | 1.96(29.91) | 0.76(0.4) | 0.000280051 |
| Chronic Kidney Disease* | 88 | 2.46(1.91-3.18) | 2.46(51.13) | 0.98(0.61) | 6.35608E-09 |
| Breast Mass* | 85 | 1.88(1.47-2.42) | 1.88(25.47) | 0.71(0.35) | 0.00269938 |
| Crying* | 80 | 2.13(1.64-2.77) | 2.13(33.61) | 0.84(0.46) | 4.43104E-05 |
| Osteopenia | 75 | 1.88(1.44-2.45) | 1.88(22.31) | 0.71(0.32) | 0.013909102 |
| Body Height Decreased* | 73 | 2.29(1.73-3.02) | 2.29(36.21) | 0.91(0.5) | 1.23012E-05 |
| Blood Urea Increased* | 71 | 1.91(1.45-2.51) | 1.91(22.29) | 0.73(0.33) | 0.014275055 |
| Pelvic Pain | 70 | 2.22(1.68-2.94) | 2.22(32.47) | 0.88(0.47) | 8.21214E-05 |
| Erysipelas* | 66 | 2.31(1.73-3.1) | 2.31(33.64) | 0.92(0.49) | 4.64765E-05 |
| Fear* | 64 | 3.22(2.35-4.41) | 3.22(59.47) | 1.23(0.77) | 1.13741E-10 |
| Head Discomfort* | 62 | 2.1(1.56-2.83) | 2.1(25.14) | 0.83(0.39) | 0.003483575 |
| Skin Hypopigmentation* | 62 | 30.89(15.84-60.23) | 30.87(248.92) | 2.36(1.76) | 2.1779E-51 |
| Blood Pressure Abnormal* | 61 | 2.15(1.6-2.91) | 2.15(26.34) | 0.85(0.41) | 0.001906191 |
| Vitiligo* | 60 | 5.75(3.96-8.33) | 5.75(109.2) | 1.68(1.16) | 2.23873E-21 |
| Increased Appetite | 54 | 2.15(1.56-2.96) | 2.15(23.23) | 0.85(0.38) | 0.009548233 |
| Platelet Count Increased* | 54 | 2.24(1.63-3.09) | 2.24(25.59) | 0.89(0.42) | 0.002895405 |
| Neutrophil Count Abnormal* | 54 | 2.32(1.68-3.2) | 2.32(27.62) | 0.93(0.45) | 0.001039756 |
| Vitamin D Deficiency | 53 | 3.43(2.42-4.86) | 3.43(53.97) | 1.29(0.77) | 1.96475E-09 |
| Eastern Cooperative Oncology Group Performance Status Worsened* | 53 | 2.51(1.81-3.5) | 2.51(32.1) | 1(0.51) | 0.000109619 |
| Blood Calcium Increased* | 51 | 2.49(1.78-3.49) | 2.49(30.31) | 1(0.5) | 0.000273859 |
| White Blood Cell Disorder* | 50 | 3.07(2.16-4.37) | 3.07(43.26) | 1.19(0.67) | 4.23435E-07 |
| Intervertebral Disc Protrusion* | 46 | 3.01(2.09-4.35) | 3.01(38.56) | 1.17(0.63) | 4.60292E-06 |
| Spinal Disorder* | 45 | 3.03(2.09-4.39) | 3.03(38.01) | 1.18(0.63) | 6.13381E-06 |
| Hunger* | 42 | 3.61(2.42-5.37) | 3.61(45.88) | 1.33(0.74) | 1.27547E-07 |
| Osteosclerosis* | 42 | 2.55(1.76-3.7) | 2.55(26.18) | 1.02(0.46) | 0.002363228 |
| Gastrointestinal Infection* | 42 | 3.61(2.42-5.37) | 3.61(45.88) | 1.33(0.74) | 1.27547E-07 |
| Renal Cyst* | 41 | 3(2.04-4.42) | 3(34.16) | 1.17(0.59) | 4.40505E-05 |
| Hiatus Hernia* | 41 | 2.58(1.77-3.77) | 2.58(26.22) | 1.03(0.47) | 0.002343722 |
| General Physical Condition Abnormal* | 41 | 2.96(2.01-4.36) | 2.96(33.35) | 1.16(0.58) | 6.586E-05 |
| Sensitive Skin* | 40 | 2.89(1.96-4.26) | 2.89(31.22) | 1.13(0.55) | 0.000193059 |
| Anosmia* | 39 | 3.74(2.47-5.66) | 3.73(44.63) | 1.36(0.74) | 2.49078E-07 |
| Neutrophil Percentage Decreased* | 38 | 15.77(8.24-30.18) | 15.77(126.15) | 2.18(1.42) | 9.68273E-25 |
| Macrocytosis* | 36 | 2.99(1.98-4.52) | 2.99(29.76) | 1.16(0.55) | 0.00042417 |
| Discouragement* | 34 | 4.7(2.94-7.52) | 4.7(50.98) | 1.54(0.85) | 1.19449E-08 |
| Anisocytosis* | 33 | 3.22(2.08-4.99) | 3.22(30.7) | 1.23(0.57) | 0.000277368 |
| Haemoglobin Increased* | 32 | 5.14(3.14-8.42) | 5.14(52.51) | 1.6(0.88) | 5.93729E-09 |
| Middle Insomnia* | 31 | 2.71(1.75-4.19) | 2.71(21.63) | 1.07(0.42) | 0.026416872 |
| Blood Creatinine Abnormal* | 31 | 2.71(1.75-4.19) | 2.71(21.63) | 1.07(0.42) | 0.026416872 |
| Hyperuricaemia* | 30 | 3.64(2.28-5.84) | 3.64(33.23) | 1.34(0.63) | 8.4071E-05 |
| Lymphocyte Percentage Increased* | 30 | 16.6(7.88-34.97) | 16.6(101.48) | 2.2(1.32) | 2.52691E-19 |
| Monocyte Percentage Increased* | 30 | 7.47(4.24-13.15) | 7.47(67.24) | 1.84(1.04) | 4.62453E-12 |
| Thrombophlebitis | 29 | 3.14(1.97-5) | 3.14(25.93) | 1.21(0.51) | 0.003202258 |
| Basophil Count Increased* | 29 | 3.7(2.29-5.99) | 3.7(32.81) | 1.35(0.63) | 0.000105597 |
| Lymphocyte Count Increased* | 29 | 4.25(2.59-6.97) | 4.25(38.86) | 1.46(0.72) | 5.3484E-06 |
| Feeling Of Despair* | 28 | 3.58(2.2-5.81) | 3.57(30.23) | 1.32(0.59) | 0.000387578 |
| Body Mass Index Increased* | 27 | 6.72(3.77-11.99) | 6.72(55.97) | 1.78(0.95) | 1.2954E-09 |
| Anaemia Macrocytic* | 25 | 4.02(2.37-6.8) | 4.02(31.34) | 1.42(0.62) | 0.000241891 |
| Reflux Gastritis* | 22 | 4.38(2.47-7.77) | 4.38(30.54) | 1.48(0.62) | 0.000394526 |
| Superficial Vein Thrombosis* | 22 | 4.06(2.31-7.12) | 4.06(27.93) | 1.42(0.57) | 0.001423477 |
| Vitamin B12 Deficiency* | 21 | 4.18(2.34-7.47) | 4.18(27.64) | 1.45(0.57) | 0.001696998 |
| Calcium Deficiency* | 21 | 6.15(3.25-11.66) | 6.15(40.53) | 1.72(0.78) | 3.16105E-06 |
| Vertigo Positional* | 20 | 19.92(7.48-53.08) | 19.92(71.87) | 2.26(1.13) | 2.6449E-08 |
| Feeling Of Body Temperature Change* | 20 | 3.56(2-6.31) | 3.56(21.44) | 1.32(0.44) | 0.036864798 |
| Prothrombin Time Prolonged* | 20 | 3.69(2.07-6.58) | 3.69(22.51) | 1.35(0.46) | 0.02174902 |
| White Blood Cells Urine Positive* | 18 | 5.27(2.72-10.23) | 5.27(30.27) | 1.62(0.62) | 0.000536524 |
| Rhinitis Allergic* | 17 | 4.23(2.22-8.08) | 4.23(22.69) | 1.46(0.47) | 0.02246154 |
| Alanine Aminotransferase Decreased* | 15 | 6.79(3.12-14.78) | 6.79(31.33) | 1.79(0.63) | 0.010805439 |
| Body Surface Area Increased* | 14 | 6.34(2.88-13.96) | 6.34(27.69) | 1.74(0.55) | 0.037655893 |
| Body Surface Area Decreased* | 13 | 9.25(3.69-23.18) | 9.25(33.47) | 1.96(0.64) | 0.007731706 |
| Plicated Tongue* | 11 | 18.26(5.09-65.45) | 18.26(38.45) | 2.23(0.66) | 0.002559855 |
| Red Blood Cells Urine Positive* | 11 | 27.39(6.07-123.57) | 27.39(43.02) | 2.34(0.71) | 0.000647554 |

Abbreviation: ROR, reporting odds ratio; CI, confidence interval; PRR, proportional reporting ratio; χ^2^, Chi-Square; IC, information component; IC025, the lower limit of the 95% CI of the IC; *, unexpected signals that were not listed in the drug label.

Supplementary Table S7. The full results of Preferred Terms for AIs Monotherapy in the FAERS Database.

| **Preferred Terms** | **Cases** | **ROR(95%Cl)** | **PRR(χ2)** | **IC(IC025)** | adjusted ***P*** value |
| --- | --- | --- | --- | --- | --- |
| Arthralgia | 1032 | 4.11(3.84-4.4) | 4.03(1962.69) | 1.81(1.71) | 0 |
| Hot Flush | 425 | 3.59(3.23-3.98) | 3.56(663.49) | 1.66(1.5) | 2.4284E-142 |
| Headache | 386 | 1.39(1.25-1.54) | 1.39(39.05) | 0.44(0.29) | 1.49439E-06 |
| Bone Pain | 363 | 2.63(2.36-2.94) | 2.62(321.46) | 1.28(1.11) | 4.41335E-68 |
| Insomnia | 341 | 2.76(2.46-3.09) | 2.75(332.89) | 1.34(1.17) | 1.50997E-70 |
| Pain In Extremity | 341 | 1.8(1.61-2.01) | 1.79(109.88) | 0.79(0.62) | 4.57083E-22 |
| Myalgia | 317 | 3.04(2.7-3.42) | 3.02(372.79) | 1.46(1.28) | 3.47704E-79 |
| Depression | 211 | 1.96(1.7-2.26) | 1.96(89.89) | 0.9(0.69) | 1.19348E-17 |
| Weight Increased | 193 | 2.37(2.04-2.75) | 2.36(135.29) | 1.15(0.92) | 1.61655E-27 |
| Gait Disturbance* | 187 | 2.03(1.75-2.36) | 2.02(88.06) | 0.95(0.72) | 3.1085E-17 |
| Hypertension | 170 | 1.61(1.38-1.89) | 1.61(36.54) | 0.65(0.41) | 6.04544E-06 |
| Osteoporosis | 137 | 7.76(6.36-9.46) | 7.74(575.03) | 2.54(2.21) | 1.6938E-122 |
| Memory Impairment* | 128 | 1.53(1.28-1.83) | 1.53(21.59) | 0.57(0.3) | 0.013162829 |
| Visual Impairment* | 125 | 2.13(1.77-2.57) | 2.13(67.63) | 1.01(0.73) | 1.00973E-12 |
| Musculoskeletal Stiffness* | 123 | 4.44(3.65-5.4) | 4.42(265.76) | 1.92(1.61) | 1.23397E-55 |
| Hyperhidrosis | 118 | 2.71(2.24-3.29) | 2.71(111.82) | 1.32(1.02) | 2.5817E-22 |
| Trigger Finger | 113 | 22.69(17.3-29.77) | 22.63(1079.34) | 3.46(3) | 5.796E-231 |
| Vision Blurred | 108 | 2.11(1.73-2.57) | 2.11(56.7) | 1(0.69) | 2.58116E-10 |
| Arthritis | 102 | 4.09(3.31-5.07) | 4.09(196.51) | 1.83(1.48) | 1.37717E-40 |
| Atrial Fibrillation* | 102 | 2.26(1.84-2.78) | 2.26(64.21) | 1.09(0.77) | 6.0757E-12 |
| Muscular Weakness* | 96 | 2.03(1.65-2.51) | 2.03(45.57) | 0.95(0.63) | 7.23812E-08 |
| Joint Stiffness* | 94 | 6.65(5.26-8.41) | 6.64(335.75) | 2.38(1.98) | 1.46462E-70 |
| Ischaemic Stroke | 88 | 6.25(4.92-7.95) | 6.24(293.14) | 2.31(1.9) | 2.46932E-61 |
| Diabetes Mellitus* | 88 | 2.21(1.77-2.76) | 2.21(52.31) | 1.06(0.72) | 2.51856E-09 |
| Cerebrovascular Accident | 85 | 1.84(1.47-2.3) | 1.84(29.74) | 0.82(0.48) | 0.000222613 |
| Musculoskeletal Pain | 85 | 2.9(2.31-3.64) | 2.89(91.63) | 1.4(1.04) | 7.4456E-18 |
| Osteopenia | 81 | 8.17(6.3-10.59) | 8.15(358.15) | 2.59(2.14) | 2.99708E-75 |
| Palpitations* | 81 | 1.71(1.36-2.15) | 1.71(21.99) | 0.72(0.38) | 0.011686336 |
| Lymphadenopathy* | 79 | 2.12(1.68-2.68) | 2.12(42.32) | 1.01(0.65) | 3.96768E-07 |
| Vertigo | 78 | 2.32(1.84-2.94) | 2.32(52.39) | 1.12(0.76) | 2.53765E-09 |
| Balance Disorder* | 76 | 2.14(1.69-2.71) | 2.14(41.59) | 1.02(0.65) | 5.80834E-07 |
| Arthropathy | 70 | 4.11(3.18-5.32) | 4.11(135.94) | 1.83(1.41) | 2.37659E-27 |
| Osteoarthritis* | 70 | 4.63(3.57-6.01) | 4.63(160.71) | 1.97(1.54) | 1.10248E-32 |
| Sleep Disorder* | 70 | 1.87(1.46-2.39) | 1.87(25.77) | 0.84(0.47) | 0.001757752 |
| Suicidal Ideation* | 70 | 8.01(6.06-10.58) | 8(303.72) | 2.57(2.08) | 2.05727E-63 |
| Cataract | 69 | 2.03(1.59-2.61) | 2.03(32.71) | 0.95(0.57) | 5.25595E-05 |
| Carpal Tunnel Syndrome | 69 | 15.43(11.25-21.17) | 15.41(518.47) | 3.18(2.6) | 1.8354E-109 |
| Blood Cholesterol Increased | 68 | 4.67(3.59-6.09) | 4.67(158.06) | 1.98(1.54) | 4.23978E-32 |
| Lymphoedema | 67 | 1.8(1.4-2.32) | 1.8(21.93) | 0.79(0.41) | 0.012565285 |
| Joint Swelling* | 67 | 2.15(1.67-2.77) | 2.15(37.19) | 1.03(0.63) | 5.56311E-06 |
| Tendonitis | 65 | 14.21(10.32-19.57) | 14.19(460.53) | 3.11(2.52) | 5.98141E-97 |
| Amnesia* | 63 | 2.71(2.08-3.53) | 2.71(59.58) | 1.32(0.9) | 7.76487E-11 |
| Cholestasis* | 63 | 3.43(2.62-4.49) | 3.43(92.14) | 1.62(1.18) | 7.17996E-18 |
| Mobility Decreased* | 62 | 2.39(1.84-3.11) | 2.39(44.64) | 1.16(0.75) | 1.36391E-07 |
| Cognitive Disorder* | 61 | 2.7(2.06-3.52) | 2.69(57.06) | 1.31(0.89) | 2.7837E-10 |
| Organising Pneumonia* | 60 | 4.97(3.74-6.59) | 4.96(151.15) | 2.05(1.57) | 1.52436E-30 |
| Bone Disorder* | 54 | 2.59(1.95-3.44) | 2.59(46.54) | 1.26(0.81) | 5.66641E-08 |
| Lower Respiratory Tract Infection* | 52 | 2.87(2.15-3.84) | 2.87(55.22) | 1.39(0.93) | 7.65112E-10 |
| Vulvovaginal Dryness | 51 | 5.87(4.29-8.03) | 5.86(158.03) | 2.24(1.69) | 6.48396E-32 |
| Irritability* | 50 | 4.14(3.05-5.61) | 4.13(97.95) | 1.84(1.33) | 5.00166E-19 |
| Night Sweats | 46 | 2.42(1.78-3.28) | 2.42(33.94) | 1.18(0.69) | 3.3291E-05 |
| Disturbance In Attention* | 38 | 2.18(1.56-3.05) | 2.18(21.77) | 1.04(0.51) | 0.016301759 |
| Rectal Haemorrhage* | 38 | 2.12(1.51-2.96) | 2.12(20.15) | 1(0.48) | 0.036783835 |
| Neuralgia* | 37 | 2.28(1.62-3.21) | 2.28(23.85) | 1.1(0.56) | 0.005774023 |
| Hypothyroidism* | 36 | 2.23(1.58-3.15) | 2.23(21.85) | 1.07(0.53) | 0.015994862 |
| Vaginal Haemorrhage | 36 | 3.45(2.42-4.91) | 3.44(53.07) | 1.62(1.03) | 2.88386E-09 |
| Tinnitus* | 35 | 2.59(1.82-3.68) | 2.59(30.03) | 1.26(0.69) | 0.000268166 |
| Body Height Decreased* | 34 | 3.34(2.32-4.8) | 3.34(47.47) | 1.58(0.97) | 4.80138E-08 |
| Rheumatoid Arthritis* | 33 | 6.23(4.21-9.22) | 6.22(109.59) | 2.31(1.59) | 2.8711E-21 |
| Bone Density Decreased | 31 | 11.82(7.56-18.47) | 11.81(190.74) | 2.95(2.06) | 2.24829E-16 |
| Crying* | 30 | 2.46(1.68-3.6) | 2.46(23.06) | 1.2(0.59) | 0.009433024 |
| Cystoid Macular Oedema* | 30 | 5.35(3.57-8.01) | 5.35(83.14) | 2.14(1.41) | 1.37242E-15 |
| Hypersensitivity Vasculitis* | 30 | 22.43(13.26-37.93) | 22.41(284.97) | 3.45(2.39) | 2.75606E-21 |
| Mood Swings* | 28 | 3.06(2.05-4.55) | 3.06(33.47) | 1.47(0.81) | 5.57821E-05 |
| Thirst* | 27 | 2.47(1.66-3.69) | 2.47(21.04) | 1.21(0.56) | 0.02722636 |
| Concomitant Disease Aggravated* | 27 | 2.82(1.88-4.22) | 2.82(27.7) | 1.37(0.71) | 0.000991719 |
| Mouth Swelling* | 26 | 4.72(3.08-7.25) | 4.72(61.34) | 2(1.23) | 6.94745E-11 |
| Dermatitis Bullous* | 26 | 4.81(3.13-7.39) | 4.81(62.9) | 2.02(1.25) | 3.24123E-11 |
| Fibromyalgia* | 25 | 7.71(4.85-12.26) | 7.71(104.5) | 2.54(1.64) | 1.52195E-09 |
| Tendon Pain* | 25 | 21.13(11.99-37.22) | 21.11(229.55) | 3.41(2.24) | 5.33415E-17 |
| Vitreous Detachment* | 25 | 15.67(9.25-26.55) | 15.67(190.02) | 3.19(2.09) | 6.53746E-15 |
| Bone Loss | 24 | 10.14(6.19-16.61) | 10.13(129.86) | 2.81(1.81) | 4.10631E-11 |
| Swollen Tongue* | 24 | 2.76(1.8-4.23) | 2.76(23.57) | 1.34(0.64) | 0.008240227 |
| Hypercholesterolaemia | 24 | 6.06(3.83-9.58) | 6.05(77.22) | 2.28(1.42) | 2.77847E-07 |
| Uveitis* | 23 | 7.1(4.4-11.44) | 7.09(88.17) | 2.45(1.52) | 5.03937E-08 |
| Osteoporotic Fracture* | 22 | 28.51(14.79-54.95) | 28.49(236.59) | 3.6(2.24) | 2.0318E-16 |
| Vaginal Discharge | 22 | 7.37(4.51-12.04) | 7.37(87.79) | 2.49(1.53) | 7.53833E-08 |
| Vitamin D Decreased | 20 | 3.57(2.21-5.74) | 3.56(31.18) | 1.66(0.83) | 0.000222205 |
| Blood Triglycerides Increased* | 20 | 3.5(2.17-5.64) | 3.5(30.27) | 1.64(0.81) | 0.000347108 |
| Mucosal Dryness* | 19 | 4.24(2.58-6.97) | 4.24(38.65) | 1.87(0.98) | 6.19492E-06 |
| Muscle Twitching* | 19 | 3.97(2.42-6.5) | 3.97(35.04) | 1.79(0.91) | 3.56613E-05 |
| Muscle Rigidity* | 18 | 20.58(10.6-39.93) | 20.57(162.77) | 3.39(1.96) | 1.57287E-11 |
| Hyperlipidaemia* | 18 | 3.61(2.18-5.96) | 3.6(28.58) | 1.68(0.79) | 0.000864831 |
| Anger* | 17 | 3.41(2.03-5.7) | 3.4(24.57) | 1.61(0.71) | 0.006390418 |
| Polyuria* | 17 | 4.53(2.67-7.67) | 4.52(37.85) | 1.95(0.98) | 0.004783934 |
| Muscle Tightness* | 17 | 3.41(2.03-5.7) | 3.4(24.57) | 1.61(0.71) | 0.006390418 |
| Ventricular Tachycardia* | 17 | 4.65(2.74-7.9) | 4.65(39.32) | 1.98(1) | 0.003446516 |
| Autoimmune Hepatitis* | 17 | 6.12(3.55-10.55) | 6.12(55.33) | 2.29(1.23) | 0.000129015 |
| Synovial Cyst* | 16 | 5.27(3.03-9.16) | 5.27(43.52) | 2.12(1.07) | 0.001741282 |
| Libido Decreased* | 16 | 5.98(3.41-10.47) | 5.98(50.71) | 2.26(1.18) | 0.000414867 |
| Tenosynovitis Stenosans | 16 | 62.19(22.78-169.76) | 62.16(229.25) | 3.96(2.1) | 5.20971E-14 |
| Dupuytren'S Contracture* | 16 | 51.82(20.28-132.44) | 51.8(217.4) | 3.89(2.08) | 1.82231E-13 |
| Acute Febrile Neutrophilic Dermatosis* | 16 | 15.55(8.06-30) | 15.54(120.93) | 3.18(1.75) | 9.23664E-09 |
| Joint Lock* | 15 | 19.43(9.5-39.75) | 19.43(131.08) | 3.35(1.78) | 5.08232E-09 |
| Grip Strength Decreased* | 15 | 11.21(5.94-21.17) | 11.21(88.43) | 2.9(1.54) | 1.24233E-06 |
| Cerebral Ischaemia* | 15 | 8.57(4.67-15.74) | 8.57(69.6) | 2.64(1.39) | 2.12409E-05 |
| Depression Suicidal* | 15 | 20.82(10.05-43.14) | 20.81(136.58) | 3.4(1.8) | 2.66302E-09 |
| Diabetes Mellitus Inadequate Control* | 15 | 4.94(2.8-8.71) | 4.94(37.57) | 2.05(0.98) | 0.00768918 |
| Optic Neuritis* | 14 | 8.24(4.41-15.4) | 8.24(62.54) | 2.61(1.31) | 9.93593E-05 |
| Joint Noise* | 14 | 24.73(11.23-54.48) | 24.72(140.23) | 3.52(1.79) | 3.60769E-09 |
| Blindness Unilateral* | 14 | 4.86(2.7-8.73) | 4.86(34.3) | 2.03(0.92) | 0.019398418 |
| Retinal Vein Occlusion* | 14 | 19.43(9.26-40.76) | 19.43(122.34) | 3.35(1.71) | 2.82304E-08 |
| Aortic Thrombosis* | 14 | 9.38(4.96-17.75) | 9.38(70.67) | 2.73(1.39) | 2.74457E-05 |
| Vitreoretinal Traction Syndrome* | 14 | 272.05(35.77-2068.95) | 271.95(251.96) | 4.25(2.04) | 1.93665E-14 |
| Purpura* | 13 | 5.61(3.03-10.41) | 5.61(38.23) | 2.19(0.99) | 0.010889896 |
| Fasciitis* | 13 | 63.15(20.59-193.69) | 63.13(187.04) | 3.97(1.87) | 5.46226E-11 |
| Tachyarrhythmia* | 13 | 5.26(2.85-9.71) | 5.26(35.3) | 2.12(0.94) | 0.019744547 |
| Superinfection* | 13 | 6.83(3.63-12.84) | 6.83(47.83) | 2.41(1.13) | 0.001769226 |
| Sjogren'S Syndrome* | 13 | 6.83(3.63-12.84) | 6.83(47.83) | 2.41(1.13) | 0.001769226 |
| Myopathy* | 12 | 5.69(2.99-10.82) | 5.69(35.85) | 2.21(0.94) | 0.02290341 |
| Eosinophilia* | 12 | 5.83(3.06-11.11) | 5.83(36.92) | 2.24(0.96) | 0.018545035 |
| Haemoperitoneum* | 12 | 116.59(26.09-520.94) | 116.55(196.39) | 4.13(1.81) | 4.6854E-11 |
| Retinal Haemorrhage* | 12 | 8.04(4.1-15.76) | 8.04(52.31) | 2.58(1.17) | 0.001173813 |
| Subarachnoid Haemorrhage* | 12 | 5.42(2.86-10.28) | 5.42(33.83) | 2.16(0.91) | 0.034381254 |
| Paresis* | 11 | 9.71(4.71-20.04) | 9.71(57.32) | 2.77(1.2) | 0.000816406 |
| Tension Headache* | 11 | 9.29(4.53-19.06) | 9.29(55.05) | 2.72(1.18) | 0.001153128 |
| Aortic Aneurysm* | 11 | 9.71(4.71-20.04) | 9.71(57.32) | 2.77(1.2) | 0.000816406 |
| Cutaneous Vasculitis* | 11 | 8.91(4.36-18.18) | 8.9(52.92) | 2.68(1.16) | 0.001606802 |
| Androgenetic Alopecia* | 11 | 42.75(14.85-123.04) | 42.74(140.11) | 3.81(1.62) | 4.00686E-08 |
| Abdominal Lymphadenopathy* | 11 | 8.91(4.36-18.18) | 8.9(52.92) | 2.68(1.16) | 0.001606802 |
| Polyarthritis* | 10 | 13.88(6.16-31.25) | 13.88(69.69) | 3.09(1.26) | 0.000244857 |
| Paraparesis* | 10 | 6.7(3.26-13.75) | 6.7(36.05) | 2.39(0.91) | 0.040343813 |
| Vestibular Disorder* | 10 | 27.76(10.56-72.93) | 27.75(106.18) | 3.59(1.44) | 3.33478E-06 |
| Breast Tenderness* | 10 | 6.94(3.37-14.29) | 6.94(37.45) | 2.43(0.93) | 0.031379009 |
| Urge Incontinence* | 10 | 14.95(6.55-34.09) | 14.94(73.53) | 3.15(1.29) | 0.000149449 |
| Low Density Lipoprotein Increased* | 10 | 8.45(4.02-17.75) | 8.45(45.75) | 2.63(1.04) | 0.007695449 |
| Anti-Neutrophil Cytoplasmic Antibody Positive Vasculitis* | 10 | 97.15(21.29-443.41) | 97.13(158.56) | 4.09(1.57) | 1.42043E-08 |
| Weight Bearing Difficulty* | 10 | 12.14(5.51-26.76) | 12.14(62.91) | 2.97(1.21) | 0.000605781 |
| Polymyositis* | 9 | 29.14(10.37-81.88) | 29.14(97.82) | 3.62(1.32) | 1.83996E-05 |
| Autoimmune Pancreatitis* | 9 | 87.43(18.89-404.68) | 87.41(139.79) | 4.06(1.42) | 2.42048E-07 |
| Abnormal Weight Gain* | 9 | 29.14(10.37-81.88) | 29.14(97.82) | 3.62(1.32) | 1.83996E-05 |
| Progesterone Increased* | 9 | 174.87(22.15-1380.31) | 174.83(155.55) | 4.2(1.44) | 4.60358E-08 |
| Sexual Dysfunction* | 9 | 8.74(3.98-19.2) | 8.74(42.55) | 2.66(0.96) | 0.019670796 |
| Ocular Hypertension* | 9 | 21.86(8.43-56.66) | 21.85(84.28) | 3.43(1.27) | 8.16918E-05 |
| Antiphospholipid Syndrome* | 9 | 58.29(15.78-215.32) | 58.28(126.66) | 3.94(1.4) | 9.25579E-07 |
| Oestrogen Receptor Assay Positive* | 9 | 9.2(4.16-20.34) | 9.2(44.64) | 2.71(0.99) | 0.014185109 |
| Ligament Pain* | 8 | 22.2(8.05-61.24) | 22.2(75.58) | 3.45(1.13) | 0.000463934 |
| Renal Infarct* | 8 | 31.09(10.17-95.03) | 31.08(89.58) | 3.65(1.18) | 0.000101391 |
| Polymyalgia Rheumatica* | 8 | 9.71(4.16-22.7) | 9.71(41.68) | 2.77(0.9) | 0.035624291 |
| Glomerulonephritis* | 8 | 10.36(4.39-24.44) | 10.36(44.12) | 2.83(0.92) | 0.024819791 |
| Blood Oestrogen Decreased* | 8 | 11.96(4.96-28.85) | 11.95(49.71) | 2.96(0.97) | 0.011250978 |
| Arrhythmia Supraventricular* | 8 | 14.13(5.68-35.13) | 14.13(56.5) | 3.1(1.03) | 0.004564969 |
| Intraocular Pressure Increased* | 8 | 11.96(4.96-28.85) | 11.95(49.71) | 2.96(0.97) | 0.011250978 |
| Pyuria* | 7 | 15.11(5.63-40.58) | 15.11(51.88) | 3.16(0.89) | 0.01552281 |
| Lichen Sclerosus* | 7 | 68(14.13-327.35) | 67.99(102.67) | 3.99(1.06) | 6.62887E-05 |
| Anhidrosis* | 7 | 68(14.13-327.35) | 67.99(102.67) | 3.99(1.06) | 6.62887E-05 |
| Antisynthetase Syndrome* | 7 | 68(14.13-327.35) | 67.99(102.67) | 3.99(1.06) | 6.62887E-05 |
| Vitreous Haemorrhage* | 7 | 12.36(4.79-31.89) | 12.36(44.67) | 2.99(0.84) | 0.039586371 |
| Myofascial Pain Syndrome* | 7 | 12.36(4.79-31.89) | 12.36(44.67) | 2.99(0.84) | 0.039586371 |
| Progesterone Receptor Assay Positive* | 7 | 13.6(5.18-35.73) | 13.6(48.06) | 3.07(0.86) | 0.025265924 |
| Prurigo* | 6 | 19.43(6.27-60.24) | 19.43(52.43) | 3.35(0.74) | 0.02935026 |
| Burnout Syndrome* | 6 | 38.86(9.72-155.37) | 38.85(73.75) | 3.77(0.81) | 0.003032889 |
| Ovarian Rupture* | 6 | 58.28(11.76-288.79) | 58.28(84.44) | 3.94(0.83) | 0.001055156 |
| Chronic Hepatitis* | 6 | 23.31(7.11-76.39) | 23.31(58.24) | 3.48(0.77) | 0.015314749 |
| Depersonalisation/Derealisation Disorder* | 6 | 38.86(9.72-155.37) | 38.85(73.75) | 3.77(0.81) | 0.003032889 |
| Gastrointestinal Hypomotility* | 6 | 23.31(7.11-76.39) | 23.31(58.24) | 3.48(0.77) | 0.015314749 |
| Diabetic Hyperosmolar Coma* | 6 | 23.31(7.11-76.39) | 23.31(58.24) | 3.48(0.77) | 0.015314749 |
| Aneurysm Ruptured* | 5 | 48.57(9.42-250.35) | 48.56(66.55) | 3.87(0.55) | 0.016205171 |
| Glomerulonephritis Rapidly Progressive* | 5 | 48.57(9.42-250.35) | 48.56(66.55) | 3.87(0.55) | 0.016205171 |
| Cerebral Microhaemorrhage* | 5 | 97.14(11.35-831.49) | 97.13(79.28) | 4.09(0.55) | 0.004826714 |
| Angina Bullosa Haemorrhagica* | 5 | 48.57(9.42-250.35) | 48.56(66.55) | 3.87(0.55) | 0.016205171 |
| Glutamate Dehydrogenase Increased* | 5 | 48.57(9.42-250.35) | 48.56(66.55) | 3.87(0.55) | 0.016205171 |

Abbreviation: ROR, reporting odds ratio; CI, confidence interval; PRR, proportional reporting ratio; χ^2^, Chi-Square; IC, information component; IC025, the lower limit of the 95% CI of the IC; *, unexpected signals that were not listed in the drug label.

Supplementary Table 8. The full results of Preferred Terms for CDK4/6 inhibitors Monotherapy in the FAERS Database.

| **Preferred Terms** | **Cases** | **ROR(95%Cl)** | **PRR(χ2)** | **IC(IC025)** | **adjusted *P* value** |
| --- | --- | --- | --- | --- | --- |
| Fatigue | 6851 | 2.35 ( 2.28 - 2.42 ) | 2.29 ( 3364.42 ) | 0.89 ( 0.85 ) | 0 |
| White Blood Cell Count Decreased | 4751 | 3.83 ( 3.68 - 3.98 ) | 3.74 ( 5242.52 ) | 1.31 ( 1.26 ) | 0 |
| Diarrhoea | 4534 | 1.53 ( 1.48 - 1.58 ) | 1.51 ( 598.93 ) | 0.47 ( 0.42 ) | 1.3903E-128 |
| Nausea | 4289 | 1.54 ( 1.48 - 1.59 ) | 1.52 ( 584.11 ) | 0.47 ( 0.42 ) | 2.3363E-125 |
| Neutropenia | 2197 | 1.22 ( 1.16 - 1.28 ) | 1.22 ( 68.08 ) | 0.23 ( 0.16 ) | 6.67699E-13 |
| Vomiting | 1946 | 1.19 ( 1.14 - 1.26 ) | 1.19 ( 47.81 ) | 0.2 ( 0.13 ) | 1.97663E-08 |
| Asthenia | 1898 | 1.37 ( 1.3 - 1.44 ) | 1.37 ( 144.68 ) | 0.36 ( 0.28 ) | 1.14831E-29 |
| Decreased Appetite | 1816 | 1.6 ( 1.51 - 1.69 ) | 1.59 ( 296.02 ) | 0.52 ( 0.44 ) | 1.21585E-62 |
| Malaise | 1584 | 1.62 ( 1.53 - 1.71 ) | 1.61 ( 271.61 ) | 0.53 ( 0.45 ) | 2.54927E-57 |
| Constipation | 1367 | 1.74 ( 1.63 - 1.85 ) | 1.73 ( 306.7 ) | 0.61 ( 0.52 ) | 6.06865E-65 |
| Headache | 1332 | 1.33 ( 1.25 - 1.42 ) | 1.33 ( 83.63 ) | 0.32 ( 0.24 ) | 2.67289E-16 |
| Dizziness | 1182 | 1.41 ( 1.32 - 1.5 ) | 1.4 ( 104.22 ) | 0.38 ( 0.29 ) | 8.38478E-21 |
| Cough | 1154 | 1.41 ( 1.32 - 1.51 ) | 1.41 ( 103.04 ) | 0.39 ( 0.29 ) | 1.52456E-20 |
| Platelet Count Decreased | 1111 | 1.94 ( 1.81 - 2.08 ) | 1.93 ( 351.2 ) | 0.72 ( 0.62 ) | 1.33068E-74 |
| Stomatitis | 1051 | 1.55 ( 1.44 - 1.66 ) | 1.54 ( 150.18 ) | 0.49 ( 0.39 ) | 7.76141E-31 |
| Weight Decreased | 999 | 1.26 ( 1.18 - 1.35 ) | 1.26 ( 41.74 ) | 0.27 ( 0.16 ) | 4.52054E-07 |
| Pruritus | 918 | 1.28 ( 1.19 - 1.38 ) | 1.28 ( 43.54 ) | 0.28 ( 0.18 ) | 1.82386E-07 |
| Full Blood Count Abnormal* | 859 | 5.57 ( 5.04 - 6.16 ) | 5.55 ( 1427.72 ) | 1.6 ( 1.47 ) | 2.6413E-308 |
| Back Pain | 843 | 1.19 ( 1.11 - 1.29 ) | 1.19 ( 20.69 ) | 0.2 ( 0.09 ) | 0.022744335 |
| Neutrophil Count Decreased | 843 | 1.55 ( 1.44 - 1.68 ) | 1.55 ( 122.44 ) | 0.49 ( 0.38 ) | 9.112E-25 |
| Haemoglobin Decreased | 815 | 1.82 ( 1.68 - 1.98 ) | 1.82 ( 213.51 ) | 0.66 ( 0.54 ) | 1.28659E-44 |
| Red Blood Cell Count Decreased | 780 | 3.01 ( 2.75 - 3.3 ) | 3 ( 623.29 ) | 1.13 ( 1.01 ) | 1.1469E-133 |
| Full Blood Count Decreased* | 744 | 5.38 ( 4.83 - 5.98 ) | 5.36 ( 1198.03 ) | 1.57 ( 1.44 ) | 2.1056E-258 |
| Abdominal Pain Upper | 714 | 1.64 ( 1.5 - 1.79 ) | 1.64 ( 129.67 ) | 0.55 ( 0.43 ) | 2.46388E-26 |
| Abdominal Discomfort* | 698 | 2.1 ( 1.92 - 2.3 ) | 2.09 ( 271.55 ) | 0.8 ( 0.67 ) | 3.1565E-57 |
| Hot Flush | 672 | 1.44 ( 1.32 - 1.58 ) | 1.44 ( 69.22 ) | 0.42 ( 0.29 ) | 4.14061E-13 |
| Insomnia | 650 | 1.38 ( 1.26 - 1.5 ) | 1.38 ( 50.98 ) | 0.36 ( 0.24 ) | 4.27132E-09 |
| Dry Skin | 640 | 1.62 ( 1.48 - 1.78 ) | 1.62 ( 111.82 ) | 0.54 ( 0.41 ) | 1.98843E-22 |
| Dehydration | 635 | 1.45 ( 1.33 - 1.59 ) | 1.45 ( 67.7 ) | 0.42 ( 0.29 ) | 9.00994E-13 |
| Peripheral Swelling* | 598 | 1.52 ( 1.38 - 1.67 ) | 1.52 ( 78.76 ) | 0.47 ( 0.34 ) | 3.41004E-15 |
| Nasopharyngitis | 579 | 1.76 ( 1.6 - 1.94 ) | 1.76 ( 137.01 ) | 0.63 ( 0.49 ) | 6.44107E-28 |
| Memory Impairment* | 553 | 2.05 ( 1.85 - 2.26 ) | 2.04 ( 202.35 ) | 0.78 ( 0.63 ) | 3.81594E-42 |
| Epistaxis | 551 | 1.5 ( 1.37 - 1.66 ) | 1.5 ( 69.32 ) | 0.46 ( 0.32 ) | 4.04922E-13 |
| Feeling Abnormal* | 550 | 1.69 ( 1.53 - 1.87 ) | 1.69 ( 112.26 ) | 0.58 ( 0.44 ) | 1.63437E-22 |
| Bone Marrow Failure* | 535 | 2.57 ( 2.31 - 2.86 ) | 2.56 ( 324.6 ) | 0.99 ( 0.85 ) | 1.01702E-68 |
| Decreased Immune Responsiveness* | 471 | 2.67 ( 2.38 - 2.99 ) | 2.67 ( 307.1 ) | 1.03 ( 0.87 ) | 6.79686E-65 |
| Gait Disturbance* | 450 | 1.31 ( 1.18 - 1.45 ) | 1.31 ( 25.24 ) | 0.31 ( 0.16 ) | 0.002256321 |
| Dyspepsia | 384 | 1.36 ( 1.21 - 1.52 ) | 1.36 ( 27.55 ) | 0.35 ( 0.18 ) | 0.000697395 |
| Somnolence | 380 | 1.74 ( 1.54 - 1.95 ) | 1.73 ( 85.09 ) | 0.61 ( 0.44 ) | 1.51164E-16 |
| Thrombosis* | 378 | 2.1 ( 1.86 - 2.38 ) | 2.1 ( 148.56 ) | 0.81 ( 0.63 ) | 2.17766E-30 |
| Infection | 363 | 1.34 ( 1.19 - 1.5 ) | 1.33 ( 23.48 ) | 0.33 ( 0.16 ) | 0.005685664 |
| Cerebrovascular Accident | 338 | 2.33 ( 2.05 - 2.66 ) | 2.33 ( 168.83 ) | 0.91 ( 0.72 ) | 8.72146E-35 |
| Dysphagia* | 331 | 1.37 ( 1.21 - 1.55 ) | 1.37 ( 25.32 ) | 0.36 ( 0.18 ) | 0.002225894 |
| Oropharyngeal Pain | 323 | 1.59 ( 1.4 - 1.81 ) | 1.59 ( 52.6 ) | 0.52 ( 0.34 ) | 2.0518E-09 |
| Hypoacusis* | 318 | 4.46 ( 3.81 - 5.21 ) | 4.45 ( 425.46 ) | 1.45 ( 1.24 ) | 1.74047E-90 |
| Influenza | 315 | 1.92 ( 1.68 - 2.19 ) | 1.91 ( 96.33 ) | 0.71 ( 0.52 ) | 5.52233E-19 |
| Confusional State* | 315 | 1.71 ( 1.5 - 1.94 ) | 1.7 ( 66.46 ) | 0.59 ( 0.41 ) | 1.87035E-12 |
| Pulmonary Oedema* | 304 | 1.97 ( 1.72 - 2.25 ) | 1.97 ( 100.17 ) | 0.74 ( 0.55 ) | 8.09434E-20 |
| Oral Pain | 301 | 1.87 ( 1.64 - 2.14 ) | 1.87 ( 85.93 ) | 0.69 ( 0.5 ) | 1.04257E-16 |
| Renal Impairment* | 293 | 1.5 ( 1.32 - 1.72 ) | 1.5 ( 36.78 ) | 0.46 ( 0.27 ) | 6.40411E-06 |
| Hepatic Enzyme Increased | 293 | 1.48 ( 1.3 - 1.69 ) | 1.48 ( 34.62 ) | 0.45 ( 0.25 ) | 1.92765E-05 |
| Electrocardiogram Qt Prolonged | 243 | 1.54 ( 1.33 - 1.79 ) | 1.54 ( 34.44 ) | 0.49 ( 0.28 ) | 2.16525E-05 |
| Taste Disorder | 242 | 2.07 ( 1.78 - 2.41 ) | 2.07 ( 91.49 ) | 0.79 ( 0.57 ) | 6.7484E-18 |
| Gastrooesophageal Reflux Disease* | 225 | 1.48 ( 1.27 - 1.72 ) | 1.48 ( 26.19 ) | 0.44 ( 0.22 ) | 0.001485067 |
| Laboratory Test Abnormal* | 221 | 3.29 ( 2.77 - 3.92 ) | 3.29 ( 202.49 ) | 1.21 ( 0.97 ) | 5.12271E-42 |
| Liver Disorder* | 219 | 1.57 ( 1.35 - 1.83 ) | 1.57 ( 33.67 ) | 0.51 ( 0.29 ) | 3.25917E-05 |
| Rhinorrhoea* | 212 | 1.49 ( 1.27 - 1.74 ) | 1.49 ( 25.23 ) | 0.45 ( 0.22 ) | 0.002451015 |
| Dysphonia* | 204 | 1.81 ( 1.53 - 2.12 ) | 1.8 ( 52.09 ) | 0.65 ( 0.42 ) | 2.90037E-09 |
| Blood Test Abnormal* | 199 | 2.93 ( 2.45 - 3.51 ) | 2.93 ( 152.77 ) | 1.11 ( 0.86 ) | 3.37702E-31 |
| Liver Function Test Increased | 195 | 1.66 ( 1.41 - 1.95 ) | 1.66 ( 36.93 ) | 0.56 ( 0.33 ) | 6.32145E-06 |
| Eating Disorder* | 185 | 1.87 ( 1.58 - 2.22 ) | 1.87 ( 52.81 ) | 0.69 ( 0.44 ) | 2.05675E-09 |
| Flatulence* | 180 | 2.13 ( 1.78 - 2.54 ) | 2.12 ( 72.53 ) | 0.82 ( 0.56 ) | 1.00665E-13 |
| Myocardial Infarction | 175 | 1.79 ( 1.5 - 2.13 ) | 1.79 ( 43.53 ) | 0.64 ( 0.39 ) | 2.27285E-07 |
| Gait Inability* | 172 | 2 ( 1.67 - 2.4 ) | 2 ( 59.63 ) | 0.76 ( 0.5 ) | 6.74514E-11 |
| Stress* | 166 | 1.48 ( 1.24 - 1.77 ) | 1.48 ( 19.6 ) | 0.45 ( 0.19 ) | 0.046030345 |
| Rash Pruritic | 164 | 1.81 ( 1.51 - 2.17 ) | 1.81 ( 42.32 ) | 0.66 ( 0.39 ) | 4.24219E-07 |
| Nervousness* | 163 | 2.65 ( 2.18 - 3.22 ) | 2.65 ( 104.76 ) | 1.02 ( 0.75 ) | 9.96794E-21 |
| Musculoskeletal Chest Pain* | 157 | 1.81 ( 1.5 - 2.18 ) | 1.81 ( 40.45 ) | 0.66 ( 0.39 ) | 1.10682E-06 |
| White Blood Cell Count Abnormal* | 154 | 3.07 ( 2.5 - 3.78 ) | 3.07 ( 127.4 ) | 1.15 ( 0.86 ) | 1.23917E-25 |
| Arthritis | 150 | 1.53 ( 1.27 - 1.84 ) | 1.53 ( 20.33 ) | 0.48 ( 0.21 ) | 0.031993286 |
| Productive Cough* | 149 | 1.71 ( 1.41 - 2.06 ) | 1.71 ( 31.65 ) | 0.6 ( 0.32 ) | 9.73742E-05 |
| Sinusitis | 149 | 1.6 ( 1.33 - 1.93 ) | 1.6 ( 24.75 ) | 0.53 ( 0.26 ) | 0.003304189 |
| Speech Disorder* | 145 | 1.82 ( 1.5 - 2.21 ) | 1.82 ( 37.86 ) | 0.66 ( 0.38 ) | 4.1893E-06 |
| Renal Disorder* | 142 | 2.01 ( 1.65 - 2.45 ) | 2.01 ( 49.8 ) | 0.76 ( 0.48 ) | 1.00656E-08 |
| Amnesia* | 138 | 1.63 ( 1.34 - 1.98 ) | 1.63 ( 24.52 ) | 0.55 ( 0.26 ) | 0.003761937 |
| Night Sweats | 135 | 2.14 ( 1.74 - 2.63 ) | 2.14 ( 55.22 ) | 0.82 ( 0.53 ) | 6.63729E-10 |
| Nasal Congestion* | 134 | 2.44 ( 1.98 - 3.02 ) | 2.44 ( 73.78 ) | 0.95 ( 0.65 ) | 5.94664E-14 |
| Immune System Disorder* | 128 | 3.2 ( 2.55 - 4.02 ) | 3.2 ( 112.61 ) | 1.19 ( 0.87 ) | 2.21029E-22 |
| Cytopenia* | 122 | 1.77 ( 1.44 - 2.19 ) | 1.77 ( 29.46 ) | 0.64 ( 0.33 ) | 0.000309752 |
| Dysstasia* | 99 | 1.71 ( 1.36 - 2.16 ) | 1.71 ( 21.26 ) | 0.6 ( 0.26 ) | 0.021316796 |
| Hypersomnia* | 96 | 2.1 ( 1.65 - 2.68 ) | 2.1 ( 37.77 ) | 0.81 ( 0.45 ) | 4.87055E-06 |
| Blood Calcium Decreased | 93 | 1.78 ( 1.4 - 2.27 ) | 1.78 ( 22.86 ) | 0.64 ( 0.29 ) | 0.009520773 |
| Emotional Disorder* | 90 | 1.81 ( 1.42 - 2.32 ) | 1.81 ( 23.27 ) | 0.66 ( 0.3 ) | 0.007753761 |
| Viral Infection | 87 | 1.78 ( 1.39 - 2.29 ) | 1.78 ( 21.39 ) | 0.64 ( 0.28 ) | 0.020436215 |
| Brain Fog* | 82 | 2.37 ( 1.81 - 3.1 ) | 2.37 ( 42.34 ) | 0.92 ( 0.53 ) | 5.117E-07 |
| Pulmonary Thrombosis* | 79 | 2.68 ( 2.03 - 3.55 ) | 2.68 ( 52.04 ) | 1.04 ( 0.63 ) | 3.99345E-09 |
| Dementia* | 78 | 2.5 ( 1.89 - 3.3 ) | 2.5 ( 44.83 ) | 0.97 ( 0.57 ) | 1.48972E-07 |
| Oral Mucosal Blistering* | 69 | 2.34 ( 1.75 - 3.14 ) | 2.34 ( 34.81 ) | 0.91 ( 0.49 ) | 2.40679E-05 |
| Blood Glucose Decreased* | 65 | 2.41 ( 1.78 - 3.26 ) | 2.41 ( 34.78 ) | 0.94 ( 0.5 ) | 2.49542E-05 |
| Blood Iron Decreased* | 61 | 2.64 ( 1.92 - 3.62 ) | 2.63 ( 38.87 ) | 1.02 ( 0.56 ) | 3.28754E-06 |
| Kidney Infection* | 60 | 2.45 ( 1.79 - 3.36 ) | 2.45 ( 33.18 ) | 0.95 ( 0.49 ) | 5.7556E-05 |
| Sinus Disorder* | 60 | 2.84 ( 2.05 - 3.93 ) | 2.84 ( 43.66 ) | 1.09 ( 0.61 ) | 3.00368E-07 |
| Increased Appetite | 59 | 2.19 ( 1.6 - 2.99 ) | 2.19 ( 25.5 ) | 0.84 ( 0.39 ) | 0.002793348 |
| Blood Disorder* | 49 | 2.3 ( 1.63 - 3.24 ) | 2.29 ( 23.62 ) | 0.89 ( 0.38 ) | 0.00769729 |
| Platelet Count Abnormal* | 46 | 2.3 ( 1.61 - 3.28 ) | 2.3 ( 22.27 ) | 0.89 ( 0.37 ) | 0.015536478 |
| Tongue Disorder* | 43 | 2.81 ( 1.92 - 4.12 ) | 2.81 ( 30.79 ) | 1.08 ( 0.52 ) | 0.000219971 |
| Sinus Congestion* | 37 | 4.12 ( 2.63 - 6.44 ) | 4.12 ( 45.33 ) | 1.39 ( 0.74 ) | 1.73233E-07 |
| Red Blood Cell Count Abnormal* | 31 | 4.06 ( 2.49 - 6.6 ) | 4.06 ( 37.35 ) | 1.38 ( 0.67 ) | 1.02049E-05 |
| Increased Tendency To Bruise* | 29 | 4.45 ( 2.66 - 7.45 ) | 4.45 ( 38.77 ) | 1.45 ( 0.7 ) | 5.30419E-06 |
| Dementia Alzheimer'S Type* | 22 | 3.92 ( 2.21 - 6.94 ) | 3.92 ( 25.4 ) | 1.35 ( 0.5 ) | 0.004688604 |
| Poikilocytosis* | 19 | 4.03 ( 2.16 - 7.49 ) | 4.03 ( 22.68 ) | 1.37 ( 0.44 ) | 0.019742119 |
| Tongue Blistering* | 19 | 4.23 ( 2.26 - 7.92 ) | 4.23 ( 24 ) | 1.41 ( 0.47 ) | 0.010342944 |
| Red Blood Cell Abnormality* | 17 | 5.82 ( 2.83 - 11.98 ) | 5.82 ( 29.39 ) | 1.63 ( 0.57 ) | 0.00082388 |
| Eastern Cooperative Oncology Group Performance Status Abnormal* | 14 | 7.79 ( 3.27 - 18.56 ) | 7.79 ( 30.11 ) | 1.79 ( 0.56 ) | 0.013452361 |

Abbreviation: ROR, reporting odds ratio; CI, confidence interval; PRR, proportional reporting ratio; χ^2^, Chi-Square; IC, information component; IC025, the lower limit of the 95% CI of the IC; *, unexpected signals that were not listed in the drug label.

Supplementary Table 9. The full results of Preferred Terms unique to the Combination Therapy in the FAERS Database.

| **Preferred Terms** | **Cases** | **ROR(95%Cl)** | **PRR(χ2)** | **IC(IC025)** | **adjusted *P* value** |
| --- | --- | --- | --- | --- | --- |
| Anaemia | 1273 | 1.38(1.3-1.47) | 1.38(105.38) | 0.38(0.29) | 4.89174E-21 |
| Leukopenia | 1047 | 2.08(1.94-2.24) | 2.08(413.62) | 0.81(0.71) | 3.81585E-88 |
| Thrombocytopenia | 718 | 1.25(1.16-1.36) | 1.25(29.55) | 0.27(0.15) | 0.000249994 |
| General Physical Health Deterioration* | 605 | 1.6(1.46-1.75) | 1.6(102.4) | 0.54(0.41) | 2.40927E-20 |
| Urinary Tract Infection | 537 | 1.39(1.27-1.53) | 1.39(46.31) | 0.38(0.25) | 4.91461E-08 |
| Pleural Effusion | 518 | 1.27(1.15-1.4) | 1.27(23.47) | 0.28(0.14) | 0.005854122 |
| Blood Creatinine Increased | 473 | 2.24(2.01-2.5) | 2.23(223.11) | 0.89(0.73) | 1.28284E-46 |
| Alanine Aminotransferase Increased | 405 | 1.51(1.35-1.69) | 1.51(53.74) | 0.48(0.32) | 1.17512E-09 |
| Aspartate Aminotransferase Increased | 388 | 1.53(1.37-1.72) | 1.53(54.55) | 0.49(0.33) | 7.85102E-10 |
| Hepatotoxicity | 270 | 2.11(1.83-2.43) | 2.1(110.06) | 0.83(0.62) | 6.13638E-22 |
| Gamma-Glutamyltransferase Increased | 228 | 2.02(1.73-2.35) | 2.02(83.23) | 0.79(0.56) | 4.57135E-16 |
| Depressed Mood* | 224 | 1.84(1.58-2.14) | 1.84(62.52) | 0.69(0.47) | 1.54673E-11 |
| Haematocrit Decreased | 210 | 2.52(2.14-2.98) | 2.52(127.9) | 1.01(0.77) | 8.78061E-26 |
| Transaminases Increased | 205 | 1.6(1.37-1.87) | 1.6(34.72) | 0.54(0.31) | 2.03886E-05 |
| Spinal Pain* | 186 | 2.35(1.98-2.8) | 2.35(98.13) | 0.94(0.69) | 2.76593E-19 |
| Dyspnoea Exertional* | 178 | 1.57(1.33-1.86) | 1.57(28.14) | 0.52(0.27) | 0.000595739 |
| Blood Alkaline Phosphatase Increased | 175 | 1.5(1.27-1.78) | 1.5(22.61) | 0.47(0.22) | 0.010233523 |
| Inflammation* | 168 | 1.86(1.55-2.22) | 1.85(48.26) | 0.7(0.44) | 2.20988E-08 |
| Bronchitis | 147 | 1.8(1.49-2.18) | 1.8(38.61) | 0.67(0.39) | 3.00061E-06 |
| Cystitis | 142 | 1.81(1.5-2.2) | 1.81(37.97) | 0.67(0.39) | 4.17782E-06 |
| Hepatic Cytolysis* | 139 | 1.57(1.29-1.9) | 1.57(21.64) | 0.52(0.24) | 0.017388821 |
| Mean Cell Volume Increased* | 132 | 5.02(3.94-6.39) | 5.02(211.56) | 1.59(1.25) | 8.46254E-44 |
| Breast Pain | 130 | 1.88(1.54-2.3) | 1.88(39) | 0.71(0.42) | 2.53503E-06 |
| Ageusia | 129 | 1.65(1.35-2.02) | 1.65(24.9) | 0.57(0.28) | 0.003311714 |
| Movement Disorder* | 128 | 2.21(1.79-2.72) | 2.21(58.47) | 0.88(0.57) | 1.38561E-10 |
| Glomerular Filtration Rate Decreased* | 123 | 3.23(2.57-4.05) | 3.22(114.56) | 1.23(0.91) | 8.99663E-23 |
| Red Cell Distribution Width Increased* | 121 | 2.54(2.04-3.17) | 2.54(74.97) | 1.02(0.7) | 3.58313E-14 |
| Migraine* | 120 | 1.59(1.29-1.95) | 1.59(19.88) | 0.53(0.23) | 0.044073047 |
| Fluid Retention* | 109 | 1.73(1.39-2.16) | 1.73(25.13) | 0.63(0.31) | 0.003033029 |
| Hepatic Steatosis* | 101 | 1.77(1.41-2.22) | 1.77(25.01) | 0.65(0.32) | 0.003281024 |
| Tooth Disorder* | 100 | 1.75(1.4-2.2) | 1.75(23.95) | 0.64(0.3) | 0.005657858 |
| Bone Lesion* | 97 | 1.72(1.36-2.17) | 1.72(21.69) | 0.62(0.28) | 0.018095082 |
| Mean Cell Haemoglobin Increased* | 93 | 4.37(3.31-5.77) | 4.37(128.71) | 1.48(1.09) | 9.37162E-26 |
| Musculoskeletal Discomfort* | 90 | 2.58(2-3.32) | 2.58(57.19) | 1.03(0.66) | 3.01057E-10 |
| Chronic Kidney Disease* | 88 | 2.46(1.91-3.18) | 2.46(51.13) | 0.98(0.61) | 0.000280051 |
| Immunodeficiency* | 88 | 1.97(1.54-2.52) | 1.96(29.91) | 0.76(0.4) | 6.35608E-09 |
| Breast Mass | 85 | 1.88(1.47-2.42) | 1.88(25.47) | 0.71(0.35) | 0.00269938 |
| Blood Urea Increased* | 71 | 1.91(1.45-2.51) | 1.91(22.29) | 0.73(0.33) | 0.014275055 |
| Pelvic Pain | 70 | 2.22(1.68-2.94) | 2.22(32.47) | 0.88(0.47) | 8.21214E-05 |
| Erysipelas* | 66 | 2.31(1.73-3.1) | 2.31(33.64) | 0.92(0.49) | 4.64765E-05 |
| Fear* | 64 | 3.22(2.35-4.41) | 3.22(59.47) | 1.23(0.77) | 1.13741E-10 |
| Skin Hypopigmentation* | 62 | 30.89(15.84-60.23) | 30.87(248.92) | 2.36(1.76) | 0.003483575 |
| Head Discomfort* | 62 | 2.1(1.56-2.83) | 2.1(25.14) | 0.83(0.39) | 2.1779E-51 |
| Blood Pressure Abnormal* | 61 | 2.15(1.6-2.91) | 2.15(26.34) | 0.85(0.41) | 0.001906191 |
| Vitiligo* | 60 | 5.75(3.96-8.33) | 5.75(109.2) | 1.68(1.16) | 2.23873E-21 |
| Neutrophil Count Abnormal | 54 | 2.32(1.68-3.2) | 2.32(27.62) | 0.93(0.45) | 0.002895405 |
| Platelet Count Increased* | 54 | 2.24(1.63-3.09) | 2.24(25.59) | 0.89(0.42) | 0.001039756 |
| Vitamin D Deficiency | 53 | 3.43(2.42-4.86) | 3.43(53.97) | 1.29(0.77) | 1.96475E-09 |
| Eastern Cooperative Oncology Group* Performance Status Worsened | 53 | 2.51(1.81-3.5) | 2.51(32.1) | 1(0.51) | 0.000109619 |
| Blood Calcium Increased | 51 | 2.49(1.78-3.49) | 2.49(30.31) | 1(0.5) | 0.000273859 |
| White Blood Cell Disorder* | 50 | 3.07(2.16-4.37) | 3.07(43.26) | 1.19(0.67) | 4.23435E-07 |
| Intervertebral Disc Protrusion* | 46 | 3.01(2.09-4.35) | 3.01(38.56) | 1.17(0.63) | 4.60292E-06 |
| Spinal Disorder* | 45 | 3.03(2.09-4.39) | 3.03(38.01) | 1.18(0.63) | 6.13381E-06 |
| Hunger* | 42 | 3.61(2.42-5.37) | 3.61(45.88) | 1.33(0.74) | 1.27547E-07 |
| Gastrointestinal Infection* | 42 | 3.61(2.42-5.37) | 3.61(45.88) | 1.33(0.74) | 0.002363228 |
| Osteosclerosis* | 42 | 2.55(1.76-3.7) | 2.55(26.18) | 1.02(0.46) | 1.27547E-07 |
| Renal Cyst* | 41 | 3(2.04-4.42) | 3(34.16) | 1.17(0.59) | 4.40505E-05 |
| General Physical Condition Abnormal* | 41 | 2.96(2.01-4.36) | 2.96(33.35) | 1.16(0.58) | 0.002343722 |
| Hiatus Hernia* | 41 | 2.58(1.77-3.77) | 2.58(26.22) | 1.03(0.47) | 6.586E-05 |
| Sensitive Skin* | 40 | 2.89(1.96-4.26) | 2.89(31.22) | 1.13(0.55) | 0.000193059 |
| Anosmia* | 39 | 3.74(2.47-5.66) | 3.73(44.63) | 1.36(0.74) | 2.49078E-07 |
| Neutrophil Percentage Decreased | 38 | 15.77(8.24-30.18) | 15.77(126.15) | 2.18(1.42) | 9.68273E-25 |
| Macrocytosis* | 36 | 2.99(1.98-4.52) | 2.99(29.76) | 1.16(0.55) | 0.00042417 |
| Discouragement* | 34 | 4.7(2.94-7.52) | 4.7(50.98) | 1.54(0.85) | 1.19449E-08 |
| Anisocytosis* | 33 | 3.22(2.08-4.99) | 3.22(30.7) | 1.23(0.57) | 0.000277368 |
| Haemoglobin Increased* | 32 | 5.14(3.14-8.42) | 5.14(52.51) | 1.6(0.88) | 5.93729E-09 |
| Middle Insomnia* | 31 | 2.71(1.75-4.19) | 2.71(21.63) | 1.07(0.42) | 0.026416872 |
| Blood Creatinine Abnormal | 31 | 2.71(1.75-4.19) | 2.71(21.63) | 1.07(0.42) | 0.026416872 |
| Lymphocyte Percentage Increased* | 30 | 16.6(7.88-34.97) | 16.6(101.48) | 2.2(1.32) | 8.4071E-05 |
| Monocyte Percentage Increased* | 30 | 7.47(4.24-13.15) | 7.47(67.24) | 1.84(1.04) | 2.52691E-19 |
| Hyperuricaemia* | 30 | 3.64(2.28-5.84) | 3.64(33.23) | 1.34(0.63) | 4.62453E-12 |
| Lymphocyte Count Increased* | 29 | 4.25(2.59-6.97) | 4.25(38.86) | 1.46(0.72) | 0.003202258 |
| Basophil Count Increased* | 29 | 3.7(2.29-5.99) | 3.7(32.81) | 1.35(0.63) | 0.000105597 |
| Thrombophlebitis | 29 | 3.14(1.97-5) | 3.14(25.93) | 1.21(0.51) | 5.3484E-06 |
| Feeling Of Despair* | 28 | 3.58(2.2-5.81) | 3.57(30.23) | 1.32(0.59) | 0.000387578 |
| Body Mass Index Increased* | 27 | 6.72(3.77-11.99) | 6.72(55.97) | 1.78(0.95) | 1.2954E-09 |
| Anaemia Macrocytic* | 25 | 4.02(2.37-6.8) | 4.02(31.34) | 1.42(0.62) | 0.000241891 |
| Reflux Gastritis* | 22 | 4.38(2.47-7.77) | 4.38(30.54) | 1.48(0.62) | 0.000394526 |
| Superficial Vein Thrombosis | 22 | 4.06(2.31-7.12) | 4.06(27.93) | 1.42(0.57) | 0.001423477 |
| Calcium Deficiency* | 21 | 6.15(3.25-11.66) | 6.15(40.53) | 1.72(0.78) | 0.001696998 |
| Vitamin B12 Deficiency* | 21 | 4.18(2.34-7.47) | 4.18(27.64) | 1.45(0.57) | 3.16105E-06 |
| Vertigo Positional* | 20 | 19.92(7.48-53.08) | 19.92(71.87) | 2.26(1.13) | 2.6449E-08 |
| Prothrombin Time Prolonged* | 20 | 3.69(2.07-6.58) | 3.69(22.51) | 1.35(0.46) | 0.036864798 |
| Feeling Of Body Temperature Change* | 20 | 3.56(2-6.31) | 3.56(21.44) | 1.32(0.44) | 0.02174902 |
| White Blood Cells Urine Positive* | 18 | 5.27(2.72-10.23) | 5.27(30.27) | 1.62(0.62) | 0.000536524 |
| Rhinitis Allergic* | 17 | 4.23(2.22-8.08) | 4.23(22.69) | 1.46(0.47) | 0.02246154 |
| Alanine Aminotransferase Decreased* | 15 | 6.79(3.12-14.78) | 6.79(31.33) | 1.79(0.63) | 0.010805439 |
| Body Surface Area Increased* | 14 | 6.34(2.88-13.96) | 6.34(27.69) | 1.74(0.55) | 0.037655893 |
| Body Surface Area Decreased* | 13 | 9.25(3.69-23.18) | 9.25(33.47) | 1.96(0.64) | 0.007731706 |
| Red Blood Cells Urine Positive* | 11 | 27.39(6.07-123.57) | 27.39(43.02) | 2.34(0.71) | 0.002559855 |
| Plicated Tongue* | 11 | 18.26(5.09-65.45) | 18.26(38.45) | 2.23(0.66) | 0.000647554 |

Abbreviation: ROR, reporting odds ratio; CI, confidence interval; PRR, proportional reporting ratio; χ^2^, Chi-Square; IC, information component; IC025, the lower limit of the 95% CI of the IC; *, unexpected signals that were not listed in the drug label.

Supplementary Table 10. Analysis of Signal Strength for Preferred Terms Comparing Combination Therapy with AIs Monotherapy in the FAERS Database.

| **Preferred Terms** | **Cases** | **ROR(95%Cl)** | **adjusted *P* value** |
| --- | --- | --- | --- |
| Fatigue | 4328 | 1.95(1.79-2.12) | 9.31529E-55 |
| Neutropenia | 3205 | 7.6(6.35-9.08) | 3.2382E-148 |
| White Blood Cell Count Decreased | 3061 | 15.24(11.8-19.69) | 1.282E-167 |
| Nausea | 2832 | 1.67(1.52-1.84) | 5.15494E-23 |
| Diarrhoea | 2262 | 1.46(1.32-1.62) | 3.22776E-10 |
| Alopecia | 1895 | 1.69(1.5-1.9) | 2.67985E-15 |
| Vomiting | 1348 | 1.53(1.34-1.75) | 1.03471E-06 |
| Decreased Appetite | 1313 | 1.8(1.56-2.08) | 1.94311E-12 |
| Anaemia | 1273 | 1.91(1.64-2.22) | 4.04018E-14 |
| Leukopenia | 1049 | 5.14(3.96-6.67) | 2.03455E-39 |
| Constipation | 969 | 1.82(1.54-2.16) | 5.50564E-09 |
| Neutrophil Count Decreased | 882 | 9.98(6.75-14.74) | 1.36823E-42 |
| Haemoglobin Decreased | 863 | 4.45(3.4-5.82) | 2.62581E-29 |
| Platelet Count Decreased | 829 | 3.29(2.59-4.17) | 9.45406E-22 |
| Thrombocytopenia | 718 | 2.13(1.72-2.62) | 1.93011E-09 |
| Red Blood Cell Count Decreased | 628 | 8.38(5.48-12.82) | 2.93722E-28 |
| General Physical Health Deterioration* | 605 | 2.61(2.03-3.35) | 2.40374E-11 |
| Dry Skin | 579 | 2.18(1.72-2.76) | 1.28138E-07 |
| Nasopharyngitis | 569 | 2.03(1.61-2.56) | 2.72643E-06 |
| Blood Creatinine Increased | 473 | 2.47(1.88-3.27) | 1.09835E-07 |
| Full Blood Count Decreased* | 454 | 13.32(7.12-24.92) | 2.02722E-22 |
| Aspartate Aminotransferase Increased | 388 | 1.89(1.44-2.49) | 0.008715655 |
| Electrocardiogram Qt Prolonged | 356 | 5.49(3.46-8.71) | 1.55147E-12 |
| Dehydration | 335 | 1.96(1.46-2.64) | 0.018258125 |
| Influenza | 317 | 3.2(2.19-4.68) | 7.97223E-07 |
| Hepatotoxicity | 270 | 7.19(3.93-13.14) | 2.447E-10 |
| Renal Impairment* | 257 | 2.79(1.87-4.14) | 0.000412453 |
| Chills* | 254 | 2.19(1.53-3.13) | 0.035119806 |
| Pancytopenia | 252 | 2.23(1.55-3.21) | 0.025727254 |
| Haematocrit Decreased | 210 | 6.15(3.26-11.6) | 5.46393E-07 |
| Transaminases Increased | 205 | 2.5(1.64-3.82) | 0.03594796 |
| Oral Pain | 193 | 2.69(1.71-4.22) | 0.024473123 |
| Liver Function Test Increased | 180 | 4.79(2.61-8.8) | 9.23802E-05 |
| Taste Disorder | 180 | 6.59(3.24-13.37) | 6.62279E-06 |
| Bone Marrow Failure* | 167 | 6.11(3.01-12.42) | 4.31335E-05 |
| Mean Cell Volume Increased* | 132 | 7.73(3.16-18.87) | 0.000431719 |
| Cytopenia* | 125 | 7.32(2.99-17.88) | 0.001204573 |
| White Blood Cell Count Abnormal* | 122 | 17.85(4.41-72.19) | 9.26436E-05 |
| Feeling Cold* | 97 | 7.09(2.61-19.29) | 0.028706325 |
| Immunodeficiency* | 89 | 13.02(3.21-52.87) | 0.013148265 |

Abbreviation: ROR, reporting odds ratio; CI, confidence interval; *, unexpected signals that were not listed in the drug label.

Supplementary Table 11. Analysis of Signal Strength for Preferred Terms Comparing Combination Therapy with CDK4/6 inhibitors Monotherapy in the FAERS Database.

| **Preferred Terms** | **Cases** | **ROR(95%Cl)** | **adjusted *P* value** |
| --- | --- | --- | --- |
| Neutropenia | 3205 | 1.61(1.53-1.7) | 3.10012E-63 |
| Arthralgia | 1469 | 1.41(1.31-1.53) | 6.07084E-15 |
| Anaemia | 1273 | 1.27(1.17-1.37) | 3.63547E-05 |
| Leukopenia | 1049 | 1.95(1.76-2.15) | 5.77211E-36 |
| Pyrexia | 917 | 1.24(1.13-1.36) | 0.030639925 |
| Thrombocytopenia | 718 | 1.8(1.6-2.03) | 2.46767E-19 |
| Bone Pain | 668 | 1.39(1.24-1.56) | 4.50322E-05 |
| General Physical Health Deterioration* | 605 | 2.32(2.02-2.68) | 5.34878E-30 |
| Pleural Effusion | 519 | 1.44(1.26-1.64) | 0.00015628 |
| Weight Increased | 492 | 1.57(1.37-1.8) | 3.73257E-07 |
| Blood Creatinine Increased | 473 | 1.54(1.34-1.77) | 3.79514E-06 |
| Alanine Aminotransferase Increased | 405 | 2.08(1.76-2.45) | 4.03728E-15 |
| Aspartate Aminotransferase Increased | 388 | 2.06(1.74-2.44) | 4.73082E-14 |
| Hypertension | 367 | 1.41(1.21-1.64) | 0.045904542 |
| Electrocardiogram Qt Prolonged | 356 | 1.61(1.37-1.89) | 3.20175E-05 |
| Oedema Peripheral | 281 | 1.77(1.47-2.14) | 7.22771E-06 |
| Hepatotoxicity | 270 | 2.19(1.78-2.7) | 8.7751E-11 |
| Interstitial Lung Disease | 247 | 1.57(1.29-1.9) | 0.018371648 |
| Sleep Disorder* | 241 | 1.77(1.45-2.18) | 9.44303E-05 |
| Blood Pressure Increased* | 240 | 1.67(1.36-2.04) | 0.001767547 |
| Gamma-Glutamyltransferase Increased | 228 | 3.91(2.96-5.16) | 1.34339E-21 |
| Osteonecrosis Of Jaw* | 217 | 2.2(1.75-2.78) | 2.4355E-08 |
| Haematocrit Decreased | 210 | 1.72(1.38-2.13) | 0.002681634 |
| Transaminases Increased | 205 | 3.36(2.55-4.42) | 3.17473E-16 |
| Spinal Pain* | 188 | 2.08(1.63-2.66) | 6.2993E-06 |
| Lymphadenopathy* | 187 | 1.99(1.57-2.53) | 4.19757E-05 |
| Discomfort* | 182 | 1.68(1.33-2.11) | 0.034172443 |
| Febrile Neutropenia | 179 | 2.21(1.71-2.84) | 1.63439E-06 |
| Blood Alkaline Phosphatase Increased | 175 | 2.4(1.84-3.13) | 9.60019E-08 |
| Inflammation* | 168 | 2.12(1.63-2.74) | 2.51713E-05 |
| Mucosal Inflammation* | 168 | 1.81(1.41-2.31) | 0.006788435 |
| Drug-Induced Liver Injury* | 148 | 2.46(1.84-3.29) | 1.43735E-06 |
| Cystitis | 142 | 2.02(1.53-2.67) | 0.001535056 |
| Hepatic Cytolysis* | 139 | 2.38(1.77-3.2) | 1.34688E-05 |
| Mean Cell Volume Increased* | 132 | 2.04(1.53-2.72) | 0.003132703 |
| Glomerular Filtration Rate Decreased* | 123 | 2.18(1.6-2.95) | 0.001302622 |
| C-Reactive Protein Increased* | 114 | 3.29(2.28-4.75) | 7.88485E-08 |
| Polyneuropathy* | 114 | 10.42(5.75-18.89) | 4.69534E-18 |
| Osteoarthritis* | 113 | 3.35(2.31-4.85) | 6.13839E-08 |
| Hyponatraemia* | 111 | 2.83(1.99-4.03) | 6.46786E-06 |
| Hepatic Steatosis* | 101 | 2.46(1.73-3.5) | 0.000897662 |
| Blood Lactate Dehydrogenase Increased* | 93 | 3.52(2.32-5.34) | 1.42219E-06 |
| Osteopenia | 75 | 2.49(1.65-3.75) | 0.026924991 |
| Lymphopenia | 73 | 4(2.44-6.56) | 1.4611E-05 |
| Cholestasis* | 69 | 6.3(3.41-11.64) | 8.68998E-08 |
| Blood Cholesterol Increased | 68 | 3.39(2.1-5.48) | 0.000610535 |
| Hepatocellular Injury* | 68 | 2.57(1.66-3.97) | 0.04497232 |
| Erysipelas* | 66 | 3.45(2.11-5.63) | 0.00073029 |
| Skin Hypopigmentation* | 62 | 22.66(7.11-72.19) | 1.03386E-10 |
| Hypertensive Crisis* | 58 | 10.6(4.57-24.56) | 4.18247E-08 |
| Eastern Cooperative Oncology Group Performance Status Worsened* | 53 | 6.46(3.18-13.09) | 1.57467E-05 |
| Vitamin D Deficiency | 53 | 6.46(3.18-13.09) | 1.57467E-05 |
| Hyperthyroidism* | 49 | 4.13(2.24-7.62) | 0.004220153 |
| Intervertebral Disc Protrusion* | 46 | 3.88(2.1-7.18) | 0.017144809 |
| Osteosclerosis* | 42 | 4.6(2.31-9.18) | 0.010064755 |
| General Physical Condition Abnormal* | 41 | 4.99(2.43-10.28) | 0.006856339 |
| Hepatic Cyst* | 38 | 6.94(2.93-16.42) | 0.00176233 |
| Neutrophil Percentage Decreased | 38 | 5.95(2.66-13.33) | 0.004814671 |
| Acute Hepatic Failure* | 37 | 8.11(3.19-20.64) | 0.001028931 |
| Ischaemic Stroke* | 37 | 8.11(3.19-20.64) | 0.001028931 |
| Hyperuricaemia* | 30 | 8.22(2.9-23.34) | 0.014414164 |
| Peripheral Sensory Neuropathy* | 29 | 7.95(2.79-22.61) | 0.024614196 |
| Thrombophlebitis | 29 | 7.95(2.79-22.61) | 0.024614196 |

Abbreviation: ROR, reporting odds ratio; CI, confidence interval; *, unexpected signals that were not listed in the drug label.

Supplementary Table 12. The full results of Preferred Terms for DDI signals Between AIs and CDK4/6 inhibitors in the FAERS Database.

| Preferred Terms | **n111** | **Ω shrinkage model** |  | **Additive model** |  | **Multiplicative model** |  | **Chi-squared model (χ)** |  |
| --- | --- | --- | --- | --- | --- | --- | --- | --- | --- |
| Neutropenia | 3205 | 0.63 | P | 0.01564 | P | 11.40 | P | 26.87 | P |
| Anaemia | 1273 | 0.26 | P | 0.00386 | P | 2.09 | P | 8.37 | P |
| Leukopenia | 1047 | 0.87 | P | 0.00616 | P | 5.33 | P | 21.77 | P |
| Neutrophil Count Decreased | 880 | 0.10 | P | 0.00334 | P | 18.05 | P | 4.01 | P |
| Haemoglobin Decreased | 862 | 0.12 | P | 0.00169 | P | 10.97 | P | 4.36 | P |
| Thrombocytopenia | 718 | 0.05 | P | 0.00471 | P | 1.31 | P | 2.84 | P |
| General Physical Health Deterioration* | 605 | 0.39 | P | 0.00405 | P | 1.59 | P | 8.58 | P |
| Decreased Immune Responsiveness* | 550 | 0.24 | P | 0.00121 | P | 154.74 | P | 5.81 | P |
| Pleural Effusion | 518 | 0.12 | P | 0.00106 | P | 1.02 | P | 3.84 | P |
| Blood Creatinine Increased | 473 | 0.46 | P | 0.00119 | P | 4.12 | P | 8.93 | P |
| Alanine Aminotransferase Increased | 405 | 0.33 | P | 0.00195 | P | 1.11 | P | 6.55 | P |
| Aspartate Aminotransferase Increased | 388 | 0.34 | P | 0.00204 | P | 1.29 | P | 6.60 | P |
| Electrocardiogram Qt Prolonged | 356 | 0.53 | P | 0.00121 | P | 13.37 | P | 9.01 | P |
| Hepatotoxicity | 270 | 0.80 | P | 0.00186 | P | 6.44 | P | 11.28 | P |
| Haematocrit Decreased | 210 | 0.58 | P | 0.00097 | P | 9.94 | P | 7.91 | P |
| Stress* | 203 | 0.10 | P | 0.00028 | P | 4.61 | P | 2.91 | P |
| Spinal Pain* | 186 | 0.44 | P | 0.00050 | P | 2.22 | P | 6.11 | P |
| Dyspnoea Exertional* | 178 | 0.23 | P | 0.00036 | P | 1.46 | P | 4.10 | P |
| Inflammation* | 168 | 0.04 | P | 0.00029 | P | 1.06 | P | 2.32 | P |
| Cystitis | 142 | 0.55 | P | 0.00070 | P | 1.97 | P | 6.54 | P |
| Mean Cell Volume Increased* | 132 | 0.74 | P | 0.00049 | P | 32.63 | P | 7.98 | P |
| Glomerular Filtration Rate Decreased* | 123 | 0.86 | P | 0.00052 | P | 6.82 | P | 8.78 | P |
| Red Cell Distribution Width Increased* | 121 | 0.52 | P | 0.00041 | P | 5.44 | P | 6.01 | P |
| Fluid Retention* | 109 | 0.15 | P | 0.00023 | P | 2.79 | P | 3.04 | P |
| Viral Infection | 100 | 0.05 | P | 0.00018 | P | 9.55 | P | 2.27 | P |
| Mean Cell Haemoglobin Increased* | 93 | 0.26 | P | 0.00022 | P | 10.53 | P | 3.68 | P |
| Immunodeficiency* | 88 | 0.02 | P | 0.00038 | P | 22.53 | P | 2.07 | P |
| Chronic Kidney Disease* | 88 | 0.59 | P | 0.00031 | P | 2.53 | P | 5.89 | P |
| Pelvic Pain | 70 | 0.10 | P | 0.00014 | P | 3.35 | P | 2.51 | P |
| Erysipelas* | 66 | 0.71 | P | 0.00038 | P | 1.47 | P | 6.06 | P |
| Skin Hypopigmentation* | 62 | 3.29 | P | 0.00043 | P | 29.68 | P | 26.95 | P |
| Blood Pressure Abnormal* | 61 | 0.21 | P | 0.00016 | P | 4.22 | P | 3.04 | P |
| Vitiligo* | 60 | 1.07 | P | 0.00032 | P | 51.07 | P | 8.05 | P |
| Neutrophil Count Abnormal | 54 | 0.14 | P | 0.00012 | P | 4.92 | P | 2.62 | P |
| Blood Calcium Increased | 51 | 0.27 | P | 0.00017 | P | 6.78 | P | 3.29 | P |
| Osteosclerosis* | 42 | 0.69 | P | 0.00029 | P | 1.48 | P | 5.18 | P |
| Renal Cyst* | 41 | 0.13 | P | 0.00012 | P | 1.23 | P | 2.52 | P |
| General Physical Condition Abnormal* | 41 | 0.92 | P | 0.00026 | P | 1.58 | P | 6.30 | P |
| Sensitive Skin* | 40 | 0.27 | P | 0.00012 | P | 5.61 | P | 3.13 | P |
| Anosmia* | 39 | 0.47 | P | 0.00014 | P | 8.58 | P | 4.01 | P |
| Discouragement* | 34 | 0.44 | P | 0.00012 | P | 8.00 | P | 3.75 | P |
| Haemoglobin Increased* | 32 | 0.75 | P | 0.00014 | P | 4.84 | P | 5.06 | P |
| Hyperuricaemia* | 30 | 0.99 | P | 0.00019 | P | 1.13 | P | 6.01 | P |
| Monocyte Percentage Increased* | 30 | 1.62 | P | 0.00018 | P | 16.76 | P | 9.07 | P |
| Body Mass Index Increased* | 27 | 0.07 | P | 0.00007 | P | 12.31 | P | 2.18 | P |
| Reflux Gastritis* | 22 | 0.06 | P | 0.00007 | P | 1.24 | P | 2.12 | P |
| Vitamin B12 Deficiency* | 21 | 0.85 | P | 0.00011 | P | 10.05 | P | 4.89 | P |
| Calcium Deficiency* | 21 | 1.77 | P | 0.00014 | P | 4.64 | P | 8.87 | P |
| Prothrombin Time Prolonged* | 20 | 0.88 | P | 0.00016 | P | 1.60 | P | 4.96 | P |
| White Blood Cells Urine Positive* | 18 | 0.18 | P | 0.00006 | P | 5.75 | P | 2.46 | P |
| Rhinitis Allergic* | 17 | 0.65 | P | 0.00008 | P | 9.50 | P | 3.96 | P |

Abbreviations: DDI, drug drug interaction; AIs: aromatase inhibitors; CDK4/6, cyclin-dependent kinase 4/6; *n*111, the number of reports. P, positive signal; *, unexpected signals that were not listed in the drug label.

Supplementary Table 13. The full results of Preferred Terms for Combination Therapy in patients aged ≥65 Years Old: A FAERS Database Study.

| **Preferred Terms** | **Cases** | **ROR(95%Cl)** | **PRR(χ2)** | **IC(IC025)** | **adjusted *P* value** |
| --- | --- | --- | --- | --- | --- |
| Fatigue | 1752 | 1.34(1.27-1.42) | 1.33(106.64) | 0.31(0.23) | 1.73337E-21 |
| White Blood Cell Count Decreased | 1424 | 1.85(1.73-1.97) | 1.83(355.69) | 0.63(0.54) | 9.65965E-76 |
| Neutropenia | 1132 | 2.03(1.88-2.18) | 2(367.04) | 0.71(0.61) | 3.47553E-78 |
| Anaemia | 533 | 1.33(1.2-1.47) | 1.33(31.58) | 0.31(0.17) | 6.20382E-05 |
| Arthralgia | 443 | 1.34(1.2-1.49) | 1.33(27.13) | 0.31(0.16) | 0.00061976 |
| Haemoglobin Decreased | 377 | 1.76(1.56-1.99) | 1.76(82.13) | 0.59(0.41) | 4.89805E-16 |
| Platelet Count Decreased | 376 | 1.55(1.38-1.75) | 1.55(51.03) | 0.47(0.29) | 3.23097E-09 |
| Neutrophil Count Decreased | 332 | 1.84(1.61-2.1) | 1.83(83.08) | 0.63(0.44) | 3.12308E-16 |
| Red Blood Cell Count Decreased | 316 | 2.07(1.8-2.37) | 2.06(108.71) | 0.74(0.54) | 8.03374E-22 |
| Leukopenia | 281 | 1.59(1.38-1.84) | 1.59(42.54) | 0.49(0.29) | 2.50504E-07 |
| Full Blood Count Decreased* | 262 | 1.99(1.71-2.32) | 1.98(81.85) | 0.7(0.49) | 6.13187E-16 |
| Nasopharyngitis | 243 | 1.68(1.44-1.95) | 1.67(44.7) | 0.54(0.32) | 8.59693E-08 |
| Full Blood Count Abnormal* | 241 | 1.66(1.43-1.94) | 1.66(43) | 0.53(0.32) | 2.03379E-07 |
| Blood Creatinine Increased | 237 | 2.03(1.73-2.38) | 2.02(77.83) | 0.72(0.5) | 4.74394E-15 |
| Feeling Abnormal* | 230 | 1.53(1.31-1.78) | 1.52(28.96) | 0.45(0.23) | 0.000261322 |
| Weight Increased | 177 | 1.72(1.43-2.06) | 1.72(35.51) | 0.57(0.31) | 9.63863E-06 |
| Decreased Immune Responsiveness* | 171 | 2.45(2.01-2.98) | 2.44(85.89) | 0.89(0.61) | 9.14399E-17 |
| Electrocardiogram Qt Prolonged | 131 | 2.44(1.95-3.05) | 2.44(65.56) | 0.89(0.57) | 2.71385E-12 |
| Cataract | 111 | 1.74(1.39-2.19) | 1.74(23.43) | 0.58(0.25) | 0.004995823 |
| Influenza | 107 | 1.7(1.35-2.15) | 1.7(20.85) | 0.56(0.23) | 0.018843746 |
| Haematocrit Decreased | 96 | 2.45(1.89-3.19) | 2.45(48.56) | 0.89(0.52) | 1.54967E-08 |
| Bronchitis | 82 | 1.85(1.42-2.42) | 1.85(21) | 0.64(0.25) | 0.018467747 |
| Renal Disorder | 77 | 2.09(1.58-2.77) | 2.09(27.37) | 0.75(0.35) | 0.000730091 |
| Stress* | 75 | 2.17(1.63-2.9) | 2.17(29.18) | 0.78(0.37) | 0.000294332 |
| Cytopenia* | 72 | 2.97(2.17-4.06) | 2.96(50.74) | 1.04(0.61) | 5.82727E-09 |
| Mean Cell Volume Increased* | 72 | 4.2(2.98-5.92) | 4.2(79.78) | 1.29(0.84) | 2.96942E-15 |
| Glomerular Filtration Rate Decreased* | 67 | 3.3(2.37-4.61) | 3.3(55.33) | 1.13(0.66) | 6.08198E-10 |
| Red Cell Distribution Width Increased* | 56 | 2.55(1.8-3.59) | 2.54(30.41) | 0.92(0.43) | 0.000174499 |
| Chronic Kidney Disease* | 54 | 2.33(1.65-3.29) | 2.33(24.67) | 0.85(0.36) | 0.003190997 |
| Nasal Congestion* | 52 | 2.19(1.55-3.1) | 2.19(20.73) | 0.79(0.3) | 0.023828285 |
| Mean Cell Haemoglobin Increased* | 47 | 3.66(2.43-5.5) | 3.65(44.33) | 1.2(0.63) | 1.78715E-07 |
| Blood Pressure Abnormal* | 40 | 2.64(1.75-3.98) | 2.64(23.24) | 0.95(0.36) | 0.007343832 |
| Blood Calcium Increased | 38 | 2.71(1.78-4.15) | 2.71(23.16) | 0.97(0.37) | 0.007811877 |
| Intervertebral Disc Protrusion* | 29 | 4.83(2.76-8.48) | 4.83(37.01) | 1.38(0.62) | 9.25918E-06 |
| Skin Hypopigmentation* | 27 | 94.51(12.84-695.53) | 94.46(89.18) | 2.12(1.15) | 8.53158E-17 |
| Vitiligo* | 22 | 5.5(2.81-10.75) | 5.5(31.48) | 1.46(0.55) | 0.000173839 |
| Fear* | 22 | 3.85(2.1-7.05) | 3.85(22.09) | 1.24(0.38) | 0.017584629 |
| Neutrophil Percentage Decreased | 17 | 19.83(5.81-67.67) | 19.82(45.59) | 1.93(0.74) | 1.22913E-05 |
| Monocyte Percentage Increased* | 17 | 9.92(3.91-25.15) | 9.91(35.54) | 1.73(0.61) | 3.11356E-05 |
| Bicytopenia* | 16 | 5.6(2.54-12.34) | 5.6(23.24) | 1.47(0.39) | 0.012570586 |
| Lymphocyte Percentage Increased* | 14 | 9.8(3.53-27.2) | 9.8(29.1) | 1.73(0.48) | 0.007259047 |
| Prothrombin Time Prolonged* | 14 | 8.17(3.14-21.25) | 8.16(26.4) | 1.65(0.43) | 0.019208486 |
| Acute Cutaneous Lupus Erythematosus* | 12 | 21(4.7-93.81) | 20.99(32.64) | 1.95(0.49) | 0.002316864 |
| Vertigo Positional* | 10 | 34.99(4.48-273.36) | 34.98(30.01) | 2.03(0.38) | 0.007157437 |

Abbreviation: ROR, reporting odds ratio; CI, confidence interval; PRR, proportional reporting ratio; χ^2^, Chi-Square; IC, information component; IC025, the lower limit of the 95% CI of the IC; *, unexpected signals that were not listed in the drug label.

Supplementary Table 14. The full results of Preferred Terms for Combination Therapy in patients aged ＜65 Years Old: A FAERS Database Study.

| **Preferred Terms** | **Cases** | **ROR(95%Cl)** | **PRR(χ2)** | **IC(IC025)** | **adjusted *P* value** |
| --- | --- | --- | --- | --- | --- |
| Fatigue | 1917 | 1.56(1.48-1.64) | 1.54(289.28) | 0.51(0.43) | 2.71208E-61 |
| White Blood Cell Count Decreased | 1447 | 2.13(2-2.26) | 2.1(610.41) | 0.84(0.76) | 4.3188E-131 |
| Neutropenia | 1254 | 2.13(2-2.28) | 2.11(533.87) | 0.85(0.75) | 1.9302E-114 |
| Arthralgia | 738 | 1.87(1.72-2.03) | 1.86(219.73) | 0.71(0.59) | 4.46941E-46 |
| Headache | 610 | 1.36(1.24-1.48) | 1.35(45.36) | 0.36(0.23) | 5.72645E-08 |
| Malaise | 569 | 1.62(1.48-1.78) | 1.62(104.25) | 0.56(0.43) | 6.97713E-21 |
| Neutrophil Count Decreased | 456 | 1.79(1.61-1.99) | 1.78(118.37) | 0.67(0.51) | 6.04381E-24 |
| Back Pain | 421 | 1.54(1.38-1.71) | 1.53(61.24) | 0.5(0.34) | 1.91714E-11 |
| Dizziness | 418 | 1.36(1.22-1.51) | 1.35(31.21) | 0.36(0.2) | 8.12403E-05 |
| Pain In Extremity | 416 | 1.62(1.45-1.8) | 1.61(75.22) | 0.56(0.4) | 1.65236E-14 |
| Haemoglobin Decreased | 413 | 2.6(2.31-2.92) | 2.59(274.06) | 1.06(0.89) | 8.28461E-58 |
| Constipation | 399 | 1.41(1.26-1.57) | 1.41(37.57) | 0.4(0.24) | 3.16856E-06 |
| Hot Flush | 387 | 1.87(1.66-2.1) | 1.86(115.66) | 0.72(0.55) | 2.4423E-23 |
| Platelet Count Decreased | 378 | 1.42(1.27-1.59) | 1.41(36.68) | 0.41(0.25) | 5.00123E-06 |
| Pruritus | 356 | 1.39(1.24-1.56) | 1.39(30.82) | 0.39(0.22) | 0.000100698 |
| Bone Pain | 342 | 1.66(1.47-1.88) | 1.66(69.07) | 0.59(0.41) | 3.78491E-13 |
| Weight Decreased | 342 | 1.41(1.25-1.59) | 1.41(32.63) | 0.41(0.23) | 4.00774E-05 |
| Insomnia | 337 | 1.98(1.75-2.24) | 1.97(119.71) | 0.78(0.6) | 3.30647E-24 |
| Red Blood Cell Count Decreased | 295 | 2.84(2.47-3.27) | 2.83(231.67) | 1.14(0.94) | 1.55528E-48 |
| Decreased Immune Responsiveness* | 284 | 4.54(3.88-5.32) | 4.52(428.44) | 1.55(1.33) | 3.28531E-91 |
| Dry Skin | 279 | 1.91(1.67-2.19) | 1.91(89.35) | 0.74(0.54) | 1.45752E-17 |
| Weight Increased | 265 | 2.49(2.15-2.88) | 2.48(161.85) | 1.01(0.8) | 2.41003E-33 |
| Full Blood Count Abnormal* | 259 | 3.42(2.93-3.99) | 3.41(272.26) | 1.31(1.09) | 2.59211E-57 |
| Abdominal Discomfort | 241 | 1.87(1.61-2.16) | 1.87(72.47) | 0.72(0.51) | 7.35624E-14 |
| Nasopharyngitis | 231 | 1.73(1.49-2) | 1.73(53.73) | 0.63(0.42) | 9.42039E-10 |
| Feeling Abnormal* | 226 | 2.44(2.09-2.86) | 2.44(132.8) | 1(0.77) | 5.32201E-27 |
| Gait Disturbance* | 194 | 1.84(1.56-2.17) | 1.84(55.72) | 0.7(0.47) | 3.59842E-10 |
| Memory Impairment* | 191 | 1.98(1.68-2.34) | 1.98(68.33) | 0.78(0.54) | 6.28274E-13 |
| Full Blood Count Decreased* | 184 | 2.93(2.45-3.5) | 2.92(152.19) | 1.17(0.91) | 3.57014E-31 |
| Dyspepsia | 177 | 1.54(1.3-1.82) | 1.54(26.19) | 0.51(0.26) | 0.001177057 |
| Influenza | 158 | 2.46(2.04-2.96) | 2.45(94.18) | 1(0.73) | 1.54455E-18 |
| Oropharyngeal Pain | 152 | 1.62(1.35-1.94) | 1.62(27.85) | 0.56(0.3) | 0.000516544 |
| Somnolence | 144 | 1.72(1.42-2.07) | 1.71(32.65) | 0.63(0.35) | 4.51515E-05 |
| Hepatotoxicity | 128 | 2.16(1.76-2.65) | 2.16(57.2) | 0.87(0.57) | 1.91027E-10 |
| Blood Creatinine Increased | 120 | 2.06(1.67-2.54) | 2.05(47.35) | 0.82(0.51) | 2.7687E-08 |
| Electrocardiogram Qt Prolonged | 117 | 3.5(2.78-4.41) | 3.5(127.5) | 1.34(1) | 1.05035E-25 |
| Depressed Mood* | 107 | 1.87(1.5-2.33) | 1.87(32.2) | 0.72(0.4) | 6.06026E-05 |
| Thrombosis | 102 | 1.67(1.34-2.08) | 1.67(20.99) | 0.6(0.27) | 0.018636521 |
| Discomfort* | 99 | 1.83(1.46-2.29) | 1.83(27.8) | 0.7(0.36) | 0.000577526 |
| Stress* | 98 | 2.34(1.85-2.97) | 2.34(52.9) | 0.96(0.61) | 1.82175E-09 |
| Lymphadenopathy* | 95 | 1.71(1.36-2.15) | 1.71(21.23) | 0.62(0.28) | 0.01672958 |
| Haematocrit Decreased | 94 | 2.78(2.17-3.56) | 2.78(71.11) | 1.13(0.76) | 2.01723E-13 |
| Spinal Pain* | 93 | 2.36(1.85-3.01) | 2.36(50.84) | 0.96(0.6) | 5.22737E-09 |
| Inflammation* | 86 | 2.31(1.79-2.97) | 2.31(44.84) | 0.94(0.57) | 1.09278E-07 |
| Taste Disorder | 81 | 1.83(1.42-2.35) | 1.82(22.69) | 0.7(0.32) | 0.00819592 |
| Migraine* | 75 | 1.88(1.44-2.44) | 1.87(22.81) | 0.72(0.34) | 0.00783474 |
| Productive Cough* | 73 | 2.07(1.58-2.71) | 2.07(29.29) | 0.83(0.43) | 0.000293747 |
| Breast Pain | 72 | 2.13(1.62-2.8) | 2.13(31.05) | 0.86(0.45) | 0.000121256 |
| Eating Disorder* | 69 | 2.05(1.56-2.71) | 2.05(27.05) | 0.82(0.41) | 0.000928701 |
| Hepatitis | 66 | 1.98(1.5-2.63) | 1.98(23.63) | 0.78(0.37) | 0.005337638 |
| Sinusitis | 64 | 1.98(1.49-2.63) | 1.98(22.72) | 0.78(0.36) | 0.008556244 |
| Movement Disorder* | 63 | 2.73(2.02-3.69) | 2.73(46.07) | 1.11(0.66) | 6.71487E-08 |
| Laboratory Test Abnormal | 60 | 2.48(1.83-3.37) | 2.48(36.53) | 1.01(0.56) | 8.18995E-06 |
| Mean Cell Volume Increased* | 60 | 6.23(4.3-9.01) | 6.22(123.39) | 1.79(1.26) | 1.43829E-24 |
| Blood Test Abnormal | 58 | 2.4(1.76-3.27) | 2.4(32.9) | 0.98(0.52) | 5.13516E-05 |
| White Blood Cell Count Abnormal* | 57 | 2.88(2.09-3.96) | 2.87(45.77) | 1.16(0.68) | 8.19276E-08 |
| Red Cell Distribution Width Increased* | 57 | 2.68(1.95-3.68) | 2.68(40.31) | 1.09(0.62) | 1.2549E-06 |
| Night Sweats | 54 | 2.06(1.51-2.82) | 2.06(21.47) | 0.83(0.36) | 0.016961388 |
| Nervousness | 53 | 2.37(1.72-3.27) | 2.37(29.3) | 0.97(0.49) | 0.000325399 |
| Nasal Congestion* | 51 | 2.12(1.54-2.94) | 2.12(21.88) | 0.86(0.37) | 0.013988712 |
| Musculoskeletal Discomfort* | 49 | 3.55(2.48-5.08) | 3.54(54.42) | 1.35(0.81) | 1.19957E-09 |
| Mean Cell Haemoglobin Increased* | 48 | 5.74(3.83-8.6) | 5.74(91.87) | 1.73(1.14) | 1.07912E-17 |
| Tooth Disorder* | 47 | 2.15(1.54-3.02) | 2.15(20.86) | 0.87(0.37) | 0.024146843 |
| Immunodeficiency* | 46 | 2.39(1.69-3.37) | 2.39(25.82) | 0.98(0.46) | 0.001982416 |
| Crying* | 44 | 2.95(2.05-4.26) | 2.95(36.91) | 1.18(0.63) | 7.8135E-06 |
| Viral Infection | 40 | 2.89(1.97-4.24) | 2.89(32.47) | 1.16(0.59) | 7.51605E-05 |
| Immune System Disorder* | 38 | 2.55(1.73-3.74) | 2.55(24.41) | 1.04(0.46) | 0.004350075 |
| Pelvic Pain | 37 | 2.64(1.79-3.91) | 2.64(25.5) | 1.08(0.48) | 0.002555326 |
| Pain Of Skin* | 36 | 2.51(1.69-3.72) | 2.5(22.36) | 1.02(0.43) | 0.012451936 |
| Glomerular Filtration Rate Decreased* | 36 | 3.67(2.4-5.59) | 3.66(41.86) | 1.38(0.74) | 7.52579E-07 |
| Chromaturia* | 35 | 2.44(1.64-3.63) | 2.44(20.52) | 1(0.39) | 0.031829259 |
| Skin Hypopigmentation* | 35 | 24.06(11.16-51.87) | 24.05(143.86) | 2.4(1.56) | 1.41181E-28 |
| Head Discomfort* | 34 | 2.6(1.73-3.9) | 2.6(22.66) | 1.06(0.44) | 0.010998977 |
| Fear* | 30 | 3.44(2.18-5.42) | 3.44(31.88) | 1.32(0.62) | 0.00011879 |
| Menopausal Symptoms | 30 | 3.67(2.31-5.82) | 3.66(34.88) | 1.38(0.67) | 2.69375E-05 |
| Blood Urea Increased* | 27 | 2.75(1.73-4.36) | 2.75(20.02) | 1.11(0.41) | 0.045785409 |
| Lung Opacity* | 26 | 3.4(2.09-5.55) | 3.4(27.25) | 1.31(0.56) | 0.001286883 |
| Spinal Disorder* | 25 | 4.3(2.55-7.25) | 4.29(35.47) | 1.51(0.71) | 2.30069E-05 |
| White Blood Cell Disorder* | 25 | 3.72(2.24-6.17) | 3.71(29.59) | 1.39(0.61) | 0.00041518 |
| Vitiligo* | 24 | 7.76(4.17-14.45) | 7.76(58.61) | 1.93(1.01) | 2.98367E-10 |
| Hunger* | 24 | 5.5(3.12-9.68) | 5.5(44.15) | 1.7(0.84) | 3.41677E-07 |
| Renal Cyst* | 23 | 4.52(2.6-7.84) | 4.52(34.56) | 1.55(0.7) | 3.85663E-05 |
| Sensitive Skin* | 22 | 4.65(2.64-8.21) | 4.65(34.16) | 1.57(0.7) | 4.87977E-05 |
| Neutrophil Percentage Decreased | 21 | 16.5(7.01-38.81) | 16.49(76.4) | 2.28(1.19) | 9.82475E-09 |
| Platelet Count Abnormal* | 20 | 3.55(2.02-6.22) | 3.55(22.23) | 1.35(0.48) | 0.01815572 |
| Apathy* | 19 | 3.48(1.96-6.19) | 3.48(20.58) | 1.33(0.44) | 0.042352517 |
| Haemoglobin Increased* | 19 | 6.96(3.54-13.71) | 6.96(42.81) | 1.86(0.84) | 8.49299E-07 |
| Tongue Discomfort* | 19 | 4.18(2.3-7.59) | 4.18(26.09) | 1.49(0.56) | 0.00283228 |
| Anosmia* | 18 | 3.96(2.16-7.26) | 3.96(23.13) | 1.44(0.5) | 0.012538905 |
| Macrocytosis* | 18 | 5.21(2.73-9.93) | 5.21(31.43) | 1.66(0.66) | 0.00022187 |
| Middle Insomnia* | 18 | 4.5(2.41-8.39) | 4.5(26.93) | 1.55(0.58) | 0.001971443 |
| Body Mass Index Increased* | 18 | 7.07(3.52-14.21) | 7.07(41.02) | 1.87(0.81) | 0.000361845 |
| Lymphocyte Percentage Increased* | 18 | 32.99(9.72-112) | 32.98(79.75) | 2.48(1.21) | 5.73362E-09 |
| Blood Creatinine Abnormal | 18 | 4.12(2.24-7.6) | 4.12(24.33) | 1.48(0.52) | 0.006992724 |
| Superficial Vein Thrombosis | 18 | 6.6(3.32-13.09) | 6.6(38.85) | 1.83(0.78) | 6.12091E-06 |
| Anisocytosis* | 17 | 5.19(2.68-10.08) | 5.19(29.59) | 1.66(0.63) | 0.000570564 |
| Discouragement* | 17 | 5.5(2.81-10.77) | 5.5(31.27) | 1.7(0.66) | 0.000252819 |
| Feeling Of Despair* | 17 | 4.67(2.45-8.92) | 4.67(26.52) | 1.58(0.57) | 0.002518057 |
| Pulmonary Pain* | 15 | 5.5(2.69-11.25) | 5.5(27.59) | 1.7(0.58) | 0.028795329 |
| Basophil Count Increased* | 14 | 6.41(2.97-13.87) | 6.41(29.52) | 1.81(0.61) | 0.018687363 |
| Monocyte Percentage Increased* | 13 | 7.15(3.13-16.3) | 7.15(29.88) | 1.88(0.61) | 0.019742188 |
| Calcium Deficiency* | 12 | 13.19(4.65-37.45) | 13.19(39.77) | 2.2(0.74) | 0.001499845 |
| Myopia* | 11 | 12.09(4.2-34.81) | 12.09(34.98) | 2.16(0.64) | 0.006920642 |
| Blood Urea Abnormal* | 10 | 13.74(4.31-43.82) | 13.74(33.76) | 2.21(0.59) | 0.012055891 |
| Polychromasia* | 9 | 24.74(5.34-114.5) | 24.74(37.27) | 2.41(0.58) | 0.00583086 |
| Vertigo Positional* | 8 | 43.98(5.5-351.65) | 43.97(37.33) | 2.53(0.5) | 0.007208678 |
| Fat Tissue Increased* | 7 | 38.48(4.73-312.79) | 38.48(31.94) | 2.51(0.34) | 0.041736371 |

Abbreviation: ROR, reporting odds ratio; CI, confidence interval; PRR, proportional reporting ratio; χ^2^, Chi-Square; IC, information component; IC025, the lower limit of the 95% CI of the IC; *, unexpected signals that were not listed in the drug label.

Supplementary Table 15. The full results of Preferred Terms for Combination Therapy in patients weight ≥70 kg: A FAERS Database Study.

| **Preferred Terms** | **Cases** | **ROR(95%Cl)** | **PRR(χ2)** | **IC(IC025)** | **adjusted *P* value** |
| --- | --- | --- | --- | --- | --- |
| Fatigue | 1023 | 1.68(1.55-1.81) | 1.65(181.96) | 0.53(0.42) | 5.448E-38 |
| White Blood Cell Count Decreased | 724 | 2.24(2.04-2.46) | 2.21(294.08) | 0.79(0.66) | 2.33474E-62 |
| Neutropenia | 677 | 2.61(2.36-2.88) | 2.57(373.49) | 0.92(0.78) | 1.31562E-79 |
| Leukopenia | 380 | 2.95(2.57-3.38) | 2.92(259.32) | 1.02(0.84) | 1.06236E-54 |
| Arthralgia | 374 | 1.5(1.33-1.7) | 1.5(43.34) | 0.43(0.25) | 1.30435E-07 |
| Anaemia | 326 | 1.53(1.35-1.75) | 1.53(41.42) | 0.45(0.26) | 3.52209E-07 |
| Malaise | 293 | 1.41(1.23-1.62) | 1.41(24.6) | 0.36(0.17) | 0.001928021 |
| Dizziness | 258 | 1.47(1.27-1.71) | 1.47(27.13) | 0.41(0.2) | 0.000531632 |
| Constipation | 226 | 1.48(1.27-1.73) | 1.48(24.31) | 0.41(0.19) | 0.002305372 |
| Weight Decreased | 224 | 1.46(1.25-1.71) | 1.46(22.83) | 0.4(0.18) | 0.004933386 |
| Thrombocytopenia | 204 | 1.56(1.32-1.85) | 1.56(28.1) | 0.47(0.23) | 0.000334995 |
| Weight Increased | 201 | 1.9(1.6-2.26) | 1.89(54.32) | 0.65(0.41) | 5.58096E-10 |
| Neutrophil Count Decreased | 201 | 2.29(1.91-2.74) | 2.28(86.39) | 0.82(0.57) | 5.48185E-17 |
| Hot Flush | 193 | 1.53(1.29-1.81) | 1.52(24) | 0.44(0.2) | 0.002756992 |
| Haemoglobin Decreased | 181 | 1.96(1.64-2.36) | 1.96(54) | 0.68(0.43) | 6.71457E-10 |
| Nasopharyngitis | 168 | 1.9(1.57-2.29) | 1.89(45.4) | 0.65(0.39) | 5.27285E-08 |
| Red Blood Cell Count Decreased | 167 | 2.43(1.99-2.96) | 2.42(81.35) | 0.87(0.59) | 7.29349E-16 |
| Platelet Count Decreased | 161 | 1.54(1.28-1.86) | 1.54(21.04) | 0.46(0.19) | 0.012954504 |
| Full Blood Count Abnormal* | 144 | 2.94(2.35-3.67) | 2.93(98.24) | 1.02(0.72) | 1.63374E-19 |
| Blood Creatinine Increased | 136 | 2.31(1.86-2.88) | 2.31(60.05) | 0.83(0.52) | 3.46455E-11 |
| Feeling Abnormal* | 135 | 1.85(1.5-2.28) | 1.85(33.94) | 0.63(0.33) | 1.83805E-05 |
| Dry Skin | 127 | 2.12(1.7-2.64) | 2.11(45.91) | 0.75(0.44) | 4.36376E-08 |
| Full Blood Count Decreased* | 117 | 2.91(2.27-3.73) | 2.91(78.75) | 1.02(0.68) | 3.0964E-15 |
| Alanine Aminotransferase Increased | 114 | 1.68(1.34-2.1) | 1.67(20.74) | 0.54(0.22) | 0.016014311 |
| Electrocardiogram Qt Prolonged | 103 | 4.84(3.58-6.55) | 4.83(128.94) | 1.37(0.98) | 4.39393E-26 |
| Illness* | 102 | 2.58(1.99-3.33) | 2.57(55.69) | 0.92(0.56) | 3.42681E-10 |
| Influenza | 82 | 2.48(1.86-3.29) | 2.47(41.59) | 0.89(0.49) | 4.43837E-07 |
| Dyspnoea Exertional* | 80 | 1.88(1.43-2.47) | 1.88(21.09) | 0.64(0.25) | 0.014405281 |
| Decreased Immune Responsiveness* | 76 | 4.36(3.1-6.13) | 4.35(85.8) | 1.3(0.85) | 1.16447E-16 |
| Stress* | 71 | 2.76(2.02-3.78) | 2.76(43.8) | 0.98(0.54) | 1.54119E-07 |
| Oropharyngeal Pain | 71 | 1.94(1.45-2.59) | 1.93(20.39) | 0.67(0.26) | 0.02114302 |
| Hepatotoxicity | 67 | 2.6(1.89-3.58) | 2.6(37.35) | 0.93(0.48) | 4.03987E-06 |
| Haematocrit Decreased | 64 | 2.38(1.73-3.28) | 2.38(29.96) | 0.85(0.4) | 0.000169559 |
| Sleep Disorder* | 63 | 2.03(1.48-2.77) | 2.03(20.5) | 0.72(0.27) | 0.020621809 |
| Depressed Mood* | 60 | 2.54(1.81-3.55) | 2.53(31.85) | 0.91(0.43) | 6.69572E-05 |
| Hypoacusis* | 56 | 2.34(1.66-3.29) | 2.34(25.3) | 0.84(0.36) | 0.001866393 |
| Cystitis | 49 | 2.55(1.76-3.69) | 2.55(26.25) | 0.91(0.39) | 0.001208965 |
| Liver Function Test Increased | 48 | 2.8(1.91-4.1) | 2.8(30.31) | 0.99(0.45) | 0.000158939 |
| Ageusia | 47 | 2.48(1.7-3.62) | 2.48(23.96) | 0.89(0.35) | 0.003897831 |
| Osteoarthritis* | 47 | 2.79(1.89-4.1) | 2.79(29.49) | 0.98(0.44) | 0.000241076 |
| Mean Cell Volume Increased* | 47 | 4.18(2.73-6.41) | 4.18(50.81) | 1.28(0.7) | 5.78441E-09 |
| Blood Test Abnormal | 38 | 2.99(1.93-4.62) | 2.98(26.64) | 1.04(0.42) | 0.001107265 |
| Glomerular Filtration Rate Decreased* | 37 | 2.98(1.91-4.63) | 2.98(25.81) | 1.04(0.41) | 0.001695764 |
| White Blood Cell Count Abnormal* | 34 | 3.38(2.1-5.44) | 3.38(28.47) | 1.13(0.46) | 0.000470308 |
| Cytopenia* | 28 | 3.15(1.88-5.28) | 3.15(21.29) | 1.08(0.35) | 0.018586985 |
| Mean Cell Haemoglobin Increased* | 28 | 3.5(2.07-5.95) | 3.5(24.59) | 1.16(0.41) | 0.003599304 |
| Musculoskeletal Discomfort* | 27 | 4.15(2.36-7.28) | 4.15(28.94) | 1.27(0.49) | 0.000425697 |
| White Blood Cell Disorder* | 22 | 5.31(2.72-10.38) | 5.31(29.92) | 1.42(0.52) | 0.000304496 |
| Hiatus Hernia* | 21 | 4.44(2.31-8.5) | 4.43(24.15) | 1.31(0.41) | 0.005340688 |
| Macrocytosis* | 18 | 6.76(3.04-15.05) | 6.76(29.42) | 1.55(0.5) | 0.000459413 |
| Vertigo Positional* | 12 | 20.27(4.54-90.59) | 20.27(31.4) | 1.91(0.46) | 0.002568623 |
| Anaemia Macrocytic* | 11 | 12.39(3.46-44.41) | 12.38(24.67) | 1.78(0.32) | 0.03574049 |
| Neutrophil Percentage Decreased | 9 | 30.41(3.85-240.02) | 30.4(25.59) | 1.98(0.25) | 0.030212646 |

Abbreviation: ROR, reporting odds ratio; CI, confidence interval; PRR, proportional reporting ratio; χ^2^, Chi-Square; IC, information component; IC025, the lower limit of the 95% CI of the IC; *, unexpected signals that were not listed in the drug label.

Supplementary Table 16. The full results of Preferred Terms for Combination Therapy in patients weight ＜70 kg: A FAERS Database Study.

| **Preferred Terms** | **Cases** | **ROR(95%Cl)** | **PRR(χ2)** | **IC(IC025)** | **adjusted *P* value** |
| --- | --- | --- | --- | --- | --- |
| Fatigue | 865 | 1.58(1.46-1.71) | 1.57(128.91) | 0.49(0.38) | 2.15176E-26 |
| Neutropenia | 739 | 1.98(1.81-2.17) | 1.96(235.12) | 0.71(0.59) | 1.60515E-49 |
| White Blood Cell Count Decreased | 737 | 1.43(1.32-1.56) | 1.42(69.32) | 0.39(0.27) | 2.39492E-13 |
| Anaemia | 354 | 1.38(1.23-1.56) | 1.38(27.59) | 0.36(0.18) | 0.000420558 |
| Leukopenia | 318 | 1.82(1.6-2.08) | 1.82(80.19) | 0.64(0.45) | 1.13371E-15 |
| Arthralgia | 297 | 1.39(1.22-1.59) | 1.39(24.06) | 0.36(0.17) | 0.002628578 |
| Dizziness | 271 | 1.36(1.18-1.56) | 1.36(19.12) | 0.34(0.14) | 0.034248404 |
| Haemoglobin Decreased | 231 | 1.92(1.64-2.24) | 1.91(67.72) | 0.69(0.46) | 6.50852E-13 |
| Thrombocytopenia | 220 | 1.55(1.33-1.81) | 1.55(30.59) | 0.48(0.25) | 9.60752E-05 |
| General Physical Health Deterioration* | 217 | 1.48(1.26-1.73) | 1.47(24.23) | 0.43(0.2) | 0.002497533 |
| Pain In Extremity | 214 | 1.44(1.23-1.69) | 1.44(21.11) | 0.4(0.18) | 0.012479278 |
| Pleural Effusion | 168 | 1.48(1.24-1.76) | 1.47(18.63) | 0.43(0.17) | 0.046406207 |
| Urinary Tract Infection | 166 | 1.72(1.43-2.06) | 1.72(34.63) | 0.58(0.32) | 1.28268E-05 |
| Red Blood Cell Count Decreased | 146 | 2.2(1.8-2.7) | 2.2(61.24) | 0.82(0.53) | 1.92168E-11 |
| Dry Skin | 141 | 1.55(1.27-1.88) | 1.54(19.4) | 0.47(0.19) | 0.032058933 |
| Nasopharyngitis | 138 | 2.08(1.69-2.56) | 2.08(50.54) | 0.77(0.47) | 4.28535E-09 |
| Full Blood Count Abnormal* | 135 | 2.94(2.35-3.67) | 2.93(98.49) | 1.07(0.76) | 1.53726E-19 |
| Feeling Abnormal* | 127 | 1.92(1.55-2.38) | 1.92(37.63) | 0.69(0.39) | 2.97398E-06 |
| Blood Creatinine Increased | 126 | 2.49(1.99-3.12) | 2.49(68.75) | 0.93(0.62) | 4.66703E-13 |
| Full Blood Count Decreased* | 110 | 2.52(1.98-3.2) | 2.51(61.18) | 0.94(0.6) | 2.18837E-11 |
| Decreased Immune Responsiveness* | 107 | 3.7(2.84-4.81) | 3.69(108.23) | 1.25(0.89) | 1.34108E-21 |
| Electrocardiogram Qt Prolonged | 101 | 4.23(3.19-5.6) | 4.22(119.8) | 1.35(0.97) | 4.35183E-24 |
| Memory Impairment* | 99 | 1.73(1.37-2.19) | 1.73(21.15) | 0.59(0.25) | 0.0138226 |
| Gamma-Glutamyltransferase Increased | 80 | 1.94(1.49-2.54) | 1.94(24.42) | 0.7(0.32) | 0.002716966 |
| Sleep Disorder* | 76 | 2.17(1.64-2.87) | 2.16(30.7) | 0.81(0.4) | 0.000113951 |
| Renal Impairment* | 74 | 2.08(1.57-2.76) | 2.08(27.05) | 0.77(0.36) | 0.00073089 |
| Lacrimation Increased | 65 | 2.32(1.71-3.16) | 2.32(30.76) | 0.87(0.43) | 0.000116405 |
| Inflammation* | 64 | 2.1(1.55-2.84) | 2.1(23.92) | 0.78(0.34) | 0.003731058 |
| Haematocrit Decreased | 59 | 2.52(1.82-3.5) | 2.52(32.96) | 0.95(0.47) | 3.97228E-05 |
| Blood Alkaline Phosphatase Increased | 57 | 2.09(1.52-2.89) | 2.09(21.24) | 0.78(0.31) | 0.015066993 |
| Depressed Mood* | 55 | 2.23(1.6-3.1) | 2.23(23.76) | 0.83(0.35) | 0.004232168 |
| Spinal Pain* | 51 | 2.23(1.58-3.14) | 2.23(22) | 0.83(0.33) | 0.010578628 |
| Taste Disorder | 43 | 2.45(1.67-3.59) | 2.45(22.71) | 0.92(0.37) | 0.007852937 |
| Red Cell Distribution Width Increased* | 42 | 3.51(2.32-5.33) | 3.51(39.84) | 1.22(0.62) | 1.49867E-06 |
| Mean Cell Volume Increased* | 40 | 6.29(3.82-10.37) | 6.29(68.4) | 1.6(0.94) | 1.12438E-12 |
| Laboratory Test Abnormal | 37 | 3.23(2.09-4.99) | 3.23(31.27) | 1.15(0.53) | 0.000114449 |
| Mean Cell Haemoglobin Increased* | 33 | 4.47(2.72-7.37) | 4.47(41.59) | 1.39(0.69) | 7.34948E-07 |
| White Blood Cell Count Abnormal* | 31 | 3.58(2.2-5.83) | 3.58(30.19) | 1.23(0.53) | 0.000217356 |
| Chronic Kidney Disease* | 30 | 3.69(2.24-6.07) | 3.68(30.27) | 1.25(0.54) | 0.000212569 |
| Glomerular Filtration Rate Decreased* | 30 | 3.37(2.07-5.49) | 3.37(26.89) | 1.19(0.48) | 0.001137871 |
| Abdominal Pain Lower | 29 | 3.93(2.35-6.58) | 3.93(31.66) | 1.3(0.56) | 0.00010907 |
| Vitamin D Deficiency | 29 | 4.75(2.77-8.16) | 4.75(38.85) | 1.43(0.67) | 3.1428E-06 |
| General Physical Condition Abnormal* | 27 | 4.08(2.38-7) | 4.08(30.8) | 1.33(0.56) | 0.000175295 |
| Erysipelas* | 26 | 5.68(3.11-10.36) | 5.67(40.96) | 1.54(0.71) | 1.21591E-06 |
| Eastern Cooperative Oncology Group Performance Status Worsened* | 25 | 3.39(1.98-5.79) | 3.39(22.59) | 1.19(0.41) | 0.010701855 |
| Sensitive Skin* | 19 | 4.39(2.28-8.45) | 4.39(23.5) | 1.38(0.43) | 0.008144 |
| Gastrointestinal Infection* | 19 | 10.67(4.48-25.38) | 10.66(44.8) | 1.85(0.77) | 2.6271E-07 |
| Neutrophil Count Abnormal | 19 | 6.22(3.02-12.82) | 6.22(32.22) | 1.59(0.59) | 0.000115594 |
| Blood Calcium Increased | 18 | 5.05(2.51-10.16) | 5.05(25.59) | 1.47(0.47) | 0.003056365 |
| Increased Appetite | 17 | 5.14(2.5-10.58) | 5.14(24.55) | 1.48(0.45) | 0.005313266 |
| Restless Legs Syndrome* | 16 | 5.24(2.48-11.08) | 5.24(23.51) | 1.49(0.42) | 0.009225676 |
| Calcium Deficiency* | 15 | 7.37(3.12-17.38) | 7.37(28.71) | 1.68(0.52) | 0.008662785 |
| Neutrophil Percentage Decreased | 15 | 11.79(4.28-32.44) | 11.78(37.01) | 1.89(0.65) | 0.000513627 |
| Monocyte Percentage Increased* | 13 | 8.51(3.24-22.4) | 8.51(27.22) | 1.75(0.46) | 0.017965184 |
| Body Mass Index Increased* | 10 | 19.65(4.3-89.67) | 19.64(29.49) | 2.04(0.41) | 0.012133467 |
| Peripheral Artery Thrombosis* | 10 | 13.1(3.6-47.59) | 13.09(25.78) | 1.92(0.36) | 0.042918687 |
| Body Surface Area Decreased* | 8 | 31.43(3.93-251.33) | 31.43(26.19) | 2.13(0.25) | 0.049517442 |

Abbreviation: ROR, reporting odds ratio; CI, confidence interval; PRR, proportional reporting ratio; χ^2^, Chi-Square; IC, information component; IC025, the lower limit of the 95% CI of the IC; *, unexpected signals that were not listed in the drug label.

Supplementary Table 17. The full results of Preferred Terms for Combination Therapy in patients excluding other medications: A FAERS Database Study.

| **Preferred Terms** | **Cases** | **ROR(95%Cl)** | **PRR(χ2)** | **IC(IC025)** | **adjusted *P* value** |
| --- | --- | --- | --- | --- | --- |
| Fatigue | 4242 | 1.39(1.35-1.44) | 1.38(362.84) | 0.38(0.33) | 3.24026E-77 |
| Neutropenia | 3107 | 2.3(2.2-2.4) | 2.27(1550.19) | 0.91(0.85) | 0 |
| White Blood Cell Count Decreased | 3014 | 2.14(2.05-2.24) | 2.12(1276.28) | 0.84(0.78) | 1.039E-275 |
| Arthralgia | 1438 | 1.64(1.55-1.74) | 1.63(271.32) | 0.57(0.48) | 3.11587E-57 |
| Decreased Appetite | 1277 | 1.18(1.11-1.26) | 1.18(28.77) | 0.2(0.11) | 0.000354386 |
| Anaemia | 1238 | 1.39(1.31-1.48) | 1.39(107.23) | 0.39(0.3) | 1.89751E-21 |
| Malaise | 1226 | 1.35(1.27-1.44) | 1.34(86.74) | 0.35(0.26) | 5.82087E-17 |
| Dizziness | 1038 | 1.39(1.29-1.49) | 1.38(87.58) | 0.38(0.28) | 3.87212E-17 |
| Leukopenia | 1036 | 2.14(1.99-2.3) | 2.13(443.05) | 0.85(0.75) | 1.5148E-94 |
| Constipation | 954 | 1.26(1.18-1.36) | 1.26(42.07) | 0.28(0.17) | 3.98537E-07 |
| Cough | 910 | 1.21(1.13-1.3) | 1.21(27.47) | 0.23(0.12) | 0.000706945 |
| Pain In Extremity | 885 | 1.46(1.36-1.57) | 1.46(99.69) | 0.44(0.33) | 8.78359E-20 |
| Weight Decreased | 871 | 1.24(1.15-1.33) | 1.24(31.86) | 0.25(0.14) | 7.40313E-05 |
| Neutrophil Count Decreased | 862 | 1.87(1.73-2.02) | 1.86(254.84) | 0.71(0.6) | 1.33153E-53 |
| Back Pain | 852 | 1.41(1.31-1.52) | 1.41(80.2) | 0.4(0.29) | 1.63757E-15 |
| Haemoglobin Decreased | 852 | 2.27(2.09-2.46) | 2.26(417.86) | 0.91(0.79) | 4.80165E-89 |
| Pruritus | 815 | 1.28(1.19-1.39) | 1.28(41.03) | 0.3(0.18) | 6.87048E-07 |
| Platelet Count Decreased | 805 | 1.46(1.35-1.58) | 1.46(91.63) | 0.44(0.33) | 5.16081E-18 |
| Hot Flush | 714 | 1.83(1.68-1.99) | 1.83(197.92) | 0.69(0.56) | 3.41049E-41 |
| Thrombocytopenia | 681 | 1.22(1.13-1.33) | 1.22(22.61) | 0.24(0.12) | 0.008847909 |
| Bone Pain | 641 | 1.38(1.26-1.51) | 1.38(52.79) | 0.38(0.25) | 1.77228E-09 |
| Red Blood Cell Count Decreased | 619 | 2.45(2.22-2.69) | 2.44(358.59) | 0.99(0.85) | 4.0781E-76 |
| Insomnia | 596 | 1.44(1.31-1.57) | 1.43(61.6) | 0.42(0.29) | 2.05791E-11 |
| General Physical Health Deterioration* | 570 | 1.54(1.4-1.69) | 1.54(83.34) | 0.5(0.37) | 3.53542E-16 |
| Dry Skin | 568 | 1.61(1.47-1.77) | 1.61(100.99) | 0.55(0.42) | 4.87149E-20 |
| Nasopharyngitis | 563 | 1.98(1.8-2.18) | 1.98(197.41) | 0.77(0.63) | 4.66596E-41 |
| Decreased Immune Responsiveness* | 539 | 3.91(3.5-4.37) | 3.9(663.97) | 1.41(1.25) | 2.1894E-142 |
| Urinary Tract Infection | 524 | 1.41(1.28-1.55) | 1.41(48.41) | 0.4(0.26) | 1.66831E-08 |
| Full Blood Count Abnormal* | 514 | 2.59(2.33-2.88) | 2.58(332.92) | 1.04(0.89) | 1.66146E-70 |
| Feeling Abnormal* | 505 | 1.76(1.59-1.94) | 1.75(122.77) | 0.65(0.5) | 8.7352E-25 |
| Weight Increased | 481 | 1.92(1.73-2.14) | 1.92(155.25) | 0.74(0.59) | 7.33824E-32 |
| Abdominal Discomfort | 478 | 1.46(1.32-1.61) | 1.46(53.47) | 0.44(0.29) | 1.29978E-09 |
| Blood Creatinine Increased | 464 | 2.27(2.04-2.53) | 2.27(228.83) | 0.91(0.75) | 7.25591E-48 |
| Full Blood Count Decreased* | 448 | 2.55(2.28-2.86) | 2.54(282.25) | 1.03(0.86) | 1.79269E-59 |
| Memory Impairment* | 426 | 1.66(1.49-1.86) | 1.66(85.14) | 0.59(0.43) | 1.49752E-16 |
| Gait Disturbance* | 407 | 1.34(1.2-1.5) | 1.34(28.28) | 0.35(0.19) | 0.000494052 |
| Alanine Aminotransferase Increased | 393 | 1.52(1.35-1.7) | 1.51(53.28) | 0.48(0.32) | 1.46887E-09 |
| Aspartate Aminotransferase Increased | 375 | 1.53(1.36-1.71) | 1.53(52.57) | 0.49(0.32) | 2.11901E-09 |
| Electrocardiogram Qt Prolonged | 350 | 3.06(2.68-3.5) | 3.06(305.47) | 1.2(1.01) | 1.82762E-64 |
| Influenza | 314 | 2.23(1.95-2.54) | 2.22(148.2) | 0.89(0.7) | 2.85584E-30 |
| Visual Impairment | 298 | 1.6(1.41-1.82) | 1.6(51.28) | 0.54(0.36) | 4.21214E-09 |
| Somnolence | 287 | 1.4(1.23-1.59) | 1.39(25.33) | 0.39(0.2) | 0.002332655 |
| Oropharyngeal Pain | 284 | 1.57(1.37-1.79) | 1.57(44.77) | 0.52(0.33) | 1.14945E-07 |
| Hepatotoxicity | 261 | 2.09(1.81-2.42) | 2.09(105.98) | 0.83(0.62) | 4.7202E-21 |
| Renal Impairment* | 256 | 1.47(1.28-1.69) | 1.47(29.85) | 0.45(0.25) | 0.000232847 |
| Sleep Disorder* | 235 | 2.17(1.86-2.53) | 2.17(104.25) | 0.87(0.65) | 1.16497E-20 |
| Depressed Mood* | 220 | 1.87(1.6-2.18) | 1.87(65.42) | 0.71(0.49) | 3.54241E-12 |
| Gamma-Glutamyltransferase Increased | 220 | 2(1.71-2.34) | 2(79.47) | 0.78(0.56) | 3.00936E-15 |
| Atrial Fibrillation* | 211 | 1.43(1.23-1.66) | 1.43(21.1) | 0.42(0.19) | 0.021386804 |
| Haematocrit Decreased | 204 | 2.52(2.13-2.98) | 2.52(125.52) | 1.01(0.77) | 2.87044E-25 |
| Stress* | 202 | 2.27(1.93-2.68) | 2.27(99.89) | 0.91(0.67) | 1.09124E-19 |
| Transaminases Increased | 198 | 1.59(1.36-1.87) | 1.59(33.3) | 0.54(0.31) | 4.14644E-05 |
| Rhinorrhoea* | 187 | 1.47(1.25-1.73) | 1.47(21.97) | 0.45(0.21) | 0.013811476 |
| Spinal Pain* | 184 | 2.41(2.03-2.88) | 2.41(103.9) | 0.97(0.72) | 1.51322E-20 |
| Taste Disorder | 176 | 1.56(1.32-1.85) | 1.56(27.15) | 0.52(0.27) | 0.000975241 |
| Cataract | 173 | 1.62(1.36-1.92) | 1.62(30.93) | 0.55(0.31) | 0.000141576 |
| Dyspnoea Exertional* | 170 | 1.54(1.3-1.83) | 1.54(25.04) | 0.5(0.25) | 0.002891807 |
| Liver Function Test Increased | 168 | 1.58(1.33-1.88) | 1.58(27.64) | 0.53(0.28) | 0.000765652 |
| Inflammation* | 166 | 1.9(1.59-2.27) | 1.9(52.01) | 0.73(0.47) | 3.27286E-09 |
| Eating Disorder* | 147 | 1.6(1.33-1.92) | 1.6(25.06) | 0.54(0.27) | 0.002927902 |
| Bronchitis | 143 | 1.81(1.5-2.19) | 1.81(38.49) | 0.68(0.4) | 3.16358E-06 |
| Cystitis | 141 | 1.87(1.54-2.27) | 1.87(42.11) | 0.71(0.43) | 5.07661E-07 |
| Productive Cough* | 139 | 1.81(1.5-2.2) | 1.81(37.57) | 0.68(0.4) | 5.06715E-06 |
| Sinusitis | 135 | 1.64(1.35-1.99) | 1.64(25.47) | 0.57(0.29) | 0.002407344 |
| Hepatic Cytolysis* | 135 | 1.57(1.3-1.91) | 1.57(21.53) | 0.52(0.24) | 0.018161971 |
| Laboratory Test Abnormal | 131 | 1.75(1.43-2.13) | 1.75(31.39) | 0.64(0.35) | 0.000118424 |
| Musculoskeletal Chest Pain* | 131 | 1.65(1.36-2.01) | 1.65(25.49) | 0.58(0.29) | 0.002391695 |
| Mean Cell Volume Increased* | 127 | 4.85(3.81-6.18) | 4.85(200.59) | 1.58(1.24) | 2.02625E-41 |
| Ageusia | 126 | 1.67(1.37-2.04) | 1.67(25.55) | 0.59(0.3) | 0.002341505 |
| Breast Pain | 126 | 1.88(1.53-2.31) | 1.88(38.08) | 0.72(0.42) | 3.99328E-06 |
| Renal Disorder | 125 | 1.96(1.6-2.41) | 1.96(42.7) | 0.76(0.46) | 3.87438E-07 |
| Movement Disorder* | 125 | 2.22(1.8-2.74) | 2.22(58.85) | 0.89(0.59) | 1.13235E-10 |
| Glomerular Filtration Rate Decreased* | 122 | 3.32(2.64-4.16) | 3.32(120.43) | 1.27(0.94) | 4.71688E-24 |
| White Blood Cell Count Abnormal* | 121 | 2.45(1.98-3.05) | 2.45(70.77) | 0.99(0.67) | 2.88747E-13 |
| Migraine* | 120 | 1.66(1.35-2.04) | 1.66(23.69) | 0.58(0.28) | 0.006129319 |
| Cytopenia* | 115 | 1.91(1.54-2.36) | 1.91(36.3) | 0.73(0.42) | 1.00597E-05 |
| Red Cell Distribution Width Increased* | 114 | 2.43(1.94-3.03) | 2.43(65.1) | 0.98(0.65) | 5.0906E-12 |
| Nervousness | 111 | 1.77(1.43-2.19) | 1.77(27.6) | 0.65(0.34) | 0.00084183 |
| Fluid Retention* | 109 | 1.81(1.45-2.25) | 1.81(29.19) | 0.68(0.36) | 0.000377646 |
| Osteoarthritis* | 108 | 2.19(1.75-2.74) | 2.19(49.12) | 0.88(0.55) | 1.58656E-08 |
| Limb Discomfort* | 107 | 1.7(1.37-2.11) | 1.7(23.17) | 0.61(0.29) | 0.008169175 |
| Nasal Congestion* | 104 | 1.97(1.57-2.47) | 1.97(36.03) | 0.77(0.44) | 1.18573E-05 |
| Viral Infection | 99 | 2.51(1.97-3.19) | 2.51(60.48) | 1.01(0.66) | 5.45438E-11 |
| Hepatic Steatosis* | 96 | 1.72(1.37-2.17) | 1.72(21.93) | 0.63(0.29) | 0.015732437 |
| Mean Cell Haemoglobin Increased* | 91 | 4.38(3.31-5.78) | 4.37(128.55) | 1.5(1.1) | 1.01434E-25 |
| Musculoskeletal Discomfort* | 90 | 2.69(2.08-3.46) | 2.68(62.74) | 1.08(0.7) | 1.83697E-11 |
| Chronic Kidney Disease* | 88 | 2.57(1.99-3.31) | 2.57(56.3) | 1.03(0.66) | 4.64845E-10 |
| Immune System Disorder* | 88 | 2.1(1.64-2.68) | 2.1(35.91) | 0.83(0.47) | 1.32312E-05 |
| Immunodeficiency* | 86 | 1.98(1.55-2.54) | 1.98(30.37) | 0.78(0.41) | 0.00021893 |
| Breast Mass | 83 | 1.9(1.48-2.44) | 1.9(25.83) | 0.73(0.36) | 0.002224065 |
| Crying* | 78 | 2.14(1.65-2.79) | 2.14(33.63) | 0.85(0.47) | 4.3299E-05 |
| Osteopenia | 74 | 1.92(1.47-2.51) | 1.92(23.84) | 0.74(0.35) | 0.006296347 |
| Body Height Decreased* | 73 | 2.38(1.81-3.15) | 2.38(40.17) | 0.96(0.55) | 1.64437E-06 |
| Pelvic Pain | 70 | 2.31(1.75-3.07) | 2.31(36.15) | 0.93(0.52) | 1.2661E-05 |
| Pain Of Skin* | 66 | 1.94(1.46-2.57) | 1.94(21.74) | 0.75(0.33) | 0.01887878 |
| Erysipelas* | 66 | 2.41(1.8-3.23) | 2.41(37.27) | 0.97(0.54) | 7.33577E-06 |
| Fear* | 62 | 3.19(2.32-4.37) | 3.19(57.64) | 1.24(0.77) | 2.82768E-10 |
| Skin Hypopigmentation* | 62 | 32.2(16.51-62.79) | 32.18(260.19) | 2.41(1.8) | 8.19531E-54 |
| Blood Pressure Abnormal* | 61 | 2.25(1.66-3.03) | 2.25(29.43) | 0.9(0.46) | 0.000393162 |
| Vitiligo* | 59 | 5.78(3.99-8.38) | 5.78(110.33) | 1.71(1.18) | 1.27414E-21 |
| Head Discomfort* | 58 | 1.99(1.47-2.7) | 1.99(20.76) | 0.78(0.33) | 0.032243594 |
| Increased Appetite | 53 | 2.18(1.58-3.01) | 2.18(23.93) | 0.87(0.4) | 0.00661829 |
| Eastern Cooperative Oncology Group Performance Status Worsened* | 53 | 2.62(1.88-3.65) | 2.62(35.29) | 1.05(0.56) | 2.17552E-05 |
| Platelet Count Increased* | 52 | 2.21(1.6-3.06) | 2.21(24.23) | 0.89(0.4) | 0.005733728 |
| Neutrophil Count Abnormal | 50 | 2.16(1.56-3.01) | 2.16(22.07) | 0.86(0.37) | 0.01734931 |
| Blood Calcium Increased | 49 | 2.45(1.74-3.44) | 2.45(28.47) | 0.99(0.48) | 0.000691254 |
| Vitamin D Deficiency | 49 | 3.14(2.2-4.48) | 3.14(44.54) | 1.22(0.69) | 2.22439E-07 |
| White Blood Cell Disorder* | 49 | 3.1(2.18-4.42) | 3.1(43.69) | 1.21(0.68) | 3.39535E-07 |
| Spinal Disorder* | 44 | 3.05(2.1-4.42) | 3.05(38.1) | 1.19(0.64) | 5.85006E-06 |
| Intervertebral Disc Protrusion* | 43 | 2.83(1.95-4.1) | 2.83(32.84) | 1.13(0.57) | 8.16581E-05 |
| Osteosclerosis* | 42 | 2.66(1.83-3.86) | 2.66(28.74) | 1.07(0.51) | 0.000641469 |
| Platelet Count Abnormal* | 42 | 2.34(1.63-3.38) | 2.34(22.3) | 0.95(0.4) | 0.016353915 |
| Gastrointestinal Infection* | 42 | 3.76(2.53-5.59) | 3.76(49.33) | 1.38(0.79) | 2.25767E-08 |
| General Physical Condition Abnormal* | 41 | 3.08(2.1-4.54) | 3.08(36.23) | 1.21(0.63) | 1.5414E-05 |
| Hunger* | 40 | 3.46(2.32-5.16) | 3.46(41.99) | 1.31(0.71) | 8.90058E-07 |
| Sensitive Skin* | 40 | 3.01(2.04-4.44) | 3.01(33.97) | 1.18(0.6) | 4.81752E-05 |
| Renal Cyst* | 38 | 2.78(1.87-4.12) | 2.78(28.17) | 1.11(0.52) | 0.000894084 |
| Neutrophil Percentage Decreased | 38 | 16.44(8.59-31.46) | 16.44(132.23) | 2.23(1.46) | 4.8278E-26 |
| Anosmia* | 36 | 3.4(2.23-5.17) | 3.4(36.82) | 1.29(0.66) | 1.24175E-05 |
| Macrocytosis* | 36 | 3.11(2.06-4.71) | 3.11(32.3) | 1.22(0.59) | 0.000117119 |
| Discouragement* | 34 | 4.9(3.07-7.84) | 4.9(54.32) | 1.59(0.89) | 2.26323E-09 |
| Haemoglobin Increased* | 32 | 5.36(3.27-8.78) | 5.36(55.82) | 1.65(0.92) | 1.14481E-09 |
| Middle Insomnia* | 31 | 2.82(1.82-4.37) | 2.82(23.64) | 1.12(0.46) | 0.009510913 |
| Blood Creatinine Abnormal | 31 | 2.82(1.82-4.37) | 2.82(23.64) | 1.12(0.46) | 0.009510913 |
| Anisocytosis* | 30 | 2.88(1.85-4.51) | 2.88(23.73) | 1.14(0.47) | 0.009230685 |
| Hyperuricaemia* | 30 | 3.8(2.37-6.08) | 3.8(35.71) | 1.39(0.68) | 2.41579E-05 |
| Lymphocyte Percentage Increased* | 30 | 17.31(8.22-36.45) | 17.3(106.34) | 2.25(1.36) | 2.32077E-20 |
| Monocyte Percentage Increased* | 30 | 7.79(4.42-13.71) | 7.79(70.98) | 1.89(1.09) | 7.28899E-13 |
| Thrombophlebitis | 28 | 3.09(1.94-4.94) | 3.09(24.84) | 1.21(0.5) | 0.005506105 |
| Basophil Count Increased* | 28 | 3.63(2.24-5.89) | 3.63(31.44) | 1.35(0.62) | 0.000209292 |
| Feeling Of Despair* | 28 | 3.73(2.29-6.06) | 3.73(32.52) | 1.37(0.63) | 0.000122868 |
| Lymphocyte Count Increased* | 28 | 4.15(2.53-6.83) | 4.15(37.23) | 1.46(0.71) | 1.2008E-05 |
| Body Mass Index Increased* | 27 | 7.01(3.93-12.5) | 7.01(59.17) | 1.83(0.99) | 2.65272E-10 |
| Anaemia Macrocytic* | 25 | 4.19(2.47-7.09) | 4.19(33.56) | 1.47(0.67) | 7.97673E-05 |
| Red Blood Cell Count Abnormal* | 24 | 3.04(1.84-5.03) | 3.04(20.7) | 1.19(0.42) | 0.046872821 |
| Creatinine Renal Clearance Decreased* | 24 | 3.04(1.84-5.03) | 3.04(20.7) | 1.19(0.42) | 0.046872821 |
| Superficial Vein Thrombosis | 22 | 4.23(2.41-7.43) | 4.23(29.89) | 1.47(0.61) | 0.000532851 |
| Calcium Deficiency* | 21 | 6.41(3.38-12.16) | 6.41(42.92) | 1.77(0.82) | 9.71543E-07 |
| Vertigo Positional* | 20 | 20.77(7.79-55.33) | 20.76(75.24) | 2.31(1.17) | 1.34143E-08 |
| Feeling Of Body Temperature Change* | 20 | 3.71(2.09-6.58) | 3.71(23.07) | 1.37(0.48) | 0.016255848 |
| Alanine Aminotransferase Decreased* | 15 | 7.08(3.25-15.41) | 7.08(33.12) | 1.84(0.67) | 0.006763948 |
| Body Surface Area Increased* | 14 | 6.61(3-14.55) | 6.61(29.31) | 1.79(0.59) | 0.024393819 |
| Body Surface Area Decreased* | 13 | 9.64(3.85-24.16) | 9.64(35.23) | 2.01(0.68) | 0.005057555 |
| Mean Platelet Volume Increased* | 13 | 6.75(2.96-15.39) | 6.75(27.67) | 1.81(0.55) | 0.046030294 |
| Plicated Tongue* | 11 | 19.03(5.31-68.23) | 19.03(40.27) | 2.28(0.69) | 0.00175051 |

Abbreviation: ROR, reporting odds ratio; CI, confidence interval; PRR, proportional reporting ratio; χ^2^, Chi-Square; IC, information component; IC025, the lower limit of the 95% CI of the IC; *, unexpected signals that were not listed in the drug label.

Supplementary Table 18. The full results of Preferred Terms for Combination Therapy reported by medical professionals: A FAERS Database Study.

| **Preferred Terms** | **Cases** | **ROR(95%Cl)** | **PRR(χ2)** | **IC(IC025)** | **adjusted *P* value** |
| --- | --- | --- | --- | --- | --- |
| Neutropenia | 2616 | 2.55(2.44-2.67) | 2.49(1653.07) | 1.03(0.96) | 0 |
| Fatigue | 2427 | 1.62(1.55-1.7) | 1.6(436.71) | 0.55(0.49) | 2.31357E-93 |
| Nausea | 1736 | 1.12(1.07-1.18) | 1.12(19.13) | 0.14(0.06) | 0.040346926 |
| White Blood Cell Count Decreased | 1475 | 2.01(1.89-2.13) | 1.99(542.77) | 0.79(0.71) | 2.2613E-116 |
| Alopecia | 972 | 1.83(1.7-1.97) | 1.82(272.79) | 0.69(0.59) | 1.25394E-57 |
| Leukopenia | 910 | 2.43(2.25-2.63) | 2.41(528.92) | 0.99(0.88) | 2.7077E-113 |
| Anaemia | 873 | 1.41(1.3-1.51) | 1.4(80.91) | 0.4(0.29) | 8.90154E-16 |
| Arthralgia | 760 | 1.81(1.66-1.96) | 1.8(204.99) | 0.68(0.56) | 7.54399E-43 |
| Decreased Appetite | 701 | 1.24(1.14-1.35) | 1.24(26.66) | 0.26(0.14) | 0.000847968 |
| Thrombocytopenia | 623 | 1.39(1.27-1.52) | 1.39(54.3) | 0.39(0.26) | 6.44547E-10 |
| Pain | 598 | 1.27(1.17-1.39) | 1.27(28.45) | 0.29(0.16) | 0.000341091 |
| Dizziness | 559 | 1.46(1.33-1.6) | 1.45(63.38) | 0.44(0.31) | 6.57411E-12 |
| Constipation | 545 | 1.39(1.26-1.52) | 1.38(46.6) | 0.39(0.25) | 3.25251E-08 |
| Neutrophil Count Decreased | 487 | 1.48(1.34-1.63) | 1.47(59.08) | 0.46(0.31) | 5.8762E-11 |
| Hot Flush | 466 | 2.48(2.22-2.77) | 2.47(284.6) | 1.02(0.86) | 4.23371E-60 |
| General Physical Health Deterioration* | 454 | 1.79(1.61-1.99) | 1.79(119.6) | 0.67(0.52) | 3.41999E-24 |
| Back Pain | 449 | 1.32(1.19-1.46) | 1.32(27.7) | 0.33(0.18) | 0.000512872 |
| Pain In Extremity | 373 | 1.37(1.22-1.53) | 1.37(29.66) | 0.37(0.21) | 0.000191471 |
| Bone Pain | 332 | 1.47(1.3-1.66) | 1.47(39.53) | 0.46(0.28) | 1.25261E-06 |
| Haemoglobin Decreased | 322 | 1.58(1.39-1.78) | 1.57(52.87) | 0.53(0.35) | 1.44821E-09 |
| Alanine Aminotransferase Increased | 320 | 1.58(1.4-1.79) | 1.58(53.29) | 0.54(0.36) | 1.16821E-09 |
| Electrocardiogram Qt Prolonged | 317 | 3.84(3.33-4.42) | 3.82(394.25) | 1.42(1.22) | 8.21275E-84 |
| Urinary Tract Infection | 309 | 1.42(1.25-1.61) | 1.42(30.69) | 0.42(0.23) | 0.000115206 |
| Aspartate Aminotransferase Increased | 309 | 1.61(1.42-1.83) | 1.61(55.26) | 0.56(0.37) | 4.34241E-10 |
| Blood Creatinine Increased | 300 | 2.3(2.01-2.63) | 2.3(156.18) | 0.94(0.75) | 4.13755E-32 |
| Red Blood Cell Count Decreased | 283 | 2.48(2.16-2.86) | 2.48(173.69) | 1.02(0.82) | 6.59283E-36 |
| Insomnia | 267 | 1.53(1.34-1.75) | 1.53(38.44) | 0.5(0.3) | 2.255E-06 |
| Dry Skin | 266 | 1.64(1.43-1.88) | 1.63(50.8) | 0.58(0.38) | 4.25085E-09 |
| Nasopharyngitis | 265 | 1.97(1.71-2.26) | 1.96(93.13) | 0.78(0.57) | 2.32056E-18 |
| Full Blood Count Abnormal* | 252 | 3.04(2.61-3.54) | 3.03(223.48) | 1.21(0.99) | 1.06677E-46 |
| Hepatotoxicity | 225 | 2.26(1.93-2.64) | 2.25(112.37) | 0.92(0.7) | 1.56103E-22 |
| Full Blood Count Decreased* | 214 | 2.71(2.3-3.19) | 2.7(155.29) | 1.1(0.87) | 7.42973E-32 |
| Depression | 209 | 1.87(1.6-2.19) | 1.87(63.75) | 0.73(0.5) | 6.45883E-12 |
| Abdominal Discomfort | 201 | 1.48(1.26-1.73) | 1.48(24.49) | 0.46(0.23) | 0.002921002 |
| Weight Increased | 190 | 1.87(1.58-2.2) | 1.86(57.28) | 0.72(0.48) | 1.72912E-10 |
| Gamma-Glutamyltransferase Increased | 186 | 2.17(1.83-2.57) | 2.16(84.28) | 0.88(0.63) | 2.2013E-16 |
| Atrial Fibrillation* | 175 | 1.62(1.37-1.92) | 1.62(32.38) | 0.57(0.32) | 5.26068E-05 |
| Transaminases Increased | 169 | 1.73(1.45-2.05) | 1.72(39.42) | 0.64(0.38) | 1.48057E-06 |
| Renal Failure* | 166 | 1.5(1.26-1.78) | 1.5(21.76) | 0.48(0.23) | 0.012201962 |
| Sleep Disorder* | 153 | 3.02(2.48-3.67) | 3.01(134.16) | 1.21(0.92) | 3.39444E-27 |
| Memory Impairment* | 145 | 1.66(1.38-2) | 1.66(29.64) | 0.6(0.32) | 0.000220018 |
| Liver Function Test Increased | 141 | 2.05(1.69-2.48) | 2.05(55.41) | 0.82(0.54) | 4.79316E-10 |
| Blood Alkaline Phosphatase Increased | 136 | 1.61(1.33-1.95) | 1.61(24.63) | 0.56(0.28) | 0.002881723 |
| Feeling Abnormal* | 134 | 1.53(1.26-1.85) | 1.53(19.24) | 0.5(0.22) | 0.046227777 |
| Dyspnoea Exertional* | 132 | 1.74(1.43-2.11) | 1.74(31.58) | 0.64(0.36) | 8.34889E-05 |
| Hepatic Cytolysis* | 128 | 1.69(1.39-2.06) | 1.69(27.9) | 0.62(0.32) | 0.000547007 |
| Influenza | 119 | 2.12(1.71-2.61) | 2.11(50.85) | 0.86(0.55) | 5.02661E-09 |
| Lacrimation Increased | 103 | 1.64(1.32-2.05) | 1.64(20.1) | 0.58(0.26) | 0.030962224 |
| Taste Disorder | 98 | 2.49(1.96-3.16) | 2.49(60.44) | 1.02(0.67) | 4.35579E-11 |
| Spinal Pain* | 96 | 2.06(1.63-2.61) | 2.06(38.54) | 0.83(0.48) | 2.65393E-06 |
| Haematocrit Decreased | 95 | 1.86(1.47-2.34) | 1.86(28.31) | 0.72(0.38) | 0.000473144 |
| Depressed Mood* | 94 | 2.14(1.69-2.72) | 2.14(41.55) | 0.87(0.52) | 5.84506E-07 |
| Cystitis | 93 | 2.15(1.69-2.73) | 2.15(41.27) | 0.87(0.52) | 6.77862E-07 |
| Illness* | 92 | 1.89(1.49-2.4) | 1.89(28.93) | 0.74(0.39) | 0.00034738 |
| Dry Eye | 90 | 1.8(1.42-2.29) | 1.8(24.43) | 0.69(0.33) | 0.003451989 |
| Cytopenia* | 89 | 2.01(1.58-2.57) | 2.01(33.44) | 0.8(0.45) | 3.56299E-05 |
| Bronchitis | 84 | 1.89(1.47-2.41) | 1.88(26.16) | 0.73(0.37) | 0.001458604 |
| Glomerular Filtration Rate Decreased* | 81 | 3.06(2.34-4.02) | 3.06(72.88) | 1.22(0.82) | 9.44688E-14 |
| Chronic Kidney Disease* | 70 | 3.95(2.91-5.36) | 3.94(90.45) | 1.45(1) | 1.63798E-17 |
| Osteoarthritis* | 67 | 2.7(2.02-3.61) | 2.7(48.34) | 1.1(0.67) | 2.20711E-08 |
| Mean Cell Volume Increased* | 62 | 4.11(2.96-5.7) | 4.11(84.32) | 1.48(1) | 3.78024E-16 |
| Skin Hypopigmentation* | 61 | 42.98(20.57-89.81) | 42.94(289.77) | 2.55(1.92) | 3.02252E-60 |
| Nasal Congestion* | 60 | 2.58(1.9-3.5) | 2.58(39.81) | 1.06(0.6) | 1.65626E-06 |
| Laboratory Test Abnormal | 58 | 2.12(1.57-2.87) | 2.12(24.97) | 0.86(0.41) | 0.002949143 |
| Erysipelas* | 56 | 2.48(1.81-3.4) | 2.48(34.45) | 1.02(0.55) | 2.52135E-05 |
| Decreased Immune Responsiveness* | 53 | 3.51(2.49-4.95) | 3.51(58.66) | 1.35(0.84) | 1.45841E-10 |
| Fluid Retention* | 51 | 2.19(1.59-3.03) | 2.19(23.82) | 0.89(0.41) | 0.005521432 |
| Diverticulitis* | 50 | 2.18(1.58-3.03) | 2.18(23.1) | 0.89(0.4) | 0.007977162 |
| Musculoskeletal Discomfort* | 50 | 2.9(2.06-4.09) | 2.9(41.17) | 1.17(0.66) | 9.16461E-07 |
| White Blood Cell Count Abnormal* | 48 | 2.22(1.59-3.1) | 2.22(22.99) | 0.9(0.4) | 0.008584268 |
| Eastern Cooperative Oncology Group Performance Status Worsened* | 44 | 2.64(1.84-3.77) | 2.64(30.44) | 1.08(0.54) | 0.000208239 |
| Vitiligo* | 43 | 8.36(5.22-13.38) | 8.35(112.08) | 1.99(1.32) | 6.09989E-22 |
| Pelvic Pain | 42 | 2.69(1.86-3.88) | 2.69(30.14) | 1.1(0.54) | 0.000247352 |
| Vitamin D Deficiency | 41 | 4.36(2.9-6.55) | 4.36(59.8) | 1.53(0.92) | 1.0022E-10 |
| Mean Cell Haemoglobin Increased* | 37 | 2.82(1.9-4.18) | 2.82(28.89) | 1.14(0.55) | 0.000491408 |
| Blood Calcium Increased | 34 | 3.83(2.48-5.92) | 3.83(42.32) | 1.42(0.76) | 6.5533E-07 |
| Platelet Count Increased* | 34 | 2.59(1.72-3.89) | 2.59(22.7) | 1.06(0.44) | 0.011361613 |
| Gastrointestinal Infection* | 32 | 6.44(3.88-10.69) | 6.44(68.58) | 1.82(1.07) | 1.69395E-12 |
| Mood Swings* | 31 | 2.57(1.68-3.93) | 2.57(20.38) | 1.05(0.41) | 0.037840275 |
| Hepatic Cyst* | 29 | 2.72(1.75-4.24) | 2.72(21.3) | 1.11(0.43) | 0.024564932 |
| Intervertebral Disc Protrusion* | 29 | 3.89(2.42-6.24) | 3.89(36.82) | 1.44(0.71) | 1.11532E-05 |
| General Physical Condition Abnormal* | 29 | 3.14(1.99-4.95) | 3.14(27.17) | 1.25(0.55) | 0.001318916 |
| Appetite Disorder* | 27 | 2.77(1.74-4.38) | 2.76(20.4) | 1.13(0.42) | 0.039867076 |
| White Blood Cell Disorder* | 27 | 3.1(1.94-4.97) | 3.1(24.82) | 1.24(0.51) | 0.004418732 |
| Hiatus Hernia* | 26 | 3.96(2.4-6.54) | 3.96(33.75) | 1.45(0.68) | 5.47083E-05 |
| Coronavirus Infection* | 26 | 2.82(1.76-4.51) | 2.82(20.3) | 1.14(0.42) | 0.042718888 |
| Hyperuricaemia* | 24 | 3.76(2.24-6.3) | 3.75(29.1) | 1.41(0.61) | 0.000571883 |
| Sinus Disorder* | 24 | 3(1.83-4.93) | 3(20.92) | 1.21(0.44) | 0.032667396 |
| Tongue Discomfort* | 23 | 5.18(2.94-9.13) | 5.18(40.42) | 1.67(0.8) | 2.30886E-06 |
| Cutaneous Lupus Erythematosus* | 21 | 3.58(2.07-6.2) | 3.58(23.91) | 1.37(0.52) | 0.008084323 |
| Anosmia* | 20 | 6.26(3.31-11.83) | 6.26(41.85) | 1.8(0.83) | 1.33461E-06 |
| Macrocytosis* | 19 | 3.82(2.13-6.84) | 3.82(23.58) | 1.42(0.51) | 0.01017613 |
| Superficial Vein Thrombosis | 18 | 4.83(2.57-9.06) | 4.83(29.41) | 1.61(0.63) | 0.000619429 |
| Calcium Deficiency* | 16 | 7.51(3.55-15.88) | 7.51(38.69) | 1.92(0.78) | 0.001079914 |
| Body Surface Area Decreased* | 10 | 18.77(5.17-68.22) | 18.77(38.83) | 2.35(0.66) | 0.003463955 |
| Vertigo Positional* | 9 | 25.35(5.48-117.31) | 25.34(38.26) | 2.44(0.6) | 0.005134222 |
| Ophthalmic Migraine* | 7 | 39.43(4.85-320.46) | 39.42(32.77) | 2.54(0.36) | 0.038163518 |

Abbreviation: ROR, reporting odds ratio; CI, confidence interval; PRR, proportional reporting ratio; χ^2^, Chi-Square; IC, information component; IC025, the lower limit of the 95% CI of the IC; *, unexpected signals that were not listed in the drug label.
